# Integrative molecular network analysis of genetic risk factors to infer biomarkers and therapeutic targets for rheumatoid arthritis

**DOI:** 10.1371/journal.pone.0329101

**Published:** 2025-08-21

**Authors:** Sakhaa Alsaedi, Katsuhiko Mineta, Naoto Tamura, Xin Gao, Takashi Gojobori, Michihiro Ogasawara

**Affiliations:** 1 Computer Science, Division of Computer, Electrical and Mathematical Sciences and Engineering (CEMSE), King Abdullah University of Science and Technology (KAUST), Thuwal, Saudi Arabia; 2 Center of Excellence on Smart Health, King Abdullah University of Science and Technology (KAUST), Thuwal, Kingdom of Saudi Arabia; 3 Center of Excellence for Generative AI, King Abdullah University of Science and Technology (KAUST), Thuwal, Kingdom of Saudi Arabia; 4 College of Computer Science and Engineering (CCSE), Taibah University, Madinah, Saudi Arabia; 5 Research Organization for Nano & Life Innovation, Waseda University, Shinjuku, Tokyo, Japan; 6 Department of System Engineering, Graduate School of Science and Engineering, Shizuoka Institute of Science and Technology, Aoi, Shizuoka, Japan; 7 Marine Open Innovation Institute (MaOI), Shimizu, Shizuoka, Japan; 8 Department of Internal Medicine and Rheumatology, Juntendo University, Tokyo, Japan; 9 Biological and Environmental Sciences and Engineering, King Abdullah University of Science and Technology (KAUST), Thuwal, Saudi Arabia; 10 Marine Open Innovation Institute (MaOI), Shizuoka, Japan; 11 Department of Life Sciences, National Cheng Kung University, Tainan, Taiwan; Sungkyunkwan University - Suwon Campus: Sungkyunkwan University - Natural Sciences Campus, KOREA, REPUBLIC OF

## Abstract

**Background:**

Understanding the interplay between genetic risk factors and molecular pathways in rheumatoid arthritis (RA) is essential for developing effective treatments. This study aims to utilize genetic risk factors of RA and identify their key pathways and potential therapeutic targets through an integrated multi-omics approach.

**Methods:**

We developed a computational pipeline to construct a knowledge graph that combines genetic risk factor molecular networks with multi-omics enrichment analysis to estimate potential therapeutic target for RA. Genetic risk factors were extracted from the literature, curated, and annotated. Molecular interaction networks were constructed based on these genetic risk factors and their neighboring proteins. Enrichment analysis was performed to identify significantly impacted biological processes and pathways. Multi-omics knowledge graph was used to prioritize candidates potential therapeutic target for RA.

**Results:**

Our analysis identified 35 significant genes associated with RA as potential therapeutic targets and biomarkers, categorized into three pathways: Cytokine Regulation and Production, Hematopoietic or Lymphoid Organ Development, and Myeloid Cell Differentiation. Among these, 25 genes were classified as risk genes, while 10 were neighboring genes. We identified nine novel risk proteins (RELA, ETS1, NFATC1, BATF, LCK, PIK3R1, PRKCB, RASGRP1,and FYN) as potential therapeutic targets for RA and they significantly contribute in the disease pathogenesis.

**Conclusion:**

This study provides a comprehensive integrative molecular network and knowledge graph analysis of genetic risk factors in RA, offering a solid framework for integrating multi-omics data in RA research. These findings may contribute to more accurate clinical decision-making and the development of targeted treatment regimens. Additionally, this study highlights the importance of inferring hidden relationships across networks based on disease associations and functional similarities, further enhancing our understanding of RA pathogenesis.

## 1 Introduction

Rheumatoid arthritis (RA) is a chronic, systemic autoimmune disease characterized by inflammation of the synovial joints, leading to progressive joint destruction, pain, and disability [1, 2]. It is also associated with conditions such as metabolic diseases [3, 4], neurological disorders [5–7], and infectious diseases [8], indicating a complex interplay between the immune system and overall health [9]. Despite significant research advancements, the precise etiology and pathogenesis of RA remain incompletely understood. It is widely accepted that genetic predisposition, environmental triggers, and immune dysregulation collectively drive RA pathogenesis [10–15]. Among these factors, genetic susceptibility plays a critical role in both disease onset and progression [16, 17]. There is a growing body of evidence suggesting that understanding genetic risk factors is essential for gaining deeper insights into the complexity of RA and how these genetic associations contribute to other diseases.

Advances in genomic technologies have facilitated the identification of numerous genetic risk factors associated with RA and the characterization of their molecular mechanisms in disease development, contributing to better disease management [17].

In genetic variant association studies, several large-scale meta-analyses of Genome-Wide Association Studies (GWAS) data have expanded our understanding of RA heritability by uncovering novel single nucleotide polymorphisms (SNPs) in immune-related genes that confer an increased risk for RA [18]. For instance, a multi-ancestry GWAS meta-analysis identified 34 novel genetic markers influencing RA risk, emphasizing the importance of diverse populations in genetic research [19]. Additionally, a cross-trait GWAS analysis revealed shared genetic loci between RA and other autoimmune diseases, suggesting common pathways in autoimmune disease pathogenesis [20].

Although GWAS studies have successfully identified genetic variants associated with RA, their functional implications in disease risk and complications remain largely unclear [21]. Therefore, understanding how these variants influence biological pathways, immune responses, and disease mechanisms is crucial for the development of targeted therapies and precision medicine strategies [22]. Identifying and characterizing the biological pathways and molecular mechanisms influenced by these genetic risk factors are essential for developing targeted therapies and improving disease management [17]. This research direction focuses on identifying molecular mechanisms and predictive biomarkers while exploring potential therapeutic drugs through integrative multi-omics analyses. For example, Lu Xiao *et al*. (2022) applied integrative biomarker identification methods to provide insights into RA [23], while Caifang Shen *et al*. (2024) utilized bioinformatics approaches to identify significant biomarkers for RA diagnosis [24].

Despite advances in elucidating RA’s molecular pathways and identifying therapeutic targets [17, 25, 26], significant limitations remain. Research often isolates systems like the cytokine network, overlooking the interplay between biological pathways [27, 28]. Studies on genetic risk factors frequently lack integration with pathway analyses, limiting understanding of genetic variations’ influence on RA [29–31]. While gene expression studies identify immune response pathways, the absence of comprehensive genetic factor analysis restricts insights into how risk genes contribute to RA pathogenesis [10, 32]. Comprehensive network analysis is needed to understand these risk variants’ functional implications [33], identify the biological pathways they influence, and develop targeted therapies for improved disease management [10, 34].

This study employs an integrated approach combining genetic risk factor analysis with multi-omics enrichment and network analysis to construct a comprehensive knowledge graph of genetic risk factors in RA to identify key pathways and potential therapeutic targets in RA. We construct molecular networks based on identified genetic risk factors and their neighboring proteins to uncover critical molecular interactions. Additionally, we perform a comprehensive enrichment analysis to highlight the most significantly impacted biological processes and pathways. Our objectives are threefold: (1) to identify core risk genes and pathways significantly associated with the complexity of RA; (2) to identify novel potential therapeutic targets for RA; and (3) to provide a foundation for future research aimed at validating these findings through experimental studies. By achieving these objectives, we aim to advance the understanding of RA pathogenesis and contribute to the development of targeted therapeutic strategies for improved patient outcomes [33].

## 2 Materials and methods

**Fig 1:**
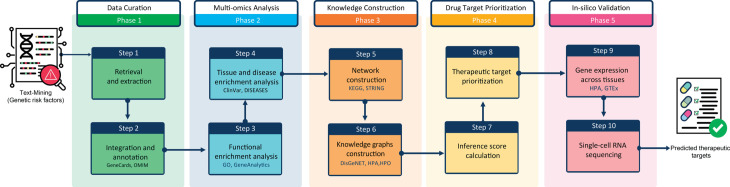
Overview of implemented computational workflow for analysis of genetic risk factors of RA. Schema of the computational molecular pipeline for analysis of genetic risk factors associated with RA. The implemented pipeline consists of five stages and phases: Phase 1:retrieving and annotating a list of risk genetic factors, Phase 2: enrichment analysis of the curated dataset of genetic risk factors, Phase 3: constructing molecular network interactions and graphs for the identified genetic risk factors, Phase 4: predicting protein therapeutic targets for RA, and Phase 5: in silico validation of novel RA therapeutic targets.

We developed a computational pipeline to analyze genetic risk factors across multiple omics levels and construct a multi-omics knowledge graph to estimate potential therapeutic target scores for RA using integrative molecular network analysis. This pipeline integrates various database APIs and bioinformatics packages across five key stages: (1) data curation of RA genetic risk factors, (2) comprehensive enrichment analysis based on molecular function, biological process similarity, tissue and cell-type specificity, molecular system similarity, and disease association, (3) construction of risk factor knowledge graphs, (4) protein prioritization and therapeutic target scoring, and (5) in silico validation of predicted therapeutic targets for RA (Fig 1).

### 2.1 Data curation of genetic risk factors for RA

In the data curation, which is the first step in our computational workflow, the phase employed for systematically extracting and annotating genetic risk factors associated with RA from various omics levels.

#### 2.1.1 Text-mining-based data sources for RA.

Instead of direct access to raw GWAS datasets, which require extensive computational resources and ethical considerations, this study employed text mining to systematically extract validated RA-associated genetic variants from peer-reviewed GWAS literature in PubMed. It did not use UK Biobank summary statistics or other GWAS databases directly but compiled genetic risk factors for RA from published studies. This approach enables automatic integration of reported risk variants and genes while ensuring data transparency and reliability.

#### 2.1.2 Data retrieval and extraction.

In the first phase of our data preparation, we utilized PubTator [35], a web-based text mining tool, to systematically extract genetic risk factors for RA across different omics levels. PubTator facilitated a comprehensive search of the published literature in English, focusing on relevant research articles and reviews. PubTator was selected for its natural language processing capabilities and PubMed integration, enabling structured extraction of gene-disease associations. Unlike BioBERT or LitVar, it provides broad coverage with minimal redundancy by integrating multiple ontologies. The search strategy leveraged controlled vocabulary and Boolean operators to identify studies investigating genetic susceptibility to RA, including queries such as "rheumatoid arthritis AND genetic risk factors" and "host genetic variant AND rheumatoid arthritis." Our search parameters were restricted to peer-reviewed publications indexed in PubMed and published between January 1, 2018, and December 28, 2023. Preprints were excluded due to their lack of peer review. At the end of this phase, we extracted unfiltered genetic risk factors (SNPs, genes, or proteins) of RA, which were saved in a list for the next phase of data curation and annotation.

#### 2.1.3 Data integration and annotation.

As part of the first phase, we applied quality control and filtering to the genetic risk factors by removing duplicates and selecting GWAS studies with sample sizes of ≥ 500 individuals from Asian or European populations, with significant odds ratios above 0.7. Following quality control and manual validation, we implemented a bioinformatics annotation workflow integrating more than four biomedical and genetic databases [36–40] to annotate 25 genetic features describing genetic risk factors. These features include genetic variant ID, chromosomal loci, associated gene, odds ratio, related phenotypes and diseases, and references. The workflow utilized the myVariants package [37] for genetic variant annotation due to its integration of dbSNP [36], ClinVar [41], and Ensembl [39]. Its real-time API queries minimize computational overhead and ensure access to the latest variant information. Each variant associated with RA is termed a risk polymorphism, while its corresponding gene and protein are referred to as a risk gene and risk protein, respectively.

#### 2.1.4 Potential data biases and mitigation strategies.

Despite robust data curation, biases may arise from text-mining limitations in detecting genetic risk factors that are less explicitly described or weakly highlighted in the literature. While PubTator efficiently extracts genetic variants, its reliance on published annotations may overlook less-documented genetic risk factors. Additionally, population-specific biases in GWAS data can limit the generalizability of findings. To address these biases, we cross-referenced extracted genetic risk factors with curated databases such as ClinVar, DisGeNET, and the GWAS Catalog for comprehensive validation. We also included multi-population GWAS studies to reduce population bias; however, most retrieved data were from European cohorts, emphasizing the need for broader representation in future research.

### 2.2 Enrichment analysis of genetic risk factors for RA

In the second stage, we performed a comprehensive enrichment analysis to characterize the biological significance of RA genetic risk factors, including functional annotation, tissue-cell specificity, and disease association.

#### 2.2.1 Process of functional enrichment analysis.

To characterize the functional role of RA risk genes, we performed gene set enrichment analysis using the Gene Ontology (GO) classification system. This approach identifies molecular functions and biological pathways enriched in the risk gene set. GOATools package [40] was selected to apply the analysis for its computational efficiency, flexibility, and rigorous statistical framework, implementing Fisher’s exact test with multiple testing correction (Benjamini-Hochberg FDR adjustment) for accurate significance estimation. This step provides biological interpretation and insights into the molecular functions and biological processes contributing to the complexity of RA.

#### 2.2.2 Process of tissue-cell type enrichment analysis.

We used the WebCSEA bioinformatics method to analyze the tissue and cell-type specificity of 196 risk genes [42]. A graphical gene-set enrichment package was employed to investigate the ontologies of tissues and compartments associated with these risk genes, using an FDR cutoff of 0.05 [40]. The WebCSEA tool conducted tissue and cell-type specificity enrichment analysis on the risk gene list using a curated dataset comprising 111 scRNA-seq panels and 1,355 TCs from 61 human tissues across 11 organ systems [42]. The FDR threshold of 0.05 was chosen to maintain a balance between statistical stringency and biological relevance. A lower threshold (e.g., 0.01) would eliminate potentially meaningful tissue-specific associations, whereas a higher threshold would increase the risk of false positives. This threshold is consistent with standard practices in functional enrichment analyses, where a moderate FDR cutoff is necessary to avoid excessive filtering of tissue-specific signals while maintaining statistical rigor [43, 44].

#### 2.2.3 Process of diseases enrichment analysis.

To map genetic risk factors related to RA and other associated diseases, we employed a disease similarity mapping approach to identify shared risk genes and their neighboring genes across related diseases. The associations within disease-gene networks were analyzed to uncover common biological pathways, utilizing biomedical databases such as ClinVar [41], Human Phenotype Ontology (HPO) [45], DISEASES, the Alliance Disease Database, and OMIM [46]. Furthermore, we integrated our curated gene-disease dataset of RA genetic risk factors with biomarkers and therapeutic targets from DisGeNET [47] and GeneCard [38] to identify potential therapeutic drugs for RA.

### 2.3 Construction of molecular networks and knowledge graphs for RA genetic risk factors

To systematically explore the molecular landscape of RA, we constructed protein-protein interaction (PPI) networks and a multi-layered knowledge graph, integrating diverse biological data sources to uncover functional associations and potential therapeutic targets.

#### 2.3.1 Construction of protein-protein interaction networks.

In the third stage, we constructed PPI networks for genetic risk factors using the STRING 11.5 database [48], accessed on February 20, 2023, with a confidence threshold of ≥0.9 . We calculated combined protein interaction confidence scores by summing all STRING confidence values and applying Min-Max normalization. Only interactions with confidence scores ≥0.9 were retained for further analysis. To enhance network visualization, we limited the number of interactions displayed in the network outputs, prioritizing top interactions for each risk protein based on the highest combined confidence score. We then used Cytoscape [49] to integrate the data and generate PPI networks, grouping unconnected networks based on the similarity of their molecular functions. Finally, we clustered the constructed networks based on the results of previous enrichment analyses, considering disease associations, common tissues, shared components, and molecular functions.

#### 2.3.2 Construction of knowledge graphs.

Next, we constructed a multi-layered knowledge graph based on the largest PPI network of genetic risk factors to mitigate graph sparsity. It integrates data from multiple biological sources, including the Proteomic Drug Atlas, GWAS Catalog, GeneAnalytics, Human Gene Atlas, DisGeNET, STRING, KEGG, Reactome Pathways Database [47, 48, 50, 51], and the Kinase Library, enabling the identification of proteins functionally linked to RA and related diseases. The knowledge graph comprises nodes representing genes, proteins, diseases, and pathways. Edges define relationships based on co-expression patterns, transcriptional regulation, protein-protein interactions, and shared disease phenotypes. We applied network-based clustering to identify highly interconnected subnetworks that reveal disease mechanisms and therapeutic targets. The graph was further refined using semantic integration techniques to align heterogeneous data sources and infer missing associations, enhancing predictive analyses of RA-related molecular interactions.

### 2.4 Calculation of inference score for potential therapeutic targets

In the fourth stage, we developed a scoring framework to quantify the relevance of each protein as a therapeutic target for RA. This framework integrates molecular network analysis and knowledge graph modeling to enhance prediction accuracy.

#### 2.4.1 Calculation of enriched scores.

Four enriched scores were estimated to calculate the final inference score, determining how likely a protein is to be a therapeutic target based on comparative and integrative analysis from previous steps. These scores are defined as follows: *S*_*d*_ represents the *Disease Mapping Score*, reflecting RA-associated protein-disease connections from curated sources. *S*_*m*_ is the *Molecular Interaction Score*, derived from PPI and pathway analysis. *S*_*b*_ corresponds to the *Biomedical Knowledge Score*, integrating transcriptomic and proteomic annotations. *S*_*k*_ is the *Knowledge Graph (KG) Score*, quantifying the strength of inferred relationships within the constructed knowledge graph. Each score component was calculated as follows:

$$S_{d}=\frac{\left|D_{p}\right|}{\left|D\right|}$$ (1)

where |*D*_*p*_| represents the number of studies reporting the protein’s association with RA, and |D| is the total number of studies in the curated dataset.

$$S_{m}=\alpha C_{d}+\beta C_{b}+\gamma C_{c}$$ (2)

where *C*_*d*_, *C*_*b*_, and *C*_*c*_ denote degree centrality, betweenness centrality, and closeness centrality, respectively, within the RA-related PPI network, and *α*, *β*, and *γ* are scaling factors.

$$S_{b}=\frac{|E_{p}|-E_{\min}}{E_{\max}-E_{\min}}$$ (3)

where |*E*_*p*_| represents the expression level of the protein in RA patient datasets, normalized using min-max scaling.

$$S_{k}=\sum_{i=1}^{n}w_{i}R_{i}$$ (4)

where *R*_*i*_ represents the relationship strength between the protein and an RA-related entity in the knowledge graph, and *w*_*i*_ is the assigned importance weight of the relationship.

#### 2.4.2 Inference score calculation.

The inference score (*I*_*p*_) is computed by integrating four independent scores, each representing a different layer of molecular and disease-related evidence, including interactions, associations, and knowledge graphs. This score ranges from 0 to 1, with higher values indicating stronger evidence of RA association.

$$I_{p}=w_{d}S_{d}+w_{m}S_{m}+w_{b}S_{b}+w_{k}S_{k}$$ (5)

Weight coefficients were selected based on empirical validation, balancing contributions from disease associations (30%), molecular interactions (30%), biomedical knowledge (20%), and knowledge graphs (20%). This distribution ensures comprehensive integration of multi-source evidence without overemphasizing any single data layer:

$$w_{d}=0.3,\;w_{m}=0.3,\;w_{b}=0.2,\;w_{k}=0.2$$ (6)

Each score component was normalized using min-max scaling:

$$S^{\prime }=\frac{S-S_{\min}}{S_{\max}-S_{\min}}$$ (7)

To predict potential therapeutic targets, we ranked proteins based on their (*I*_*p*_) and classified them according to biological function, regulatory role, and molecular interactions. Proteins with *I*_*p*_>0.85 were identified as high-confidence targets, supported by multiple layers of evidence linking them to RA pathology. Additionally, network-based enrichment analysis was applied to assess their involvement in RA-related pathways.

### 2.5 In silico validation of predicted therapeutic therapeutic targets

To validate the predicted therapeutic targets, we conducted in-silico analysis using data from the Human Protein Atlas (HPA), GTEx, and single-cell RNA sequencing (scRNA-seq) datasets. GTEx provides gene expression data across various tissues, while HPA offers protein-level validation through immunohistochemistry. scRNA-seq datasets add single-cell resolution, allowing us to examine expression patterns in immune cells relevant to RA. We prioritized proteins with high expression in relevant tissues as potential therapeutic candidates. To further confirm their relevance, we consulted RA specialists and cross-referenced findings with biomarkers and therapeutic targets from DrugBank [52] and medical literature. This in-silico validation approach enhances confidence in the identified therapeutic targets and their potential therapeutic applications.

## 3 Results

### 3.1 Overview of genetic risk factors in RA

The retrieved list of genetic risk factors for RA includes 279 SNPs associated with the condition. However, only 60% of the reviewed articles provided odds ratios for the reported significant risk variants. These SNPs were annotated and integrated with public biomedical and genetic databases to create a dataset comprising 196 genes, 158 of which are protein-coding. The genetic profiles in this curated dataset provide descriptive information on RA-associated risk polymorphisms. A shortlist of these genetic risk factors is presented in Table 1. The full curated dataset is publicly accessible in the supplementary materials (1–3). Summary statistics and processed results can be provided upon reasonable request.

**Table 1:** Top 30 genetic risk factors related to rheumatoid arthritis.

Risk variants	Chromosome	Risk gene	OR	PubMed ID (PMID)
rs2476601	1	PTPN22	1.81	15208781, 20453842
rs9557321	13	CLYBL	1.73	24532677
rs113066392	7	GTF2IRD1-NCF1	1.43	33310728, 28135245, 27272985
rs138193887	11	CUL5	1.21	33310728, 35088123, 22446963
rs3753389	1	CD244	1.30	18794858
rs909685	22	SYNGR1	1.14	23143596, 33310728
rs11933540	4	RBPJ	1.15	24390342, 23143596, 20453842
rs16903108	8	PVT1	1.15	35088123, 23143596
rs911760	9	PLGRKT	1.15	35088123
rs940825	7	AGR3-AHR	1.13	35088123
rs2867461	4	ANXA3	1.13	22446963
rs11574914	9	CCL21	1.12	35088123, 23143596, 22446963, 20453842
rs13031237	2	REL	1.12	19503088
rs6681482	1	TNFSF4	1.12	24532676, 35088123, 24390342
rs705700	12	CDK2	1.09	24390342, 33310728
rs34480360	16	ZNF689	1.09	33310728, 35088123
rs2300373	21	IFNGR2	1.09	35088123, 33310728, 23143596
rs5754104	22	UBE2L3-YDJC	1.09	23143596, 35088123, 33310728
rs1427749	12	SCAF11	1.08	35088123
rs9979383	21	RUNX1	1.08	23143596, 35088123, 33310728, 23143596
rs866205108	18	TNFRSF1A	1.10	35088123
rs1885013	14	RAD51B	1.11	24390342, 33310728
rs11375064	17	KSR1	0.93	23143596, 35088123, 24390342
rs11777380	8	CCN4	0.92	35088123
rs2671692	10	WDFY4	0.92	23143596, 22446963,35088123
rs660442	11	BAD	0.90	33310728, 35088123
rs6032662	20	CD40	0.90	19898481,35088123, 22446963, 23143596
rs6495979	15	RasGRP1	0.88	23143596, 35088123, 24390342, 33310728
rs10790268	11	CXCR5	0.87	24390342
rs1571878	6	CCR6	0.86	23143596, 22446963, 20453842

### 3.2 Enrichment analysis of genetic risk factors in RA

The results of the multi-omics enrichment analysis of genetic risk factors in RA, including biological processes, molecular functions, cell-type specificity, and disease associations are summarized in the following sections.

#### 3.2.1 Biological processes enrichment analysis.

**Fig 2:**
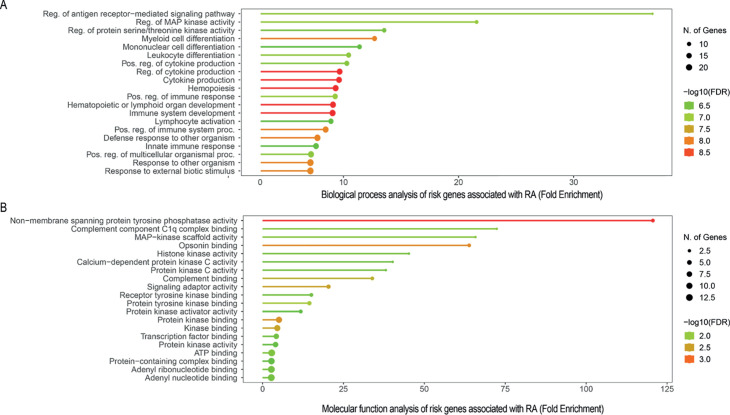
Biological process and molecular function analysis of genetic risk factors of RA. Enrichment analysis of the molecular function and biological processes related to the genetic risk factors for rheumatoid arthritis. Fold enrichment was calculated by dividing the proportion of genes in the list of risk genes that are involved in a pathway by the equivalent percentage in the background. The FDR indicates how probable enrichment is due to chance alone; higher scores indicate the enrichment is biologically relevant. Fold enrichment reveals the extent to which genes significant to a specific pathway are overrepresented.

In the biological processes, three main pathways are significantly impacted by genetic risk factors: Cytokine Regulation and Production, Hematopoietic or Lymphoid Organ Development, and Myeloid Cell Differentiation, as shown in Fig 2A. Risk genes such as IRAK1, UBASH3A, AIM2, and CD40 are involved in inflammatory responses and cytokine regulation and production. The Hematopoietic or Lymphoid Organ Development pathway is essential for the development and function of immune cells. Notable risk genes in this pathway include SLC8A3, FLT3, TGF-*β*1, RasGRP1, PLD4, and AIRE, which play important roles in healthy immune responses in RA. A subset of the hematopoietic pathway focuses specifically on the differentiation of myeloid cells, which are crucial for inflammation and tissue damage in RA. Key genes involved include SH2B3, TNFRSF1A, CDK2, MED1, PRKCB, KMT2B, and ZFP361, with some, such as TNFRSF1A and SH2B3, being potential therapeutic targets. Interestingly, seven risk genes (RUNX1, IRF8, BATF, PTPN11, PTPN22, PTPN2, and IFI-16) intersect all three main pathways. These risk genes appear to play a central role in influencing all three pathways, suggesting their potential importance in the complex interplay between inflammation and immune cell function in RA.

#### 3.2.2 Molecular function enrichment analysis.

In terms of molecular function, Fig 2B demonstrates significant enrichment of non-membrane spanning protein tyrosine phosphatase activity with a −log(FDR)=3, emphasizing its role in immune system responses and the hematopoietic system. Four RA risk genes - BATF, PTPN11, PTPN2, and PTPN22 - play crucial roles in this activity, impacting cytokine production, lymphoid organ development, and myeloid cell differentiation. For example, PTPN2, known for its anti-inflammatory properties, emerges as a potential therapeutic target for both RA and pancreatic adenocarcinoma (PAAD) due to its ability to moderate inflammation and reduce tumor malignancy [53, 54]. Similarly, PTPN22, involved in T cell signaling, serves as both a biomarker for disease predisposition and a potential therapeutic target for RA [53, 55]. Furthermore, BATF regulates immune activation and T-cell memory, underlining its importance in immune responses [56, 57].

#### 3.2.3 Cell-type specific enrichment analysis.

**Fig 3:**
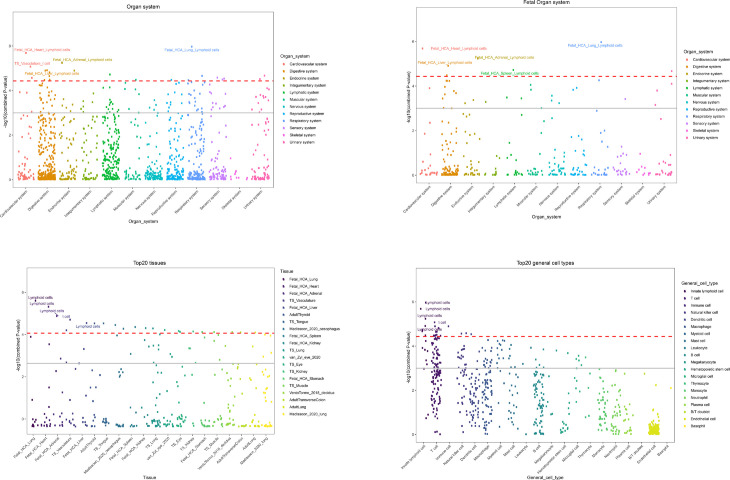
Cell-type specific enrichment analysis of genetic risk factors of RA. This figure presents the top enriched tissues and cell types based on cell-type-specific enrichment analysis of genetic risk factors for rheumatoid arthritis. The top left plot shows organ system enrichment, reflecting RA’s autoimmune nature. The top right plot highlights tissue-specific enrichment, emphasizing fetal and adult immune-related tissues. The bottom plot presents cell-type enrichment, identifying key immune cells (T cells, B cells, macrophages, and dendritic cells) as major contributors to RA pathogenesis. The bottom right plot illustrates immune lineage-specific enrichment, showing the relationships of RA-associated immune cells in adaptive and innate immunity

Fig 3 shows the cell-type specific enrichment analysis that indicates the genetic risk factors for RA are highly enriched in the cardiovascular, respiratory, and digestive systems, highlighting these organ systems as crucial targets for improving RA treatment efficacy. The digestive system, particularly the intestinal tract, plays a significant role in RA pathogenesis [58]. Here, RA risk genes are expressed in fetal chorioamnionitis lung and heart tissue, and in the vasculature, affecting lymphoid cell and T cell memory functions, which are key in RA development. This suggests that targeting these tissues in early interventions may help prevent RA onset. Tissue-specific analysis reveals significant expression of genetic risk factors in immune-related tissues, especially lymphoid cells, underscoring the importance of immune cell regulation in RA treatment. General cell type analysis identifies diverse immune cells, including antigen-presenting cells such as dendritic cells, which are crucial for T cell activation and autoantibody production by B cells. The role of T cells is emphasized, with drugs such as Abatacept and Tacrolimus showing therapeutic potential in RA. Microglia, the central immunocompetent cells of the nervous system, are highly enriched in cytokine production and regulation. Typically associated with the brain and spinal cord, microglia are implicated in synovitis of peripheral joints, contributing to RA-related pain. This suggests that microglial activation, a secondary change due to peripheral joint inflammation, may be important for managing RA symptoms.

#### 3.2.4 Disease enrichment analysis.

**Fig 4:**
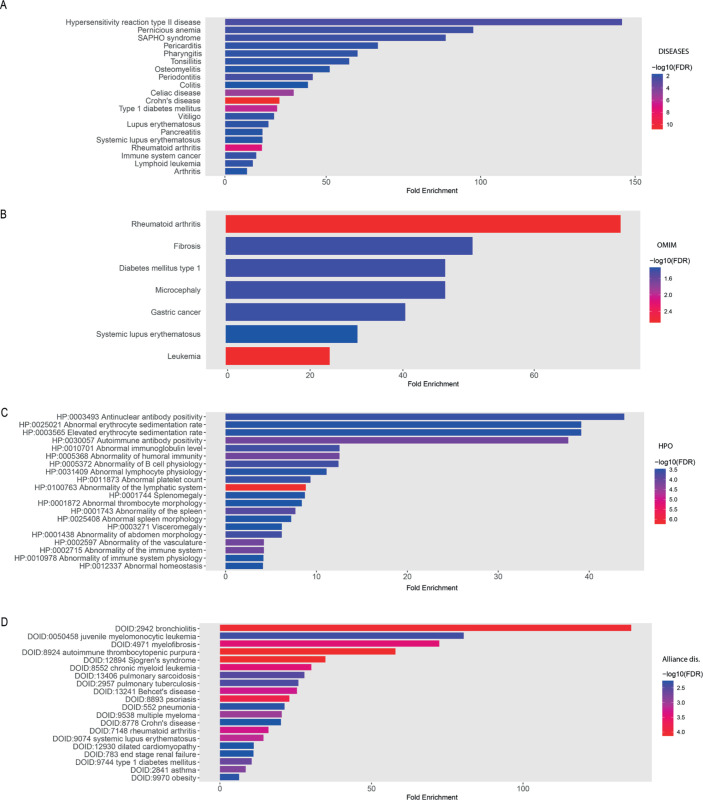
Disease enrichment analysis of genetic risk factors of RA. Disease mapping and relations based on enrichment analysis of the identified genetic risk factors for rheumatoid arthritis. (A) DISEASES database: gene-disease associations from text-mining and experiments. (B) OMIM: inherited disorders and clinically relevant mutations. (C) HPO: phenotype-based links between RA risk genes and patient traits. (D) Alliance Disease: cross-species disease models and functional genomics

We performed a disease enrichment analysis to identify the most enriched diseases associated with the risk genes of RA, as illustrated in Fig 4. Our disease mapping indicates that Crohn’s disease is highly associated with RA (Fig 4A). Seven shared risk genes (CD40, PTPN22, CD226, BATF, PRKCB, RasGRP1, and PTPN2) are involved in cytokine pathways linked to Crohn’s disease. Additionally, Fig 4B–4C links chronic myeloid leukemia to several RA risk genes (CD40, TGF-*β*1, SH2B3, and IL1RN). Phenotype analysis revealed significant associations between RA risk genes and lymphatic system abnormalities. Three overlapping genes (RUNX1, FLT3, PTPN11) connect to pathways involved in abnormal cell growth (hematopoiesis). RA risk genes also show strong associations with autoimmune comorbidities, including bronchiolitis, Sjögren’s syndrome, and psoriasis (Fig 4D). For example, CD40, CRP, and IL1RN are involved in cytokine production pathways, which play a crucial role in regulating inflammatory responses. Dysregulation of these pathways contributes to excessive immune activation in both RA and bronchiolitis, a condition characterized by airway inflammation and immune cell infiltration. Similarly, SH2B3, CD40, and CRP are linked to Sjögren’s syndrome, diabetes mellitus, and celiac disease, reinforcing the genetic connections between RA and other autoimmune diseases. SH2B3 encodes a signaling adaptor that modulates T-cell activation and immune homeostasis, and its variants have been implicated in autoimmune susceptibility across multiple conditions. CD40, a co-stimulatory protein, is critical in B-cell activation and autoantibody production, contributing to glandular inflammation in Sjögren’s syndrome and immune dysregulation in diabetes and celiac disease. CRP, an inflammatory biomarker, is consistently elevated in these disorders, indicating a shared inflammatory cascade underlying their pathogenesis.

### 3.3 Construction and analysis of rheumatoid arthritis protein networks

**Fig 5:**
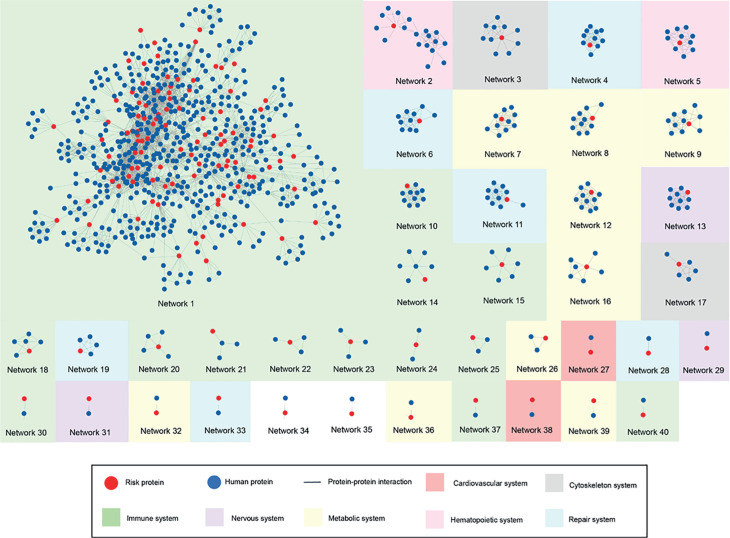
Molecular network clustering of constructed genetic risk proteins networks in RA. This figure illustrates the molecular system clustering of 40 protein-protein interaction networks. Red nodes represent genetic risk proteins associated with RA, while blue nodes indicate neighboring proteins that interact with them. Each color-coded cluster corresponds to a major molecular system, categorized by shared biological functions and disease relevance. These clusters highlight molecular similarities within functional systems and emphasize the role of each network in RA pathogenesis.

The puzzle plot in Fig 5 shows our 40 constructed PPI networks with 3462 protein interactions based on 158 risk proteins and their molecular function similarities. The green block contains thirteen networks related to the immune system. The yellow block consists of nine networks related to metabolic disorders. Six networks form the repair system, indicated by the blue block, and are involved in DNA and chromosomal processes. Lastly, Networks 13, 29, and 31, associated with the nervous system, relate to Alzheimer’s disease and psychiatric disorders. Most networks are associated with the immune and metabolic systems, but Networks 2 and 5 are related to the hematopoietic system.

Certain constructed networks have no association with the immune system. For example, Network 3 is related to the cytoskeletal system, which is affected in RA. Network 17 plays an essential role in the regulatory activities and biological processes of vascular metabolism. Moreover, Networks 23, 29, and 31 contribute to the neural system. Detailed summaries of the molecular functions and pathway analysis of the 39 remaining constructed networks are provided in the supplementary material 4. Additionally, Networks 1-5, 13, 14, and 17-16 share common pathways with the cardiovascular system and cardiovascular diseases. Networks 4, 6, 11, and 28 are related to the respiratory system.

#### 3.3.1 Significant risk proteins analysis in network 1.

Network 1, the largest network, Network 1 consists of 893 proteins, including 111 RA risk proteins, 121 direct neighbor proteins, and 661 additional non-risk proteins. It contains 2,883 PPIs, with approximately 71% of interactions involving RA risk proteins and their direct neighbors. It is mainly associated with immune, musculoskeletal, and metabolic systems. Key pathways include cytokine signaling, innate immune processes, myeloid cell differentiation, hematopoietic development, responses to infectious diseases, such as Hepatitis B, measles, and cancer-related pathways, such as leukemia, breast cancer.

**Fig 6:**
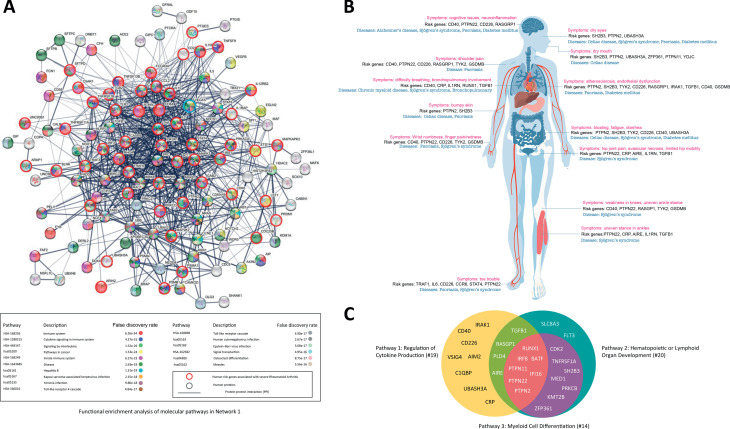
Integrative analysis of molecular network and disease association of genetic risk factors for RA. (A) Key molecular and disease pathways of genetic risk proteins for RA in Network 1. (B) Risk gene-disease mapping, showing shared risk genes between RA and other diseases. (C) Pathway interactions of significant RA risk genes, therapeutic targets, and biomarkers, identified through disease mapping and multi-omics network enrichment. This visualization highlights functional overlaps between biological pathways, demonstrating how key risk genes interact across immune regulation, cytokine production, and hematopoietic differentiation.

Fig 6A shows a subnetwork of PPI in Network 1, with red circles indicating genetic risk factors and blue circles indicating main hub proteins interacting with 80% of genetic risk factors. PADI4, a significant risk protein, is an arginine-to-citrulline converting enzyme, important for RA pathogenesis and diagnosis. Despite its role, PADI4’s interactions in Network 1 are sparse, mainly involving inflammatory cytokines and signaling, more related to pathogenesis than disease risk. In Network 1, VEGF is a hub protein that regulates inflammatory responses and is involved in cancer pathways, supporting tumor growth and chronic inflammation in RA. VEGF also contributes to the TNF signaling pathway, a therapeutic target for diseases associated with immunity and cancer.

The disease mapping analysis of RA risk genes reveals significant associations with various other diseases. Specifically, four RA risk proteins are linked to neurological diseases such as Alzheimer’s, six to psoriasis, five to respiratory diseases, nine to metabolic diseases, five to Sjogren’s syndrome, and six to bone diseases. These findings, illustrated in Fig 6B, suggest shared molecular pathways and potential therapeutic targets across multiple diseases.

The Venn diagram in Fig 6C illustrates the intersections among three main pathways involved in RA development: cytokine production regulation, hematopoietic signaling, and myeloid cell differentiation. A total of 28 genetic risk factors overlap between these pathways, including 11 identified therapeutic targets and 7 biomarkers for RA. (See Table 4)

### 3.4 Multi-omics knowledge graph analysis of RA genetic risk factors

**Fig 7:**
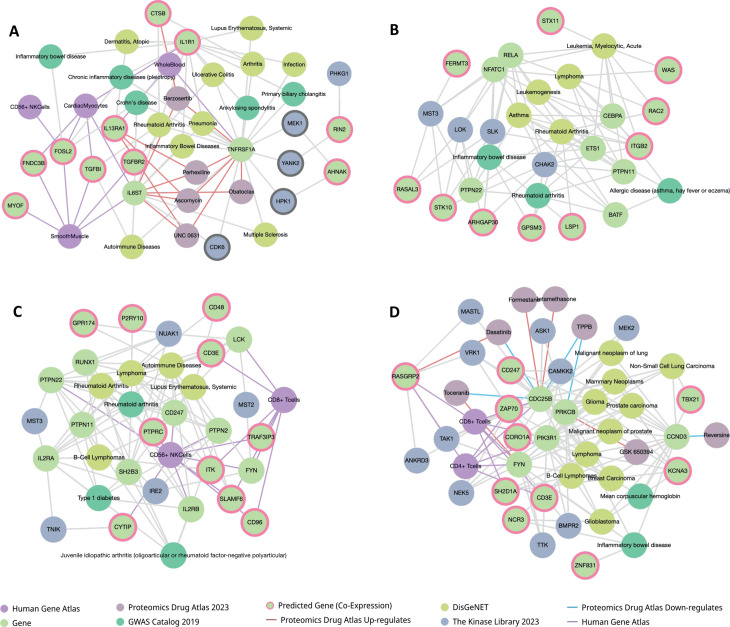
Multi-omics knowledge graph analysis of genetic risk factors in RA. The constructed knowledge graph displays RA risk proteins and their interacting non-risk proteins, integrating data from multiple databases. This analysis highlights the top 10 enriched pathways and molecular interactions, providing insights into key biological processes, signaling cascades, and potential therapeutic targets involved in RA pathogenesis.

Table 2 presents the non-risk proteins that directly interact with RA risk proteins. Fig 7 illustrates the constructed sub-knowledge graphs, highlighting the top 10 enriched pathways or terms from six key databases: Human Gene Atlas, Proteomics Drug Atlas, DiseaseGeneNet [47], KEGG, The Kinase Library, and GWAS Catalog [59]. These databases were integrated to identify significant proteins that are highly connected with RA risk proteins, aiding in the understanding of potential disease mechanisms and therapeutic targets.

**Table 2:** Molecular pathways and disease associations of interacting proteins with risk proteins in network 1.

Category	Neighboring Genes	Associated Diseases
A) Cytokine Signaling: 39 genes	IL6ST, SOCS3, TNFSF9, TRAF2, IL12A, IL12B, IL12RB1, IL1B, TNFRSF4, IFNG, IRF9, TNFRSF10A, TNFRSF10B, TNFRSF1A, IL19, IL20, IL20RA, IL22RA1, IL2RB, IL2RG, IL10RA, IL10RB, IFNGR1, IL3RA, IL5RA, TNFRSF25, TNFRSF6B, IL23A, IL1R1, IL1RAP, IL1R2, IRAK3, IFNAR1, IFNAR2, CXCL1, IL1A, IL17RC, TNFSF15, TNFSF13B	Alzheimer’s Disease, Psoriasis, Crohn’s Disease, Cancer, Diabetes, Inflammatory Diseases, Inflammatory Bowel Disease, Psoriasis, Asthma, Immunodeficiency Disorders, Various Inflammatory Diseases, Immune System Disorders, Autoimmune Diseases, Infectious Diseases, Viral Infections, Autoimmune Diseases, Psoriasis, Inflammatory Bowel Disease, Severe Combined Immunodeficiency, Hematological Malignancies, Eosinophilic Disorders, Autoimmune Diseases, Cancer, Various Inflammatory Diseases, Immune System Disorders, Viral Infections, Autoimmune Diseases, Cancer, Immune System Disorders
B) Transcription Factors: 30 genes	SPI1, EGR2, GATA2, CEBPA, BATF, FOXP3, NFATC1, MAFF, PRDM1, JUN, JUNB, FOS, ETS1, TAL1, MYB, RORA, RORC, TBX21, TBXAS1, KLF6, ELF2, RELB, TP63, MAFK, FLI1, STAT5A, STAT5B, STAT6, NF1, RELA	Leukemia, Multiple Sclerosis, Myelodysplastic Syndromes, Immunodeficiency, Charcot-Marie-Tooth Disease, Cancer, Autoimmune Diseases, Heart Disease, Diabetes, Inflammatory Diseases, Bone Diseases, Blood Disorders, Asthma, Cardiovascular Diseases
C) Immune Receptors and Signaling: 37 genes	CD247, FCER1G, FYN, LYN, NCAM1, SRC, LCK, CD22, CD8A, CD48, CD274, CD276, TNFRSF1B, CSK, PTPN11, PTPN6, PTPRCAP, PTPN1, PTPN12, PTPN18, SH2D1A, SH2D1B, SH2B3, SHC1, CD79A, CD79B, CD40LG, IL2RA, IL2RB, IL2RG, IL10RA, IL10RB, IL5RA, IL3RA, IL17RC, CXCL1, ACKR4	Immune System Disorders, Allergic Diseases, Cancer, Nervous System Diseases, Bone Diseases, Hematological Malignancies, Eosinophilic Disorders, Psoriasis, Autoimmune Diseases, Inflammatory Bowel Disease, Viral Infections, Various Inflammatory Diseases, Immune System Disorders, Severe Combined Immunodeficiency
D) Enzymes and Kinases: 35 genes	AKT1, MAP2K1, MAP2K2, MAPK1, MAPK8, MAPKAPK2, PRKCB, PRKCD, PRKACA, CDK8, CDKN2A, CDKN2B, CDKN2C, CDKN2D, CCNA1, CCNA2, CCNB1, CCND2, CCND3, CDC25B, SRC, LCK, FYN, LYN, CSK, ITK, TEC, AKT1, AKT2, PIK3CA, PIK3R1, PLCG1, PLCG2, RAF1, PTK2	Cancer, Diabetes, Neurological Disorders, Various Inflammatory Diseases, Developmental Disorders, Myelodysplastic Syndromes, Neurodevelopmental Disorders, Metabolic Disorders, Bone Diseases, Cardiovascular Diseases, Immune System Disorders

The knowledge graph integrates data from multiple sources to uncover RA-associated molecular interactions, genetic predisposition, and therapeutic targets. Human Gene Atlas identifies tissue-specific expression, while Proteomics Drug Atlas links proteins to therapeutic targets. DiseaseGeneNet and GWAS Catalog highlight shared genetic risk factors, and KEGG maps molecular pathways involved in RA. The Kinase Library focuses on phosphorylation networks essential for immune regulation. Together, these datasets provide a comprehensive view of RA pathogenesis, aiding in drug discovery and precision medicine approaches.

#### 3.4.1 Cytokine signaling pathway neighboring proteins.

The subnetwork analysis of the cytokine signaling pathway, illustrated in Fig 7A, reveals significant associations with RA and related diseases through the study of neighboring proteins of genetic risk factors. Key proteins such as TNFRSF1A, IL1R1, and IL6ST emerge as potential therapeutic targets and biomarkers for RA. TNFRSF1A is linked to a range of diseases including lupus erythematosus, arthritis, RA, infection, multiple sclerosis, autoimmune diseases, inflammatory bowel diseases, ulcerative colitis, pneumonia, and ankylosing spondylitis [47]. Additionally, proteomics data analysis shows that TNFRSF1A is upregulated by drugs such as ascomycin, UNC0631, perhexiline, obatoclax, and berzosertib, and is phosphorylated by kinases including YANK2, MEK1, HPK1, and CDK6 [60, 61]. Similarly, IL1R1 is associated with RA and other inflammatory bowel diseases [47, 59]. IL6ST is linked to RA, multiple sclerosis, autoimmune diseases, inflammatory bowel diseases, pneumonia, and Crohn’s disease, and is regulated by multiple drugs and phosphorylated by CDK6 [47, 59]. From our analysis of 39 neighboring proteins of genetic risk factors in the cytokine signaling pathway, these findings suggest TNFRSF1A, IL1R1, and IL6ST are promising therapeutic targets and biomarkers for developing therapies and diagnostics for RA and related inflammatory diseases. This approach has the potential to improve treatment and disease management.

#### 3.4.2 Analysis of transcription factors.

Subnetwork analysis in Fig 7B identifies key transcription factors associated with various diseases [47, 59, 61]. Our results highlight RELA, ETS1, NFATC1, and ITGB2 as central players in the pathogenesis of various diseases, including RA, lymphoma, asthma, acute myelocytic leukemia, and leukemogenesis. The analysis demonstrates phosphorylation of these factors by kinases such as MST3, CHAK2, SLK, and LOK. GWAS data further strengthen the link between these genes and diseases, with BATF, CEBPA, and ETS1 associated with allergic diseases, and PTPN22, ETS1, PTPN11, and BATF linked to RA [61]. This convergence of disease association and kinase interaction suggests that RELA, ETS1, and NFATC1 are promising therapeutic targets and biomarkers, particularly for RA and related inflammatory diseases.

#### 3.4.3 Analysis of immune receptors and signaling.

The subnetwork analysis indicates that several kinases phosphorylate essential immune receptors and signaling proteins (Fig 7C). Specifically, NUAK1 phosphorylates SH2B3, PTPN22, LCK, GPR174, PTPN11, and RUNX1. Type 1 diabetes mellitus shares eight risk genes with RA. SH2B3 regulates JAK-STAT signaling and is a potential therapeutic target for RA [62, 63]. CD40 is a biomarker and therapeutic target for RA [47], while CRP is a common biomarker for multiple diseases. IRE2 is shown to phosphorylate FYN, IL2RA, PTPN22, and PTPN2. Additionally, MST3 phosphorylates IL2RA and PTPN22, while MST2 targets FYN and LCK. TNIK phosphorylates IL2RA and CYTIP. These interactions underscore the regulatory complexity and potential points of therapeutic intervention within the immune signaling pathways. Moreover, gene expression analysis reveals that the genes CD247, CYTIP, PTPRC, TRAF3IP3, CD96, IL2RB, PTPN22, PTPN2, FYN, and SLAMF6 are upregulated in CD56+ NK cells. Furthermore, TRAF3IP3, SLAMF6, CD3E, ITK, FYN, CD96, and LCK are upregulated in CD8+ T cells. This differential gene expression highlights the cell-type-specific roles of these genes in immune function and disease. Additionally, key genes such as IL2RA and CD247, linked with risk genes PTPN22, PTPN2, and RUNX1, are consistently implicated across multiple conditions, including RA, lupus erythematosus, B-cell lymphomas, and autoimmune diseases. These findings suggest that IL2RA, RUNX1, and associated kinases are potential targets for developing immune-related disease therapies and diagnostics.

### 3.5 Analysis of enzymes and kinases

Our analysis of neighboring proteins categorized as enzymes and kinases in Fig 7D highlights the complex interactions and regulatory mechanisms involving key proteins associated with various diseases. Several drugs modulate the expression of these proteins. For instance, betamethasone and formestane upregulate CDC25B, while toceranib upregulates FYN and downregulates CDC25B. GSK 650394 upregulates CDC25B and CCND3, whereas dasatinib downregulates CDC25B and upregulates RASGRP2. This modulation is crucial as gene expression data from the Human Gene Atlas reveals that proteins such as PIK3R1, CDC25B, CD3E, FYN, RASGRP2, and SH2D1A are upregulated in CD8+ T cells, and similar upregulation occurs in CD4+ T cells for CD3E, FYN, CDC25B, PIK3R1, ZAP70, and RASGRP2. Kinase interactions further illustrate that VRK1, CAMKK2, and TAK1 phosphorylate these proteins, highlighting their regulatory roles. These findings indicate that the identified genes CDC25B, FYN, PRKCB, PIK3R1, and CCND3, along with kinases VRK1, CAMKK2, and TAK1, are promising targets for therapeutic strategies and diagnostic tools for enzyme and kinase-related diseases.

### 3.6 Identification of potential therapeutic targets for RA

From our comprehensive analysis, we identified a total of 35 significant therapeutic targets and biomarkers for RA. These genes were categorized into three primary pathways: Pathway A (16 genes), Pathway B (17 genes), and Pathway C (2 genes). Among these, 25 genes were classified as risk genes, while 10 were neighboring genes, suggesting their functional relevance in RA pathogenesis. Notably, we identified nine novel proteins as potential therapeutic targets for RA based on their strong molecular interactions, involvement in immune signaling pathways, and inferred functional relevance in RA progression.

Based on our inference score, we selected the top genes with a score greater than 0.85 and considered them potential risk genes. We identified nine novel and candidate therapeutic targets for RA: PIK3R1, ETS1, NFATC1, LCK, PRKCB, BATF, RASGRP1, FYN, and RELA. These genes were prioritized based on their involvement in immune regulation, transcriptional control, and inflammatory signaling. (See Table 3).

**Table 3:** Potential therapeutic targets and biomarkers of RA.

Protein	Type	Inference Score	Situation	Novelty	PubMed ID (PMID)
RASGRP1	Risk Protein	0.93	Potential target	Novel	34199962, 34777472
NFATC1	Interaction Partner	0.93	Potential target	Novel	31972421, 34681582
RELA	Interaction Partner	0.92	Potential target	Novel	34750358, 37457726
ETS1	Interaction Partner	0.92	Potential target	Novel	38821936, 34199962
LCK	Interaction Partner	0.90	Potential target	Novel	36910149, 38473928
PIK3R1	Interaction Partner	0.90	Potential target	Novel	36992829
BATF	Risk Protein	0.90	Potential target	Novel	38821936
TNFRSF1A	Risk Protein	0.93	Identified target	Previously studied	37396624
IL1R1	Interaction Partner	0.92	Identified target	Previously studied	DrugBank
IL6ST	Interaction Partner	0.91	Identified target	Previously studied	35140805
CD40	Risk Protein	0.90	Identified biomarker	Previously studied	Biomarker Database
TGF-*β*1	Risk Protein	0.90	Identified target	Previously studied	25967237
PRKCB	Risk Protein	0.89	Potential target	Novel	33667569
RUNX1	Risk Protein	0.89	Potential target	Previously studied	36625319, 34737275
IRAK1	Risk Protein	0.89	Identified target	Previously studied	Biomarker
FYN	Interaction Partner	0.88	Potential target	Novel	33714238, 36740671, 36693907
SH2B3	Risk Protein	0.88	Identified target	Previously studied	32879140, 33067605
CRP	Risk Protein	0.88	Identified biomarker	Previously studied	34880861, 35379209
AIM2	Risk Protein	0.88	Identified target	Previously studied	37426634, 33976384
FLT3	Risk Protein	0.88	Potential target	Previously studied	33003568, 38238321
IL2RA	Interaction Partner	0.87	Potential target	Previously studied	31253980
IRF8	Risk Protein	0.87	Identified target	Previously studied	37066336, 33673123
PTPN2	Risk Protein	0.87	Identified biomarker	Previously studied	Biomarker
PTPN11	Risk Protein	0.86	Identified target	Previously studied	35265082
PTPN22	Risk Protein	0.86	Identified biomarker	Previously studied	35637605
AIRE	Risk Protein	0.85	Identified biomarker	Previously studied	Biomarker Database, 34199962, 32849810
MED1	Risk Protein	0.85	Identified target	Previously studied	31092410, 34777472
CDK2	Risk Protein	0.85	Potential target	Previously studied	38818077
PLD4	Risk Protein	0.84	Identified target	Previously studied	34199962, 32849810

### 3.7 Validation of novel RA therapeutic targets

**Fig 8:**
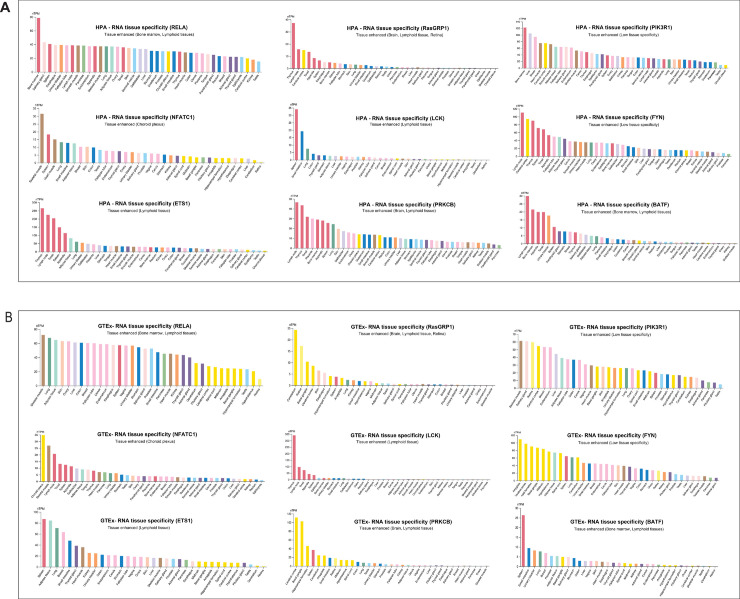
RNA tissue expression analysis of novel RA therapeutic targets. Gene expression profiles of the nine identified novel RA therapeutic targets across various tissues, highlighting their relevance in immune-related, hematopoietic, and neurological pathways. The analysis integrates GTEx and Human Protein Atlas (HPA) datasets, showing differential expression in bone marrow, lymphoid tissues, and the brain, which are critical for RA pathogenesis. These findings support the potential role of these genes in immune dysregulation and inflammation, reinforcing their viability as therapeutic targets.

The in silico validation of identified therapeutic targets was performed by analyzing their tissue-specific and single-cell expression patterns using data from GTEx, the HPA, and publicly available scRNA-seq datasets. As shown in Fig 8, our analysis of gene expression profiles supports the tissue relevance of the identified therapeutic targets. The result indicates that RELA, PIK3R1, and BATF are highly associated with bone marrow, highlighting their involvement in hematopoietic processes essential for immune function. Furthermore, RELA, RASGRP1, ETS1, PRKCB, BATF, and LCK are predominantly expressed in lymphoid tissues, reinforcing their role in immune cell activation and inflammatory pathways characteristic of RA. Additionally, RASGRP1, PRKCB, and NFATC1 show significant expression in the brain, suggesting potential neuroimmune interactions relevant to RA pathology.

**Fig 9:**
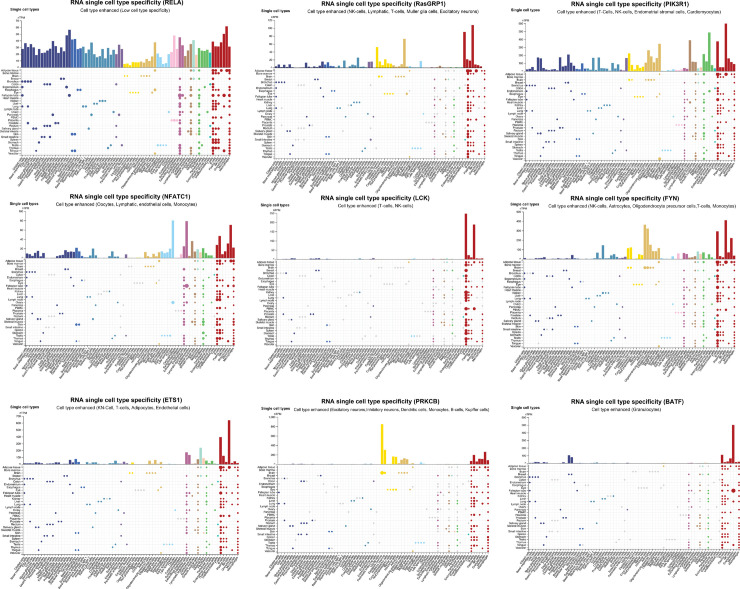
Single-cell RNA expression analysis of novel RA therapeutic targets. The scRNA-seq analysis of the nine identified novel RA therapeutic targets, highlighting their expression across different immune cell types. The results demonstrate strong enrichment in T cells, B cells, macrophages, and dendritic cells, reinforcing their involvement in immune regulation and RA pathogenesis. Notably, RASGRP1 and PRKCB exhibit elevated expression in brain cells, linking RA-associated genetic risk factors to neuroinflammatory pathways, while PIK3R1 shows significant expression in cardiomyocytes, suggesting potential cardiovascular implications.

Fig 9 presents the single-cell RNA expression analysis, which confirmed that all nine genes are highly expressed in immune cells and within the broader immune system, further strengthening their role as RA-related therapeutic targets. Notably, RASGRP1 and PRKCB exhibit elevated expression in brain cells, a finding that aligns with their identification as novel biomarkers for Alzheimer’s disease. This suggests a genetic link between RA and neurodegenerative disorders, potentially indicating shared inflammatory mechanisms. Moreover, PIK3R1 is highly expressed in cardiomyocytes, implicating a possible role in cardiovascular complications associated with RA.

### 3.8 Therapeutic implications and drug repurposing opportunities

Among the nine novel RA risk proteins, six are targetable by current or investigational drugs, highlighting opportunities for rapid repurposing with translational potential. For example, RELA is indirectly suppressed by dexamethasone, which binds NR3C1 and induces IκB, thereby blocking NF-κB nuclear translocation and inflammatory gene expression [64]. Celastrol, an investigational triterpene, directly inhibits RELA by impairing proteasome function and modulating heat shock proteins. However, further validation is required due to unresolved issues related to toxicity and formulation [65]. Tacrolimus and cyclosporine A inhibit calcineurin via immunophilin complexes, preventing NFATC1 dephosphorylation and nuclear entry, thus reducing T-cell activation and cytokine release. Dasatinib targets LCK and FYN, two Src kinases involved in T-cell receptor signaling. Although effective at the molecular level, its clinical application in RA is limited due to serious side effects such as bone marrow suppression and bleeding. Nonetheless, it may be useful for studying TCR-associated pathways. Alpelisib, a PI3K*α*-selective inhibitor, targets the PIK3CA–PIK3R1 complex and suppresses AKT/mTOR signaling, affecting lymphocyte survival and synovial fibroblast function [66]. Careful monitoring is necessary due to potential immune suppression. Enzastaurin inhibits PRKCB, thereby disrupting B-cell receptor signaling and angiogenesis processes linked to synovial inflammation [67]. RASGRP1 does not have direct inhibitors, but its downstream signaling can be modulated using MEK inhibitors such as trametinib and cobimetinib, which block ERK/MAPK activation. ETS1 and BATF currently lack approved inhibitors, though early-stage small molecules targeting ETS1 show preclinical promise [68]. BATF may be indirectly regulated through interventions targeting Th17 differentiation.Several of these drugs function indirectly by modulating upstream regulators or downstream effectors of RA-associated proteins. For instance, dexamethasone regulates RELA via NR3C1, while MEK inhibitors modulate the RASGRP1 pathway. Table 4 summarizes the associated therapeutic agents, molecular mechanisms, and clinical relevance. This list may serve as a library of drug repurposing candidates for RA, with validation in preclinical models anticipated, especially for high-risk or treatment-resistant cases.

**Table 4:** Therapeutic and pathway profiling of RA risk proteins via omics graphs.

Target	Drug(s)	Drug Class	Mechanism of Action	Pathway	Approved Indication(s)	Status	DrugBank ID(s)	Clinical Aspect
RELA	Dexamethasone, Celastrol	Glucocorticoid, Phytochemical	Dexamethasone activates NR3C1 to inhibit NF-κB; Celastrol directly inhibits NF-κB	NF-κB signaling, Cytokine production	Autoimmune diseases, Inflammation	Approved (Dexamethasone), Preclinical (Celastrol)	DB01234	Exploratory-phase Celastrol has undefined toxicity, precluding RA clinical use
NFATC1	Tacrolimus, Cyclosporine A	Calcineurin inhibitors	Inhibit calcineurin, preventing NFATC1 dephosphorylation and nuclear entry	Calcium–NFAT signaling, T-cell activation, Osteoclastogenesis	Transplant rejection, Autoimmune diseases	Approved	DB00864, DB00091	Effectively suppresses T-cell activity; requires careful management of immunosuppression in RA
LCK	Dasatinib	Src-family kinase inhibitor	Inhibits LCK (and FYN), blocking TCR signaling	T-cell receptor signaling, Immune synapse	Leukemia (CML, ALL)	Approved	DB01254	Marrow suppression and bleeding limit its use in RA, though it aids pathway analysis
FYN	Dasatinib	Src-family kinase inhibitor	Inhibits FYN (and LCK), suppressing T-cell receptor signaling	TCR signaling / Immune cell migration	Leukemia (CML, ALL)	Approved	DB01254	Safety concerns same as for LCK; severe side effects restrict RA use; relevant in mechanistic studies
PIK3R1	Alpelisib	PI3K*α* inhibitor	Inhibits PIK3CA affecting PIK3R1–PIK3CA heterodimer to suppress AKT/mTOR axis	PI3K–AKT–mTOR pathway, immune regulation	HR-positive, HER2-negative breast cancer	Approved	DB12015	Offers immune regulatory potential; caution warranted due to immunosuppression and infection risk in RA
PRKCB	Enzastaurin	PKC*β* inhibitor	Inhibits PRKCB, disrupting BCR signaling and angiogenesis	PKC*β* signaling, B-cell receptor axis, Angiogenesis	Glioblastoma, Lymphoma	Investigational	DB06486	Investigational status; requires additional validation for use in RA

These findings provide robust molecular evidence linking RA risk genes to critical immune, neurological, and cardiovascular functions. The convergence of these genes across multiple tissues and disease pathways underscores their therapeutic potential, not only in RA but also in related disorders such as Alzheimer’s disease and cardiovascular conditions. Integrating these insights enhances our understanding of RA pathophysiology and guides precision medicine strategies for identifying biomarkers and therapeutic targets across multifactorial diseases. While these agents show compelling mechanistic rationale, further validation in RA-specific preclinical models and early-phase clinical trials is essential. Future studies should assess efficacy, immunologic safety, and infection risk. Integrating multiomics-based risk profiles with clinical phenotypes may enable more personalized treatment strategies for RA.

## 4 Discussion

This study presents a comprehensive integrative analysis of genetic risk factors in RA using a multi-omics molecular network and knowledge graph approach. We developed a genetic risk factor–based knowledge graph integrating genomics, transcriptomics, and protein–protein interactions to elucidate RA molecular mechanisms. This approach enables the identification of key pathways, therapeutic targets, and drug repurposing opportunities based on functional connectivity, disease associations, and shared pathway signatures.

### 4.1 Key findings from the constructed multi-omics knowledge graph

Based on our knowledge graph analysis, we identified 35 risk proteins and their neighboring genes that share three main pathways in RA. Our analysis indicates that seven risk proteins were previously identified as biomarkers (CD40, CRP, PTPN2, PTPN22, AIRE, SLC8A3, and CDC25B) and therapeutic targets for RA, while 11 risk proteins (TNFRSF1A, IL1R1, IL6ST, TGF-*β*1, IRAK1, SH2B3, AIM2, IRF8, PTPN11, MED1, and PLD4) have been recognized for their involvement in disease progression. Among these 35 proteins, we identified nine novel proteins as potential therapeutic targets: RELA, ETS1, NFATC1, BATF, LCK, PIK3R1, PRKCB, RASGRP1, and FYN. Existing RA treatments target 11 previously identified as therapeutic target for RA, including TNFRSF1A, IL1R1, IL6ST, CD40, TGF-*β*1, IRAK1, SH2B3, AIM2, IL2RA, PTPN2, and FLT3, all of which are involved in cytokine signaling and immune regulation. For instance, RELA, BATF, and NFATC1 influence T helper cell differentiation and pro-inflammatory cytokine expression, including IL-17 [69]. ETS1 downregulation weakens Treg cell function, intensifying inflammatory pathways. PIK3R1, a regulatory subunit of phosphoinositide 3-kinase, modulates key immune pathways via AKT/mTOR signaling. RASGRP1, LCK, PRKCB, and FYN are integral to immune receptor signaling, including T-cell receptor activation and downstream kinase cascades. In addition to their role in RA, certain novel targets, such as RASGRP1 and PRKCB exhibit high expression in brain tissues, implicating them in neuroinflammatory processes and suggesting genetic overlap between RA and Alzheimer’s disease [70].

### 4.2 Functional characterization and therapeutic potential of RA targets

We further assessed the therapeutic relevance of these targets by integrating expression patterns with functional network analysis. The results confirmed a strong association between these proteins and RA pathogenesis, reinforcing their potential as therapeutic targets, particularly in drug-resistant cases. Although biologic therapies targeting TNF, IL-6, and IL-17 have significantly improved RA outcomes, a subset of patients remains unresponsive [71]. Our findings suggest that these newly identified proteins may contribute to drug resistance and offer alternative therapeutic strategies. RELA, a key regulator of the NF-κB pathway, may be persistently activated in TNF-inhibitor-resistant patients, making it a promising alternative target for non-responders [72]. The NF-κB pathway mediates pro-inflammatory signaling in both innate and adaptive immunity [72]. BATF, a transcription factor critical for Th17 differentiation and IL-17 production, has been implicated in IL-17-mediated inflammation. This suggests potential benefits from IL-17-targeted therapies in TNF-refractory RA cases [73, 74]. NFATC1, implicated in osteoclastogenesis, could serve as a therapeutic target for RA patients with bone erosion who do not respond to disease-modifying antirheumatic drugs [69]. Additionally, PIK3R1 is highly expressed in immune-related tissues and exhibits strong network connectivity with known RA-associated proteins, reinforcing its therapeutic potential [75]. Furthermore, PIK3R1, which plays a role in kinase signaling, was highly expressed in cardiomyocytes, suggesting a connection between RA and cardiovascular disease. Notably, its expression in cardiomyocytes suggests a mechanistic link between RA and cardiovascular disease, aligning with the observed increase in atherosclerosis among RA patients [76]. Additionally, tissue-specific and single-cell analyses showed high expression of the identified genes in synovial tissue, immune cells, and lymphoid organs [77]. RASGRP1 and PRKCB displayed elevated expression in brain tissue, suggesting a genetic connection between RA and neuroinflammatory diseases such as Alzheimer’s disease [77, 78].

### 4.3 Implications for drug pepurposing and disease progression

Our findings highlight the potential of multi-omics knowledge graph analysis in elucidating the genetic risk factors contributing to RA pathogenesis [79, 80]. These graphs integrate biological priors with omics data to uncover functional networks, enabling cross-disease analysis [80, 81]. The overlap in risk pathways among RA, diabetes, AD, and cardiovascular diseases supports the hypothesis of shared inflammatory and immune-related etiologies [82].This approach offers a promising framework for understanding the shared pathogenic mechanisms among multiple diseases, including diabetes, Alzheimer’s disease, and cardiovascular disorders. Specifically, RASGRP1, PRKCB, and NFATC1 are implicated in both RA and Alzheimer’s disease, suggesting therapeutic convergence. Dysregulation of these genes contributes to systemic inflammation and immune dysregulation, features common to RA and neurodegenerative disorders [78, 83]. PRKCB has been explored as a therapeutic target in AD via protein kinase C modulation [77, 84]. The observed genetic overlap provides opportunities for drug repurposing. Existing therapies for chronic diseases, such as cancer or AD, may be repositioned for RA based on shared gene expression and protein interaction networks [85]. Bioinformatics-based drug repurposing efforts have identified several such candidates [85]. These results support the emerging model of precision drug repurposing—leveraging genetic and molecular data to tailor treatments to individual patients [85–87]. Integrating omics data with clinical profiles allows for the development of predictive models that inform prognosis, treatment response, and long-term risk stratification in RA [87].

### 4.4 Potential medical applications and precision medicine approaches

The application of precision medicine in RA remains limited by the absence of predictive biomarkers [8, 87]. Our comprehensive approach underscores the potential for targeting specific organs, tissues, and cell types to enhance RA treatment efficacy, slow disease progression, and mitigate complications [81, 87]. Additionally, this study establishes a robust framework for utilizing genetic risk network analysis to identify critical pathways, potential therapeutic targets, and repurposing opportunities [85, 86]. By integrating genetic risk factor analyses, this research uncovers previously overlooked therapeutic targets and disease mechanisms, offering new insights for future RA treatment development [85, 88]. Genetic risk scores linked to over 30 RA-associated loci explain approximately half of the disease’s heritability [70, 88]. Integrating these risk loci with clinical, genomic, and environmental data enables risk stratification and early prediction of complications [88]. Our findings suggest that existing drugs approved for diseases that share common genetic factors and pathways with RA may be repurposed for RA treatment. Integrating genetic risk factors with genomic and biomedical data is crucial for anticipating future complications in RA. Furthermore, this study sheds new light on the molecular mechanisms underlying RA by integrating multi-omics data, genetic risk factors, and molecular network analyses [79, 84]. Understanding common pathways linking therapeutic targets, biomarkers, and organ dysfunction will enable more precise treatment planning and decision-making [24, 81]. The enrichment network analysis presented here may contribute to the identification of novel biomarkers, prioritization of organ-specific treatments, and discovery of potential therapeutic targets for RA [79, 80]. Graph models that integrate genetic and clinical data will be essential for shaping personalized, proactive, and preventive strategies in RA care. [87, 89]

### 4.5 Limitations and challenges

This study contributes to the medical analysis of RA by integrating multi-omics knowledge graph analysis of genetic risk factors, which may support personalized treatment strategies and predict potential complications [87]. Despite its promising implications, several challenges must be addressed before clinical application. One limitation is that the multi-omics analysis was conducted using a specific dataset, requiring validation across independent cohorts and technological platforms to confirm reproducibility [79]. Additionally, a larger sample size and stratified analyses based on disease progression and treatment response are needed to account for RA patient heterogeneity [90]. Experimental validation through in vivo and in vitro studies is essential to confirm the roles of these genes in RA pathogenesis and their potential as therapeutic targets. Understanding the interactions between genetic factors and environmental influences may further explain the complex etiology of RA [90]. The multifactorial nature of RA, involving the interplay between genetic and environmental factors, complicates treatment strategies [78, 85, 90]. While these findings provide a foundation for further research, functional studies are needed to determine the mechanistic roles of these genes in RA pathogenesis and treatment response. Future research will incorporate epigenetic and environmental data, diagnostic radiology, and pharmacological treatment information to refine precision medicine approaches, with the goal of developing individualized treatment strategies and preventing chronic RA [86, 89].

## 5 Conclusion

Our study has employed an integrated approach to analyze genetic risk factors and protein interactions in RA, identifying several key pathways and potential therapeutic targets. The identified pathways—Cytokine Regulation and Production, Hematopoietic or Lymphoid Organ Development, and Myeloid Cell Differentiation—are crucial in immune cell development and inflammatory responses. The study highlights seven risk genes intersecting all three pathways, presenting promising targets for therapeutic intervention. Furthermore, we identified 35 core region genes, including both recognized and new potential therapeutic targets, involved in immune responses, cell signaling, and inflammation. This comprehensive analysis underscores the importance of integrating multi-omics enrichment with network analysis to uncover the molecular mechanisms underlying RA. These findings provide a strong foundation for future research and the development of targeted therapies, with the potential to significantly improve RA management and patient outcomes. Further experimental validation and exploration of genetic and environmental interactions are necessary to advance our understanding and treatment of RA.

## Supporting information

S1 Table Genetic Risk Variants of RA: List of retrieved genetic risk SNP of Rheumatoid Arthritis retrieved from biomedical articles. (PDF)

S2 Table Annotated Genetic Risk Variants of RA: List of annotated genetic risk variants of Rheumatoid Arthritis retrieved from biomedical articles. (PDF)

S3 Table Annotated Genetic Risk Genes: List of annotated genetic risk genes of Rheumatoid Arthritis retrieved from biomedical articles. (PDF)

S4 Table Molecular network: Detailed information on the molecular functions and biological processes associated with the constructed networks of RA genetic risk factors. (PDF)

S5 Table Therapeutic targets and biomarker validation dataset of RA: List of approved therapeutic targets and biomarkers of RA from DrugBank and biomarker databases. (PDF)
