# Supplementary material for: Integrative molecular network analysis of genetic risk factors to infer biomarkers and therapeutic targets for rheumatoid arthritis
Source: PLoS One. 2025 Aug 21;20(8):e0329101. doi: 10.1371/journal.pone.0329101 (PMC12370121; doi:10.1371/journal.pone.0329101)
Supplement: S1 Table — (PDF) [file pone.0329101.s001.pdf]

Risk variants	Chromosome	Risk gene	OR	95%CI	PMID ID ref.		
rs998731	8	TPD52	1.08	(1.05–1.11)	35088123, 23143596		
rs9979383	21	RUNX1	1.08	(1.06–1.11)	23143596, 35088123, 33310728, 23143596		
rs9943599	11	SWAP70	1.09	(1.06–1.11)	33310728, 32723749		
rs9927316	16	IRF8	0.91	(0.89–0.94)	PMID32232558 , 23143596, 35088123, 24390342, 33310728		
rs9826828	3	IL20RB	1.44	(1.28–1.61)	24390342		
rs968567	11	FADS2	1.12	(1.07–1.16)	23143596, 35088123, 24390342		
rs9603616	13	COG6	0.89	(0.87–0.91)	33310728, 33310728		
rs9557321	13	CLYBL	1.73	(1.42–2.11)	24532677		
rs9532434	13	COG6	0.89	(0.87–0.91)	33310728, 33310728		
rs950918814	4	ANTXR2	0.93	(0.91–0.95)	35088123		
rs947474	10	PRKCQ	0.92	(0.90–0.94)	23143596, 22446963		
rs940825	7	AGR3-AHR	1.13	(1.08–1.18)	35088123		
rs9378815	6	IRF4	0.91	(0.89–0.94)	35088123, 23143596		
rs9373594	6	PPIL4	1.09	(1.06–1.12)	24390342		
rs9372120	6	PRDM1-ATG5	1.11	(1.07–1.15)	24390342, 35088123, 19898481		
rs934734	2	SPRED2	0.9	(0.88–0.93)	22446963, 20453842		
rs932036	4	RBPJ	1.15	(1.11–1.19)	24390342, 23143596, 20453842		
rs911760	9	PLGRKT	1.15	(1.09–1.20)	35088123		
rs909685	22	SYNGR1	1.14	(1.11–1.17)	23143596, 33310728		
rs9005	2	IL1RN	N.A	N.A	PMID31852669		
rs883220	1	POU3F1	0.89	(0.86–0.92)	35088123, 23143596		
rs874040	4	RBPJ	1.15	(1.11–1.19)	24390342, 23143596, 20453842		
rs866205108	18	TNFRSF11A	1.1	(1.06–1.14)	35088123		
rs8133843	21	RUNX1	1.08	(1.06–1.11)	23143596, 35088123, 33310728, 23143596		
rs8126756	21	IFNGR2	1.09	(1.06–1.12)	35088123, 33310728, 23143596		
rs8106598	19	SIGLEC6	1.08	(1.05–1.11)	35088123		
rs8083786	18	PTPN2	0.91	(0.89–0.94)	22446963, 23143596, 35088123		
rs8073171	17	C1QBP	1.12	(1.08–1.17)	35088123, 23143596		
rs8043085	15	RASGRP1	0.88	(0.86–0.90)	23143596, 35088123, 24390342, 33310728		
rs8032939	15	RASGRP1	0.88	(0.86–0.90)	23143596, 35088123, 24390342, 33310728		
rs8026898	15	TLE3	1.14	(1.11–1.17)	23143596, 35088123		

Risk variants	Chromosome	Risk gene	OR	95%CI	PMID ID ref.		
rs798000	1	CD2	1.12	(1.09–1.16)	35088123, 22446963		
rs7943728	11	FADS3	1.12	(1.07–1.16)	23143596, 35088123, 24390342		
rs793108	10	ZNF438	1.07	(1.05–1.09)	33310728, 23143596, 35088123		
rs793095	10	ZNF438	1.07	(1.05–1.09)	33310728, 23143596, 35088123		
rs79145843	11	PDE2A-ARAP1	0.88	(0.84–0.91)	33310728, 35088123, 22446963		
rs7902146	10	ARID5B	1.16	(1.13–1.19)	23143596		
rs7848647	9	TL1A	N.A	N.A	PMID30488533		
rs77574423	3	TAMM41-SYN2	0.89	(0.86–0.93)	35088123		
rs7752903	6	TNFAIP3	1.33	(1.26–1.40)	23143596, 33310728, 22446963, 17982455		
rs77465633	12	SH2B3-PTPN11	1.32	(1.20–1.45)	33310728, 33310728		
rs7731626	5	ANKRD55	0.82	(0.79–0.85)	33310728, 35088123, 23143596, 20453842		
rs773125	12	CDK2	1.09	(1.07–1.12)	24390342, 33310728		
rs77191406	6	A20	N.A	N.A	PMID27435953		
rs762574969	1	GNG4	0.91	(0.88–0.94)	35088123		
rs7574865	2	STAT4	N.A	N.A	PMID27342690		
rs7574865	2	STAT4	1.16	(1.13–1.19)	22446963, 23143596		
rs7540342	1	POU3F1	0.89	(0.86–0.92)	35088123, 23143596		
rs74842123	X	GPR174-KIF4CF	1.11	(1.08–1.15)	23143596, 35088123, 33310728		
rs740122	7	JAZF1	0.92	(0.90–0.95)	24390342, 33310728		
rs734094	11	TSPAN32	1.08	(1.05–1.10)	35088123		
rs73366469	7	GTF2IRD1-NCF1	1.43	(1.33–1.55)	24390342		
rs7324510	13	FLT-1	N.A	N.A	PMID28323906		
rs73194058	21	IFNGR2	1.09	(1.06–1.12)	35088123, 33310728, 23143596		
rs73081554	3	DNASE1L3	1.18	(1.11–1.25)	24390342		
rs73013527	11	FLI1-ETS1	0.92	(0.90–0.95)	24390342, 35088123		
rs73005423	11	DDX6	1.15	(1.09–1.22)	35088123, 23143596, 22446963		
rs72928038	6	BACH2	1.09	(1.06–1.11)	PMID28711138		
rs7278257	21	AIRE	0.91	(0.89–0.94)	21505073, 23143596, 35088123, 33310728		
rs72634030	17	C1QBP	1.12	(1.08–1.17)	35088123, 23143596		
rs726288	10	SFTPD	1.14	(1.07–1.20)	22446963		

Risk variants	Chromosome	Risk gene	OR	95%CI	PMID ID ref.		
rs7241016	18	PTPN2	0.91	(0.89–0.94)	22446963, 23143596, 35088123		
rs7206670	16	TXNDC11	1.07	(1.05–1.10)	33310728, 23143596		
rs7171617	15	IQGAP1	1.12	(1.08–1.16)	33310728, 35088123		
rs71624119	5	ANKRD55	0.85	(0.80–0.91)	35088123, 23143596		
rs7097397	10	WDFY4	0.92	(0.90–0.94)	23143596, 22446963,35088123		
rs706778	10	IL2RA	1.1	(1.08–1.13)	33310728, 23143596, 20453842		
rs705700	12	CDK2	1.09	(1.07–1.12)	24390342, 33310728		
rs6979218	7	CASTOR3-SPDY	0.92	(0.89–0.94)	35088123		
rs6932056	6	TNFAIP3	1.33	(1.26–1.40)	23143596, 33310728, 22446963, 17982455		
rs6930468	6	IRF4	0.91	(0.89–0.94)	35088123, 23143596		
rs6920220	6	TNFAIP3	1.33	(1.26–1.40)	23143596, 33310728, 22446963, 17982455		
rs6859212	5	ANKRD55	0.82	(0.79–0.85)	33310728, 35088123, 23143596, 20453842		
rs6814280	4	KIAA1109	0.93	(0.90–0.96)	35088123		
rs678347	8	GRHL2	1.08	(1.05–1.11)	35088123, 23143596		
rs6732565	2	ACOXL	1.07	(1.05–1.10)	24390342, 35088123		
rs67318457	6	NRSN1	1.09	(1.05–1.12)	35088123		
rs67250450	7	JAZF1	0.92	(0.90–0.95)	24390342, 33310728		
rs6715284	2	CASP8	1.15	(1.10–1.20)	24390342, 35088123		
rs6705628	2	DGUOK-AS1	0.88	(0.85–0.92)	22446963		
rs66922517	21	RUNX1	1.08	(1.06–1.11)	23143596, 35088123, 33310728, 23143596, 36859360		
rs6681482	1	TNFSF4	1.12	(1.08–1.15)	24532676, 35088123, 24390342		
rs6619397	X	GPR174-KIF4CF	1.11	(1.08–1.15)	23143596, 35088123, 33310728		
rs660442	11	BAD	0.9	(0.87–0.93)	33310728, 35088123		
rs6583441	7	IKZF1	0.95	(0.93–0.97)	35088123		
rs657075	5	CSF2	1.09	(1.06–1.12)	35088123, 23143596		
rs6546146	2	SPRED2	0.9	(0.88–0.93)	22446963, 20453842		
rs6495979	15	RASGRP1	0.88	(0.86–0.90)	23143596, 35088123, 24390342, 33310728		
rs6479800	10	RTKN2	1.11	(1.07–1.15)	23028356, 23143596		
rs6478109	9	TL1A	N.A	N.A	PMID30488533		
rs629326	6	TAGAP	1.11	(1.08–1.14)	24390342, 23143596		

Risk variants	Chromosome	Risk gene	OR	95%CI	PMID ID ref.
rs624988	1	CD2	1.12	(1.09–1.16)	35088123, 22446963
rs62264113	3	TPRA1	0.92	(0.89–0.95)	35088123
rs61944750	13	FLT3	0.91	(0.88–0.94)	35088123
rs61828284	1	TNFSF4	1.12	(1.08–1.15)	24532676, 35088123, 24390342
rs6032662	20	CD40	0.9	(0.88–0.92)	19898481,35088123, 22446963, 23143596
rs6011186	20	C20orf181	0.9	(0.87–0.93)	33310728, 35088123
rs5987194	X	IRAK1	1.15	(1.12–1.18)	24390342, 33310728, 23143596
rs59578717	11	TPCN2	0.91	(0.88–0.94)	33310728
rs595158	11	FADS1	1.12	(1.07–1.16)	23143596, 35088123, 24390342
rs59466457	6	CCR6	0.86	(0.84–0.88)	23143596, 22446963, 20453842
rs591549	18	DLGAP1	0.91	(0.88–0.94)	35088123
rs58107865	4	LEF1	0.84	(0.80–0.88)	35088123
rs5756407	22	CSF2RB-LOC10	1.06	(1.04–1.08)	35088123
rs5754104	22	UBE2L3-YDJC	1.09	(1.06–1.12)	23143596, 35088123, 33310728
rs56787183	5	PTGER4	0.85	(0.80–0.90)	35088123
rs56750287	17	GSDMB	0.93	(0.91–0.95)	23143596
rs55762233	19	HAPLN4	1.1	(1.07–1.14)	35088123
rs548234	6	PRDM1-ATG5	1.11	(1.07–1.15)	24390342, 35088123, 19898481
rs502919	10	PRKCQ	0.92	(0.90–0.94)	23143596, 22446963
rs4963581	12	LOC105369698	1.09	(1.06–1.12)	33310728
rs4938573	11	DDX6	1.15	(1.09–1.22)	35088123, 23143596, 22446963
rs4840565	8	BLK	1.12	(1.09–1.15)	19503088, 35088123, 33310728, 23143596
rs4810485	20	CD40	0.9	(0.88–0.92)	19898481,35088123, 22446963, 23143596
rs4809371	20	C20orf181	0.9	(0.87–0.93)	33310728, 35088123
rs479777	11	BAD	0.9	(0.87–0.93)	33310728, 35088123
rs4780401	16	TXNDC11	1.07	(1.05–1.10)	33310728, 23143596
rs4690029	4	FAM193A	0.94	(0.92–0.96)	35088123
rs4687070	3	TPRG1-TP63	1.15	(1.09–1.20)	35088123
rs4655698	1	IL12RB2	1.09	(1.05–1.11)	35088123
rs4622308	12	CDK2	1.09	(1.07–1.12)	24390342, 33310728

Risk variants	Chromosome	Risk gene	OR	95%CI	PMID ID ref.		
rs4602367	3	PLCL2	0.93	(0.91–0.95)	33310728, 24390342		
rs4584833	16	TXNDC11	1.07	(1.05–1.10)	33310728, 23143596		
rs4452313	3	PLCL2	0.93	(0.91–0.95)	33310728, 24390342		
rs4409785	11	CEP57	0.91	(0.88–0.94)	24390342, 33310728		
rs4272	7	CDK6	0.92	(0.89–0.94)	35088123, 23143596		
rs42044	7	CDK6	0.92	(0.89–0.94)	35088123, 23143596		
rs419598	12	IL1RN	N.A	N.A	PMID31852669		
rs41313373	1	GFI1	1.12	(1.08–1.16)	35088123		
rs41269479	1	HIVEP3	1.15	(1.09–1.20)	35088123		
rs403214	5	MACIR	1.1	(1.06–1.13)	35088123, 23143596,20453842,3310728,		
rs3890745	1	MMEL1	0.91	(0.89–0.93)	23143596, 19898481, 22446963		
rs3825568	14	ZFP36L1	1.08	(1.06–1.11)	32723749, 33310728		
rs3824660	10	GATA3	0.93	(0.91–0.95)	23143596, 23143596, 35088123		
rs3810936	9	TL1A	N.A	N.A	PMID30488533		
rs3807306	7	IRF5	0.88	(0.86–0.91)	23143596, 22446963, 20453842		
rs3806624	3	EOMES	0.92	(0.89–0.94)	33310728, 24390342		
rs3804333	6	PRDM1-ATG5	1.11	(1.07–1.15)	24390342, 35088123, 19898481		
rs3783782	14	PRKCH	1.14	(1.09–1.18)	33310728, 33310728		
rs3781913	11	PDE2A-ARAP1	0.88	(0.84–0.91)	33310728, 35088123, 22446963		
rs3764880	X	TLR8	N.A	N.A	PMID36262248		
rs3764879	X	TLR8	N.A	N.A	PMID36262248		
rs3761847	9	TRAF1-C5	0.92	(0.90–0.95)	17804836, 22446963		
rs3753389	1	CD244	1.3	(1.18–1.43)	18794858		
rs35156883	22	SMC1B	1.1	(1.06–1.13)	33310728		
rs34695944	2	REL	1.12	(1.08–1.15)	24390342, 19503088		
rs34536443	19	TYK2	0.68	(0.62–0.75)	23143596		
rs34480360	16	ZNF689	1.09	(1.06–1.12)	33310728, 35088123		
rs3184504	12	SH2B3-PTPN11	1.32	(1.20–1.45)	33310728, 33310728		
rs315952	2	IL1RN	N.A	N.A	PMID31852669		
rs315952	2	IL1RN	N.A	N.A	PMID31852669		

Risk variants	Chromosome	Risk gene	OR	95%CI	PMID ID ref.		
rs3134883	10	IL2RA	1.1	(1.08–1.13)	33310728, 23143596, 20453842		
rs3125734	10	RTKN2	1.11	(1.07–1.15)	23028356, 23143596		
rs3093023	6	CCR6	0.86	(0.84–0.88)	23143596, 22446963, 20453842		
rs3087243	2	CTLA4	0.88	(0.86–0.90)	20453842, 35088123		
rs3001423	14	PLD4	0.85	(0.82–0.88)	22446963, 35088123, 23143596		
rs2918392	5	DAP	0.94	(0.91–0.96)	33310728, 35088123		
rs2910164	5	MIR146A	N.A	N.A	PMID27342690		
rs2872507	17	GSDMB	0.93	(0.91–0.95)	23143596, 35088123, 24390342		
rs2867461	4	ANXA3	1.13	(1.09–1.17)	22446963		
rs2847297	18	PTPN2	0.91	(0.89–0.94)	22446963, 23143596, 35088123		
rs2843401	1	MMEL1	0.91	(0.89–0.93)	23143596, 19898481, 22446963		
rs2841277	14	PLD4	0.85	(0.82–0.88)	22446963, 35088123, 23143596		
rs28411352	1	MTF1	1.1	(1.07–1.13)	24390342		
rs28398409	1	PTPRC	0.91	(0.88–0.94)	35088123		
rs28373672	19	KMT2B	0.93	(0.91–0.96)	35088123		
rs28362855	6	NFKBE	1.22	(1.17–1.26)	35088123, 33310728		
rs2833522	21	ACPA	N.A	N.A	PMID26077402, PMID33609792		
rs2812378	9	CCL21	1.12	(1.09–1.16)	35088123, 23143596, 22446963, 20453842		
rs2736340	8	BLK	1.12	(1.09–1.15)	19503088, 35088123, 33310728, 23143596		
rs2671692	10	WDFY4	0.92	(0.90–0.94)	23143596, 22446963,35088123		
rs2664035	4	TEC	1.07	(1.04–1.10)	24390342		
rs2582532	14	PLD4	0.85	(0.82–0.88)	22446963, 35088123, 23143596		
rs2561477	5	MACIR	1.1	(1.06–1.13)	35088123, 23143596,20453842,3310728,		
rs2542151	18	PTPN22	N.A	N.A	PMID27342690 , PMID32678001		
rs2476601	1	PTPN22	1.81	(1.73–1.89)	15208781, 20453842		
rs2469434	18	CD226	1.07	(1.05–1.10)	23143596, 35088123		
rs2451258	6	TAGAP	1.11	(1.08–1.14)	24390342, 23143596		
rs244685	5	CSF2	1.09	(1.06–1.12)	35088123, 23143596		
rs244468	5	ARHGAP26	0.93	(0.91–0.95)	35088123		
rs2317231	1	FCRL3	1.08	(1.05–1.10)	24390342		

Risk variants	Chromosome	Risk gene	OR	95%CI	PMID ID ref.		
rs2317230	1	FCRL3	1.08	(1.05–1.10)	24390342		
rs2305480	17	GSDMB	0.93	(0.91–0.95)	23143596, 35088123, 24390342		
rs2301888	1	PADI4	0.88	(0.86–0.90)	35088123, 23143596, 22446963, PMID36262248		
rs2300373	21	IFNGR2	1.09	(1.06–1.12)	35088123, 33310728, 23143596		
rs2275806	10	GATA3	0.93	(0.91–0.95)	23143596, 23143596, 35088123		
rs227163	1	TNFRSF9	1.04	(1.02–1.06)	24390342		
rs2258734	1	MMEL1	0.91	(0.89–0.93)	23143596, 19898481, 22446963		
rs2240336	1	PADI4	0.88	(0.86–0.90)	35088123, 23143596, 22446963		
rs2234067	6	ETV7	1.15	(1.10–1.20)	24390342, 35088123		
rs2233945	6	PSORS1C1	N.A	N.A	PMID27342690		
rs2233424	6	POLR1C	1.22	(1.17–1.26)	35088123, 33310728		
rs2228145	1	IL6R	0.93	(0.91–0.95)	33310728, 24390342, 23143596		
rs2147161	13	AKAP11- LINC02	1.1	(1.06–1.13)	35088123		
rs2141331	2	CASP8	1.15	(1.10–1.20)	24390342, 35088123		
rs2105325	1	TNFSF4	1.12	(1.08–1.15)	24532676, 35088123, 24390342		
rs2075876	21	AIRE	0.91	(0.89–0.94)	21505073, 23143596, 35088123, 33310728		
rs2069235	22	SYNGR1	1.14	(1.11–1.17)	23143596, 33310728		
rs201408742	X	GPR174-KIF4CF	1.11	(1.08–1.15)	23143596, 35088123, 33310728		
rs199894206	15	IQGAP1	1.12	(1.08–1.16)	33310728, 35088123		
rs1980422	2	CD28	1.12	(1.09–1.16)	19898481		
rs1950897	14	RAD51B	1.11	(1.08–1.14)	24390342, 33310728		
rs1943199	18	LINC01898	1.94	(1.54–2.44)	24532677		
rs1893592	21	UBASH3A	1.1	(1.07–1.13)	23143596		
rs1885013	14	RAD51B	1.11	(1.08–1.14)	24390342, 33310728		
rs1883832	20	CD40	0.9	(0.88–0.92)	19898481,35088123, 22446963, 23143596		
rs1877030	17	MED1	1.09	(1.06–1.12)	23143596		
rs187579	5	MACIR	1.1	(1.06–1.13)	35088123, 23143596,20453842,3310728,		
rs1858037	2	SPRED2	0.9	(0.88–0.93)	22446963, 20453842		
rs182199544	7	SKAP2-HOXA1	0.87	(0.84–0.91)	35088123		
rs1800896	1	IL10	N.A	N.A	PMID36233253		

Risk variants	Chromosome	Risk gene	OR	95%CI	PMID ID ref.		
rs1800872	1	IL10	N.A	N.A	PMID27342690		
rs1800797	7	IL6	N.A	N.A	PMID33074413		
rs1800796	7	IL6	N.A	N.A	PMID33074413		
rs1800470	19	TGFB1	N.A	N.A	PMID34046752,PMID36233253		
rs1800469	19	TGFB1	N.A	N.A	PMID34046752 , PMID36233253		
rs175714	14	BATF	0.94	(0.92–0.96)	35088123		
rs1696466	12	OS9-AGAP2	1.06	(1.04–1.08)	23143596, 35088123		
rs16903108	8	PVT1	1.15	(1.10–1.20)	35088123, 23143596		
rs1571878	6	CCR6	0.86	(0.84–0.88)	23143596, 22446963, 20453842		
rs1538981	10	ZNF438	1.07	(1.05–1.09)	33310728, 23143596, 35088123		
rs1516971	8	PVT1	1.15	(1.10–1.20)	35088123, 23143596		
rs149041927	16	PRKCB	0.91	(0.88–0.94)	23143596, 35088123, 24390342, 33310728		
rs147622113	19	ILF3	0.68	(0.60–0.77)	22446963		
rs146492555	14	PRKCH	1.14	(1.09–1.18)	33310728, 33310728		
rs143259280	2	PCBP1-AS1	1.09	(1.06–1.12)	35088123		
rs143107126	18	CD226	1.07	(1.05–1.10)	23143596, 35088123		
rs1427749	12	SCAF11	1.08	(1.05–1.11)	35088123		
rs1422673	5	TNIP1	1.1	(1.06–1.14)	35088123		
rs138193887	11	CUL5	1.21	(1.13–1.29)	33310728, 35088123, 22446963		
rs13426947	2	STAT4	1.16	(1.13–1.19)	22446963, 23143596		
rs13397	X	IRAK1	1.15	(1.12–1.18)	24390342, 33310728, 23143596		
rs13330176	16	IRF8	0.91	(0.89–0.94)	23143596, 35088123, 24390342, 33310728		
rs13142500	4	CLNK	1.1	(1.08–1.13)	24390342, 33310728		
rs13103285	4	CLNK	1.1	(1.08–1.13)	24390342, 33310728		
rs13031237	2	REL	1.12	(1.08–1.15)	24390342, 19503088		
rs12918327	16	ZNF689	1.09	(1.06–1.12)	33310728, 35088123		
rs12795702	11	LOC107984408	1.09	(1.06–1.12)	33310728		
rs12764378	10	ARID5B	1.16	(1.13–1.19)	23143596, 22446963,35088123		
rs12712065	2	AFF3	1.1	(1.08–1.13)	23143596, 35088123, 20453842		
rs1264600	8	GRHL2	1.08	(1.05–1.11)	35088123, 23143596		

Risk variants	Chromosome	Risk gene	OR	95%CI	PMID ID ref.		
rs12530098	6	CD83	1.15	(1.10–1.20)	35088123, 23143596		
rs12529514	6	CD83	1.15	(1.10–1.20)	35088123, 23143596		
rs12212067	6	FOXO3	N.A	N.A	PMID27214848		
rs12145329	1	PTGS2	0.92	(0.89–0.95)	35088123, 22446963		
rs12126142	1	IL6	0.93	(0.91–0.95)	33310728, 24390342, 23143596, PMID36574943		
rs12026490	1	SLAMF6	0.8	(0.75–0.85)	33310728		
rs11933540	4	RBPJ	1.15	(1.11–1.19)	24390342, 23143596, 20453842		
rs11900673	2	B3GNT2	1.15	(1.08–1.21)	22446963		
rs11777380	8	CCN4	0.92	(0.90–0.95)	35088123		
rs11586238	1	CD2	1.12	(1.09–1.16)	35088123, 22446963		
rs11574914	9	CCL21	1.12	(1.09–1.16)	35088123, 23143596, 22446963, 20453842		
rs11571302	2	CTLA4	0.88	(0.86–0.90)	20453842, 35088123		
rs115284761	15	PSTPIP1	0.91	(0.89–0.94)	35088123		
rs11454989	21	AIRE	0.91	(0.89–0.94)	21505073, 23143596, 35088123, 33310728		
rs1143634	2	IL1beta	N.A	N.A	PMID33074413		
rs1143627	2	IL1beta	N.A	N.A	PMID33074413		
rs11420145	6	ETV7	1.15	(1.10–1.20)	24390342, 35088123		
rs11375064	17	KSR1	0.93	(0.90–0.95)	23143596, 35088123, 24390342		
rs113532504	6	JARID2	1.13	(1.08–1.18)	35088123		
rs113066392	7	GTF2IRD1-NCF1	1.43	(1.33–1.55)	33310728, 28135245, 27272985		
rs11089637	22	UBE2L3-YDJC	1.09	(1.06–1.12)	23143596, 35088123, 33310728		
rs10985070	9	TRAF1-C5	0.92	(0.90–0.95)	17804836, 22446963		
rs10917571	1	FCGR3A	0.91	(0.89–0.94)	35088123		
rs10911902	1	PTGS2	0.92	(0.89–0.95)	35088123, 22446963		
rs10905284	10	GATA3	0.93	(0.91–0.95)	23143596, 23143596, 35088123		
rs10892279	11	DDX6	1.15	(1.09–1.22)	35088123, 23143596, 22446963		
rs10821944	10	ARID5B	1.16	(1.13–1.19)	23143596, 22446963,35088123		
rs10790268	11	CXCR5	0.87	(0.84–0.90)	24390342		
rs10774624	12	SH2B3-PTPN11	1.32	(1.20–1.45)	33310728, 33310728		
rs10683701	12	OS9-AGAP2	1.06	(1.04–1.08)	23143596, 35088123		

Risk variants	Chromosome	Risk gene	OR	95%CI	PMID ID ref.		
rs10556591	11	FLI1-ETS1	0.92	(0.90–0.95)	24390342, 35088123		
rs10499194	6	TNFAIP3	1.33	(1.26–1.40)	23143596, 33310728, 22446963, 17982455		
rs10497813	2	PLCL1-LINC019[illegible]	1.06	(1.04–1.09)	30891314, 35088123		
rs10488631	7	IRF5	0.88	(0.86–0.91)	23143596, 22446963, 20453842		
rs10453119	8	TPD52	1.08	(1.05–1.11)	35088123, 23143596		
rs1044165	X	VSIG4	N.A	N.A	PMID33645007		
rs10415976	19	ARID3A	0.92	(0.90–0.95)	35088123		
rs10209110	2	AFF3	1.1	(1.08–1.13)	23143596, 35088123, 20453842		
rs10175798	2	LBH	0.92	(0.90–0.94)	24390342		
N.A	N.A	MIC-1	N.A	N.A	PMID17328047		
N.A	N.A	AIM2	N.A	N.A	PMID36476420		
N.A	N.A	CDK5RAP2	N.A	N.A	PMID28711138		
N.A	N.A	CRP	N.A	N.A	PMID36233253		
N.A	N.A	DPP4	N.A	N.A	PMID28711138		
N.A	N.A	FcgR	N.A	N.A	PMID20707220		
N.A	N.A	HLA-DRB1	N.A	N.A	PMID25919528		
N.A	N.A	IFI16	N.A	N.A	PMID29341486		
N.A	N.A	IL17A	N.A	N.A	PMID29584788		
N.A	N.A	LMP2	N.A	N.A	PMID8730138		
N.A	N.A	SLC8A3	N.A	N.A	PMID28711138		
N.A	N.A	TBX3	N.A	N.A	PMID30630509		
N.A	N.A	TNFalpha	N.A	N.A	PMID33483748		
N.A	N.A	CSE	N.A	N.A	PMID29844591		
N.A	N.A	IFI144	N.A	N.A	PMID36189314		
N.A	N.A	NRAMP1	N.A	N.A	PMID12135431		
N.A	N.A	DR4	N.A	N.A	PMID3927858		
