# Supplementary material for: Integrative molecular network analysis of genetic risk factors to infer biomarkers and therapeutic targets for rheumatoid arthritis
Source: PLoS One. 2025 Aug 21;20(8):e0329101. doi: 10.1371/journal.pone.0329101 (PMC12370121; doi:10.1371/journal.pone.0329101)
Supplement: S2 Table — (PDF) [file pone.0329101.s002.pdf]

dbsnp.rsid	_id	_score	dbsnp._license	dbsnp.alleles	dbsnp.alt	dbsnp.chrom	dbsnp.hg38.end	dbsnp.hg38.start	dbsnp.ref	dbsnp.vartype	dbsnp.citations	dbsnp.gene.name	dbsnp.gene.symbol
rs998731	chr8:g.80183160	12.185324	http://bit.ly/2AqoLOc	[{'allele': 'C', 'freq	G	8	80183160	80183160	C	snv	[26659945, 3283	uncharacterized LOC105375920	LOC105375920
rs998731	chr8:g.80183160	10.722702	http://bit.ly/2AqoLOc	[{'allele': 'C', 'freq	T	8	80183160	80183160	C	snv	[26659945, 3283	uncharacterized LOC105375920	LOC105375920
rs9979383	chr21:g.3534346	17.165886	http://bit.ly/2AqoLOc	[{'allele': 'C', 'freq	G	21	35343463	35343463	C	snv	[26843965, 2689	N.A	N.A
rs9979383	chr21:g.3534346	14.293955	http://bit.ly/2AqoLOc	[{'allele': 'C', 'freq	T	21	35343463	35343463	C	snv	[26843965, 2689	N.A	N.A
rs9943599	chr11:g.9731194	12.141735	http://bit.ly/2AqoLOc	[{'allele': 'C', 'freq	G	11	9731194	9731194	C	snv	N.A	switching B cell complex subunit SWAP70	SWAP70
rs9943599	chr11:g.9731194	10.699627	http://bit.ly/2AqoLOc	[{'allele': 'C', 'freq	T	11	9731194	9731194	C	snv	N.A	switching B cell complex subunit SWAP70	SWAP70
rs9927316	chr16:g.8598279	21.582733	http://bit.ly/2AqoLOc	[{'allele': 'C', 'freq	T	16	85982795	85982795	C	snv	N.A	N.A	N.A
rs9927316	chr16:g.8598279	17.262783	http://bit.ly/2AqoLOc	[{'allele': 'C', 'freq	G	16	85982795	85982795	C	snv	N.A	N.A	N.A
rs9901455	chr17:g.3511853	1	http://bit.ly/2AqoLOc	[{'allele': 'G', 'freq	A	17	35118530	35118530	G	snv	25741868	N.A	N.A
rs9826828	chr3:g.13668321	12.161432	http://bit.ly/2AqoLOc	[{'allele': 'G', 'freq	A	3	136683218	136683218	G	snv	32831971	stromal antigen 1	STAG1
rs968567	chr11:g.6182809	12.185324	http://bit.ly/2AqoLOc	[{'allele': 'C', 'freq	T	11	61828092	61828092	C	snv	[19546342, 2042	fatty acid desaturase 2	FADS2
rs9603616	chr13:g.3979393	17.109528	http://bit.ly/2AqoLOc	[{'allele': 'C', 'freq	T	13	39793932	39793932	C	snv	[26993500, 3283	N.A	N.A
rs9557321	chr13:g.9986884	10.641802	http://bit.ly/2AqoLOc	[{'allele': 'T', 'freq'	C	13	99868847	99868847	T	snv	N.A	N.A	N.A
rs9532434	chr13:g.3978177	17.113205	http://bit.ly/2AqoLOc	[{'allele': 'T', 'freq'	A	13	39781776	39781776	T	snv	N.A	component of oligomeric golgi complex 6	COG6
rs9532434	chr13:g.3978177	14.244385	http://bit.ly/2AqoLOc	[{'allele': 'T', 'freq'	C	13	39781776	39781776	T	snv	N.A	component of oligomeric golgi complex 6	COG6
rs950918814	chr4:g.80031255	14.282234	http://bit.ly/2AqoLOc	[{'allele': 'G', 'freq	T	4	80031255	80031255	G	snv	N.A	ANTXR cell adhesion molecule 2	ANTXR2
rs950918814	chr4:g.80031255	14.244385	http://bit.ly/2AqoLOc	[{'allele': 'G', 'freq	A	4	80031255	80031255	G	snv	N.A	ANTXR cell adhesion molecule 2	ANTXR2
rs947474	chr10:g.6348488	14.31599	http://bit.ly/2AqoLOc	[{'allele': 'G', 'freq	T	10	6348488	6348488	G	snv	[18978792, 1907	long intergenic non-protein coding RNA 2656	LINC02656
rs947474	chr10:g.6348488	14.293186	http://bit.ly/2AqoLOc	[{'allele': 'G', 'freq	C	10	6348488	6348488	G	snv	[18978792, 1907	long intergenic non-protein coding RNA 2656	LINC02656
rs947474	chr10:g.6348488	12.210293	http://bit.ly/2AqoLOc	[{'allele': 'G', 'freq	A	10	6348488	6348488	G	snv	[18978792, 1907	long intergenic non-protein coding RNA 2656	LINC02656
rs940825	chr7:g.17167540	12.263285	http://bit.ly/2AqoLOc	[{'allele': 'T', 'freq'	G	7	17167540	17167540	T	snv	N.A	N.A	N.A
rs9378815	chr6:g.426155G	17.24686	http://bit.ly/2AqoLOc	[{'allele': 'G', 'freq	A	6	426155	426155	G	snv	N.A	N.A	N.A
rs9378815	chr6:g.426155G	14.256571	http://bit.ly/2AqoLOc	[{'allele': 'G', 'freq	T	6	426155	426155	G	snv	N.A	N.A	N.A
rs9378815	chr6:g.426155G	14.241655	http://bit.ly/2AqoLOc	[{'allele': 'G', 'freq	C	6	426155	426155	G	snv	N.A	N.A	N.A
rs9373594	chr6:g.14951343	17.180494	http://bit.ly/2AqoLOc	[{'allele': 'T', 'freq'	A	6	149513438	149513438	T	snv	N.A	peptidylprolyl isomerase like 4	PPIL4
rs9373594	chr6:g.14951343	14.321266	http://bit.ly/2AqoLOc	[{'allele': 'T', 'freq'	C	6	149513438	149513438	T	snv	N.A	peptidylprolyl isomerase like 4	PPIL4
rs9372120	chr6:g.10621966	14.244385	http://bit.ly/2AqoLOc	[{'allele': 'T', 'freq'	G	6	106219660	106219660	T	snv	27363682	autophagy related 5	ATG5
rs934734	chr2:g.65368452	10.70771	http://bit.ly/2AqoLOc	[{'allele': 'G', 'freq	C	2	65368452	65368452	G	snv	[20453842, 2051	sprouty related EVH1 domain containing 2	SPRED2
rs934734	chr2:g.65368452	9.453314	http://bit.ly/2AqoLOc	[{'allele': 'G', 'freq	A	2	65368452	65368452	G	snv	[20453842, 2051	sprouty related EVH1 domain containing 2	SPRED2
rs934734	chr2:g.65368452	9.443935	http://bit.ly/2AqoLOc	[{'allele': 'G', 'freq	T	2	65368452	65368452	G	snv	[20453842, 2051	sprouty related EVH1 domain containing 2	SPRED2
rs932036	chr4:g.26089240	17.164366	http://bit.ly/2AqoLOc	[{'allele': 'A', 'freq'	G	4	26089240	26089240	A	snv	N.A	uncharacterized LINC02357	LINC02357
rs932036	chr4:g.26089240	14.293955	http://bit.ly/2AqoLOc	[{'allele': 'A', 'freq'	T	4	26089240	26089240	A	snv	N.A	uncharacterized LINC02357	LINC02357
rs911760	chr9:g.5438435C	10.713871	http://bit.ly/2AqoLOc	[{'allele': 'C', 'freq	T	9	5438435	5438435	C	snv	N.A	plasminogen receptor with a C-terminal lysine	PLGRKT
rs911760	chr9:g.5438435C	10.663349	http://bit.ly/2AqoLOc	[{'allele': 'C', 'freq	A	9	5438435	5438435	C	snv	N.A	plasminogen receptor with a C-terminal lysine	PLGRKT
rs909685	chr22:g.3935166	17.164366	http://bit.ly/2AqoLOc	[{'allele': 'T', 'freq'	C	22	39351666	39351666	T	snv	[22936693, 2638	synaptogyrin 1	SYNGR1
rs909685	chr22:g.3935166	17.145582	http://bit.ly/2AqoLOc	[{'allele': 'T', 'freq'	G	22	39351666	39351666	T	snv	[22936693, 2638	synaptogyrin 1	SYNGR1
rs909685	chr22:g.3935166	14.245945	http://bit.ly/2AqoLOc	[{'allele': 'T', 'freq'	A	22	39351666	39351666	T	snv	[22936693, 2638	synaptogyrin 1	SYNGR1
rs9005	chr2:g.11313383	2.7203636	http://bit.ly/2AqoLOc	[{'allele': 'G', 'freq	A	2	113133835	113133835	G	snv	[19934104, 2035	interleukin 1 receptor antagonist	IL1RN
rs883220	chr1:g.38151199	10.635252	http://bit.ly/2AqoLOc	[{'allele': 'C', 'freq	A	1	38151199	38151199	C	snv	[26843965, 3283	uncharacterized LOC105378654	LOC105378654
rs874040	chr4:g.26106575	14.224991	http://bit.ly/2AqoLOc	[{'allele': 'G', 'freq	C	4	26106575	26106575	G	snv	[20453842, 2051	N.A	N.A
rs866205108	chr18:g.6234240	14.224991	http://bit.ly/2AqoLOc	[{'allele': 'C', 'freq	A	18	62342401	62342401	C	snv	N.A	TNF receptor superfamily member 11a	TNFRSF11A
rs8133843	chr21:g.3536594	17.212355	http://bit.ly/2AqoLOc	[{'allele': 'G', 'freq	T	21	35365944	35365944	G	snv	N.A	N.A	N.A
rs8133843	chr21:g.3536594	17.17861	http://bit.ly/2AqoLOc	[{'allele': 'G', 'freq	C	21	35365944	35365944	G	snv	N.A	N.A	N.A
rs8133843	chr21:g.3536594	14.31599	http://bit.ly/2AqoLOc	[{'allele': 'G', 'freq	A	21	35365944	35365944	G	snv	N.A	N.A	N.A
rs8126756	chr21:g.3340313	17.251127	http://bit.ly/2AqoLOc	[{'allele': 'T', 'freq'	G	21	33403138	33403138	T	snv	[22057826, 2669	interferon gamma receptor 2	IFNGR2
rs8126756	chr21:g.3340313	1.9933434	http://bit.ly/2AqoLOc	[{'allele': 'T', 'freq'	C	21	33403138	33403138	T	snv	[22057826, 2669	interferon gamma receptor 2	IFNGR2
rs8106598	chr19:g.5151468	14.244385	http://bit.ly/2AqoLOc	[{'allele': 'C', 'freq	T	19	51514686	51514686	C	snv	N.A	N.A	N.A
rs8083786	chr18:g.1288136	14.329393	http://bit.ly/2AqoLOc	[{'allele': 'A', 'freq'	G	18	12881362	12881362	A	snv	N.A	protein tyrosine phosphatase non-receptor type 2	PTPN2
rs8073171	chr17:g.5243944	17.233637	http://bit.ly/2AqoLOc	[{'allele': 'G', 'freq	C	17	5243944	5243944	G	snv	N.A	N.A	N.A
rs8073171	chr17:g.5243944	17.176159	http://bit.ly/2AqoLOc	[{'allele': 'G', 'freq	T	17	5243944	5243944	G	snv	N.A	N.A	N.A
rs8073171	chr17:g.5243944	14.282234	http://bit.ly/2AqoLOc	[{'allele': 'G', 'freq	A	17	5243944	5243944	G	snv	N.A	N.A	N.A
rs8043085	chr15:g.3853593	14.305805	http://bit.ly/2AqoLOc	[{'allele': 'G', 'freq	T	15	38535939	38535939	G	snv	[26843965, 2733	RAS guanyl releasing protein 1	RASGRP1
rs8032939	chr15:g.3854183	12.175369	http://bit.ly/2AqoLOc	[{'allele': 'T', 'freq'	C	15	38541832	38541832	T	snv	27804980	RAS guanyl releasing protein 1	RASGRP1
rs8026898	chr15:g.6969907	12.220966	http://bit.ly/2AqoLOc	[{'allele': 'G', 'freq	A	15	69699078	69699078	G	snv	[26843965, 3283	N.A	N.A
rs798000	chr1:g.11673807	12.141855	http://bit.ly/2AqoLOc	[{'allele': 'C', 'freq	T	1	116738074	116738074	C	snv	24968232	N.A	N.A
rs7943728	chr11:g.6177959	3.745533	http://bit.ly/2AqoLOc	[{'allele': 'G', 'freq	A	11	61779596	61779596	G	snv	33670313	myelin regulatory factor	MYRF
rs793108	chr10:g.3112617	9.450315	http://bit.ly/2AqoLOc	[{'allele': 'C', 'freq	T	10	31126177	31126177	C	snv	N.A	uncharacterized LOC105376481	LOC105376481
rs793095	chr10:g.3109704	8.225225	http://bit.ly/2AqoLOc	[{'allele': 'C', 'freq	G	10	31097045	31097045	C	snv	N.A	uncharacterized LOC105376481	LOC105376481
rs793095	chr10:g.3109704	7.4691353	http://bit.ly/2AqoLOc	[{'allele': 'C', 'freq	T	10	31097045	31097045	C	snv	N.A	uncharacterized LOC105376481	LOC105376481
rs79145843	chr11:g.7269384	17.2467	http://bit.ly/2AqoLOc	[{'allele': 'C', 'freq	A	11	72693848	72693848	C	snv	N.A	ArfGAP with RhoGAP domain, ankyrin repeat and	ARAP1

dbsnp.rsid	_id	_score	dbsnp._license	dbsnp.alleles	dbsnp.alt	dbsnp.chrom	dbsnp.hg38.end	dbsnp.hg38.start	dbsnp.ref	dbsnp.vartype	dbsnp.citations	dbsnp.gene.name	dbsnp.gene.symbol
rs79145843	chr11:g.7269384	6.9566174	http://bit.ly/2AqoLOc	[{'allele': 'C', 'freq	T	11	72693848	72693848	C	snv	N.A	ArfGAP with RhoGAP domain, ankyrin repeat and	ARAP1
rs7902146	chr10:g.6204127	14.307951	http://bit.ly/2AqoLOc	[{'allele': 'C', 'freq	G	10	62041271	62041271	C	snv	[20424228, 2236	AT-rich interaction domain 5B	ARID5B
rs7902146	chr10:g.6204127	14.234728	http://bit.ly/2AqoLOc	[{'allele': 'C', 'freq	A	10	62041271	62041271	C	snv	[20424228, 2236	AT-rich interaction domain 5B	ARID5B
rs7902146	chr10:g.6204127	12.128408	http://bit.ly/2AqoLOc	[{'allele': 'C', 'freq	T	10	62041271	62041271	C	snv	[20424228, 2236	AT-rich interaction domain 5B	ARID5B
rs7848647	chr9:g.11480676	14.31599	http://bit.ly/2AqoLOc	[{'allele': 'T', 'freq'	C	9	114806766	114806766	T	snv	[17663424, 1842	TNF superfamily member 15	TNFSF15
rs77574423	chr3:g.11943270	21.658997	http://bit.ly/2AqoLOc	[{'allele': 'G', 'freq	T	3	11943270	11943270	G	snv	N.A	N.A	N.A
rs77574423	chr3:g.11943270	17.119461	http://bit.ly/2AqoLOc	[{'allele': 'G', 'freq	A	3	11943270	11943270	G	snv	N.A	N.A	N.A
rs7752903	chr6:g.13790622	17.223833	http://bit.ly/2AqoLOc	[{'allele': 'T', 'freq'	G	6	137906227	137906227	T	snv	[19387456, 2133	N.A	N.A
rs77465633	chr12:g.1114957	17.165886	http://bit.ly/2AqoLOc	[{'allele': 'C', 'freq	T	12	111495741	111495741	C	snv	N.A	ataxin 2	ATXN2
rs77465633	chr12:g.1114957	14.199593	http://bit.ly/2AqoLOc	[{'allele': 'C', 'freq	A	12	111495741	111495741	C	snv	N.A	ataxin 2	ATXN2
rs7731626	chr5:g.56148856	14.244385	http://bit.ly/2AqoLOc	[{'allele': 'G', 'freq	C	5	56148856	56148856	G	snv	[26386125, 2847	ankyrin repeat domain 55	ANKRD55
rs7731626	chr5:g.56148856	14.244385	http://bit.ly/2AqoLOc	[{'allele': 'G', 'freq	C	5	56148856	56148856	G	snv	[26386125, 2847	ankyrin repeat domain 55	ANKRD55
rs7731626	chr5:g.56148856	12.154573	http://bit.ly/2AqoLOc	[{'allele': 'G', 'freq	A	5	56148856	56148856	G	snv	[26386125, 2847	ankyrin repeat domain 55	ANKRD55
rs7731626	chr5:g.56148856	12.154573	http://bit.ly/2AqoLOc	[{'allele': 'G', 'freq	A	5	56148856	56148856	G	snv	[26386125, 2847	ankyrin repeat domain 55	ANKRD55
rs773125	chr12:g.5600117	17.153454	http://bit.ly/2AqoLOc	[{'allele': 'A', 'freq'	C	12	56001170	56001170	A	snv	[26019233, 3283	sulfite oxidase	SUOX
rs773125	chr12:g.5600117	17.113205	http://bit.ly/2AqoLOc	[{'allele': 'A', 'freq'	T	12	56001170	56001170	A	snv	[26019233, 3283	sulfite oxidase	SUOX
rs773125	chr12:g.5600117	14.211805	http://bit.ly/2AqoLOc	[{'allele': 'A', 'freq'	G	12	56001170	56001170	A	snv	[26019233, 3283	sulfite oxidase	SUOX
rs77191406	chr6:g.13788170	14.329586	http://bit.ly/2AqoLOc	[{'allele': 'C', 'freq	T	6	137881704	137881704	C	snv	[27435953, 2829	TNF alpha induced protein 3	TNFAIP3
rs762574969	chr1:g.23563705	14.244385	http://bit.ly/2AqoLOc	[{'allele': 'CAA', 'freq': {'alspac': 0.9		1	235637059	235637057	CAA	del	N.A	G protein subunit gamma 4	GNG4
rs7574865	chr2:g.19109990	12.2564335	http://bit.ly/2AqoLOc	[{'allele': 'T', 'freq'	A	2	191099907	191099907	T	snv	[17804842, 1793	signal transducer and activator of transcription 4	STAT4
rs7574865	chr2:g.19109990	12.256277	http://bit.ly/2AqoLOc	[{'allele': 'T', 'freq'	A	2	191099907	191099907	T	snv	[17804842, 1793	signal transducer and activator of transcription 4	STAT4
rs7574865	chr2:g.19109990	10.61783	http://bit.ly/2AqoLOc	[{'allele': 'T', 'freq'	G	2	191099907	191099907	T	snv	[17804842, 1793	signal transducer and activator of transcription 4	STAT4
rs7574865	chr2:g.19109990	10.61783	http://bit.ly/2AqoLOc	[{'allele': 'T', 'freq'	G	2	191099907	191099907	T	snv	[17804842, 1793	signal transducer and activator of transcription 4	STAT4
rs7540342	chr1:g.38156596	17.153454	http://bit.ly/2AqoLOc	[{'allele': 'T', 'freq'	G	1	38156596	38156596	T	snv	N.A	uncharacterized LOC105378654	LOC105378654
rs7540342	chr1:g.38156596	14.211779	http://bit.ly/2AqoLOc	[{'allele': 'T', 'freq'	C	1	38156596	38156596	T	snv	N.A	uncharacterized LOC105378654	LOC105378654
rs74842123	chrX:g.79076899	21.638985	http://bit.ly/2AqoLOc	[{'allele': 'G', 'freq	C	X	79076899	79076899	G	snv	N.A	N.A	N.A
rs74842123	chrX:g.79076899	17.253649	http://bit.ly/2AqoLOc	[{'allele': 'G', 'freq	A	X	79076899	79076899	G	snv	N.A	N.A	N.A
rs740122	chr7:g.28126823	17.113205	http://bit.ly/2AqoLOc	[{'allele': 'G', 'freq	C	7	28126823	28126823	G	snv	N.A	JAZF zinc finger 1	JAZF1
rs740122	chr7:g.28126823	14.245218	http://bit.ly/2AqoLOc	[{'allele': 'G', 'freq	A	7	28126823	28126823	G	snv	N.A	JAZF zinc finger 1	JAZF1
rs734094	chr11:g.2301990	12.263285	http://bit.ly/2AqoLOc	[{'allele': 'G', 'freq	C	11	2301990	2301990	G	snv	N.A	N.A	N.A
rs734094	chr11:g.2301990	12.17345	http://bit.ly/2AqoLOc	[{'allele': 'G', 'freq	T	11	2301990	2301990	G	snv	N.A	N.A	N.A
rs734094	chr11:g.2301990	10.598057	http://bit.ly/2AqoLOc	[{'allele': 'G', 'freq	A	11	2301990	2301990	G	snv	N.A	N.A	N.A
rs73366469	chr7:g.74619286	17.13274	http://bit.ly/2AqoLOc	[{'allele': 'T', 'freq'	C	7	74619286	74619286	T	snv	[26808113, 2727	N.A	N.A
rs7324510	chr13:g.2843289	12.256231	http://bit.ly/2AqoLOc	[{'allele': 'C', 'freq	G	13	28432898	28432898	C	snv	[28323906, 3140	fms related receptor tyrosine kinase 1	FLT1
rs7324510	chr13:g.2843289	12.243217	http://bit.ly/2AqoLOc	[{'allele': 'C', 'freq	A	13	28432898	28432898	C	snv	[28323906, 3140	fms related receptor tyrosine kinase 1	FLT1
rs73194058	chr21:g.3339198	21.547617	http://bit.ly/2AqoLOc	[{'allele': 'C', 'freq	G	21	33391982	33391982	C	snv	32831971	N.A	N.A
rs73194058	chr21:g.3339198	17.23261	http://bit.ly/2AqoLOc	[{'allele': 'C', 'freq	A	21	33391982	33391982	C	snv	32831971	N.A	N.A
rs73081554	chr3:g.58317208	12.263285	http://bit.ly/2AqoLOc	[{'allele': 'C', 'freq	T	3	58317208	58317208	C	snv	33455918	N.A	N.A
rs73013527	chr11:g.1286270	17.246706	http://bit.ly/2AqoLOc	[{'allele': 'C', 'freq	T	11	128627057	128627057	C	snv	[26241881, 3187	N.A	N.A
rs73005423	chr11:g.1188103	17.23261	http://bit.ly/2AqoLOc	[{'allele': 'A', 'freq'	G	11	118810370	118810370	A	snv	N.A	N.A	N.A
rs72928038	chr6:g.90267049	14.330011	http://bit.ly/2AqoLOc	[{'allele': 'G', 'freq	A	6	90267049	90267049	G	snv	[22922229, 2402	BTB domain and CNC homolog 2	BACH2
rs7278257	chr21:g.4423388	17.153454	http://bit.ly/2AqoLOc	[{'allele': 'G', 'freq	A	21	44233881	44233881	G	snv	N.A	inducible T cell costimulator ligand	ICOSLG
rs7278257	chr21:g.4423388	14.256571	http://bit.ly/2AqoLOc	[{'allele': 'G', 'freq	C	21	44233881	44233881	G	snv	N.A	inducible T cell costimulator ligand	ICOSLG
rs72634030	chr17:g.5369285	14.234728	http://bit.ly/2AqoLOc	[{'allele': 'C', 'freq	A	17	5369285	5369285	C	snv	N.A	rabaptin, RAB GTPase binding effector protein 1	RABEP1
rs726288	chr10:g.7994721	10.629694	http://bit.ly/2AqoLOc	[{'allele': 'C', 'freq	T	10	79947217	79947217	C	snv	[21934714, 3187	surfactant protein D	SFTPD
rs7241016	chr18:g.1288020	14.333988	http://bit.ly/2AqoLOc	[{'allele': 'A', 'freq'	T	18	12880207	12880207	A	snv	N.A	protein tyrosine phosphatase non-receptor type 2	PTPN2
rs7241016	chr18:g.1288020	12.263285	http://bit.ly/2AqoLOc	[{'allele': 'A', 'freq'	G	18	12880207	12880207	A	snv	N.A	protein tyrosine phosphatase non-receptor type 2	PTPN2
rs7206670	chr16:g.1174003	14.329424	http://bit.ly/2AqoLOc	[{'allele': 'G', 'freq	C	16	11740030	11740030	G	snv	N.A	thioredoxin domain containing 11	TXNDC11
rs7206670	chr16:g.1174003	14.224991	http://bit.ly/2AqoLOc	[{'allele': 'G', 'freq	A	16	11740030	11740030	G	snv	N.A	thioredoxin domain containing 11	TXNDC11
rs7206670	chr16:g.1174003	12.210293	http://bit.ly/2AqoLOc	[{'allele': 'G', 'freq	T	16	11740030	11740030	G	snv	N.A	thioredoxin domain containing 11	TXNDC11
rs7171617	chr15:g.9036162	12.128408	http://bit.ly/2AqoLOc	[{'allele': 'G', 'freq	A	15	90361626	90361626	G	snv	N.A	zinc finger protein 774	ZNF774
rs71624119	chr5:g.56144903	17.164366	http://bit.ly/2AqoLOc	[{'allele': 'G', 'freq	C	5	56144903	56144903	G	snv	[26386125, 2689	ankyrin repeat domain 55	ANKRD55
rs71624119	chr5:g.56144903	17.164366	http://bit.ly/2AqoLOc	[{'allele': 'G', 'freq	C	5	56144903	56144903	G	snv	[26386125, 2689	ankyrin repeat domain 55	ANKRD55
rs71624119	chr5:g.56144903	14.293186	http://bit.ly/2AqoLOc	[{'allele': 'G', 'freq	A	5	56144903	56144903	G	snv	[26386125, 2689	ankyrin repeat domain 55	ANKRD55
rs71624119	chr5:g.56144903	14.293186	http://bit.ly/2AqoLOc	[{'allele': 'G', 'freq	A	5	56144903	56144903	G	snv	[26386125, 2689	ankyrin repeat domain 55	ANKRD55
rs7097397	chr10:g.4881735	10.639566	http://bit.ly/2AqoLOc	[{'allele': 'G', 'freq	C	10	48817351	48817351	G	snv	[20169177, 2377	WDFY family member 4	WDFY4
rs7097397	chr10:g.4881735	10.5793495	http://bit.ly/2AqoLOc	[{'allele': 'G', 'freq	T	10	48817351	48817351	G	snv	[20169177, 2377	WDFY family member 4	WDFY4
rs7097397	chr10:g.4881735	9.894014	http://bit.ly/2AqoLOc	[{'allele': 'G', 'freq	A	10	48817351	48817351	G	snv	[20169177, 2377	WDFY family member 4	WDFY4
rs706778	chr10:g.6056986	12.180801	http://bit.ly/2AqoLOc	[{'allele': 'C', 'freq	T	10	6056986	6056986	C	snv	[17395754, 1855	interleukin 2 receptor subunit alpha	IL2RA
rs705700	chr12:g.5599550	12.248341	http://bit.ly/2AqoLOc	[{'allele': 'T', 'freq'	A	12	55995509	55995509	T	snv	31036605	N.A	N.A

dbsnp.rsid	_id	_score	dbsnp._license	dbsnp.alleles	dbsnp.alt	dbsnp.chrom	dbsnp.hg38.end	dbsnp.hg38.start	dbsnp.ref	dbsnp.vartype	dbsnp.citations	dbsnp.gene.name	dbsnp.gene.symbol
rs705700	chr12:g.5599550	10.648554	http://bit.ly/2AqoLOc	[{'allele': 'T', 'freq'	C	12	55995509	55995509	T	snv	31036605	N.A	N.A
rs6979218	chr7:g.10029552	14.329586	http://bit.ly/2AqoLOc	[{'allele': 'C', 'freq'	T	7	100295525	100295525	C	snv	N.A	N.A	N.A
rs6979218	chr7:g.10029552	12.243217	http://bit.ly/2AqoLOc	[{'allele': 'C', 'freq'	G	7	100295525	100295525	C	snv	N.A	N.A	N.A
rs6932056	chr6:g.13792130	14.307951	http://bit.ly/2AqoLOc	[{'allele': 'T', 'freq'	C	6	137921300	137921300	T	snv	[19387456, 1941	N.A	N.A
rs6930468	chr6:g.426268A>	14.336613	http://bit.ly/2AqoLOc	[{'allele': 'A', 'freq'	G	6	426268	426268	A	snv	N.A	N.A	N.A
rs6920220	chr6:g.13768536	9.48501	http://bit.ly/2AqoLOc	[{'allele': 'G', 'freq	A	6	137685367	137685367	G	snv	[17982455, 1798	N.A	N.A
rs6859212	chr5:g.27308412	17.201046	http://bit.ly/2AqoLOc	[{'allele': 'T', 'freq'	A	5	27308412	27308412	T	snv	N.A	N.A	N.A
rs6814280	chr4:g.12212250	14.191605	http://bit.ly/2AqoLOc	[{'allele': 'A', 'freq'	G	4	122122507	122122507	A	snv	N.A	N.A	N.A
rs678347	chr8:g.10145137	17.23261	http://bit.ly/2AqoLOc	[{'allele': 'G', 'freq	C	8	101451374	101451374	G	snv	32831971	N.A	N.A
rs678347	chr8:g.10145137	14.245218	http://bit.ly/2AqoLOc	[{'allele': 'G', 'freq	A	8	101451374	101451374	G	snv	32831971	N.A	N.A
rs6732565	chr2:g.11085025	14.224991	http://bit.ly/2AqoLOc	[{'allele': 'A', 'freq'	T	2	110850255	110850255	A	snv	32831971	acyl-CoA oxidase like	ACOXL
rs6732565	chr2:g.11085025	12.129879	http://bit.ly/2AqoLOc	[{'allele': 'A', 'freq'	G	2	110850255	110850255	A	snv	32831971	acyl-CoA oxidase like	ACOXL
rs67318457	chr6:g.23924793	17.211634	http://bit.ly/2AqoLOc	[{'allele': 'A', 'freq'	G	6	23924793	23924793	A	snv	N.A	N.A	N.A
rs67318457	chr6:g.23924793	14.314938	http://bit.ly/2AqoLOc	[{'allele': 'A', 'freq'	C	6	23924793	23924793	A	snv	N.A	N.A	N.A
rs67250450	chr7:g.28135367	14.232042	http://bit.ly/2AqoLOc	[{'allele': 'T', 'freq'	A	7	28135367	28135367	T	snv	32831971	JAZF zinc finger 1	JAZF1
rs67250450	chr7:g.28135367	12.126574	http://bit.ly/2AqoLOc	[{'allele': 'T', 'freq'	C	7	28135367	28135367	T	snv	32831971	JAZF zinc finger 1	JAZF1
rs6715284	chr2:g.20128967	14.256571	http://bit.ly/2AqoLOc	[{'allele': 'C', 'freq	T	2	201289674	201289674	C	snv	32831971	flagellum associated containing coiled-coil domains	FLACC1
rs6715284	chr2:g.20128967	8.336067	http://bit.ly/2AqoLOc	[{'allele': 'C', 'freq	G	2	201289674	201289674	C	snv	32831971	flagellum associated containing coiled-coil domains	FLACC1
rs6705628	chr2:g.73981235	9.421297	http://bit.ly/2AqoLOc	[{'allele': 'C', 'freq	T	2	73981235	73981235	C	snv	27313952	N.A	N.A
rs66922517	chr21:g.3534268	8.535658	http://bit.ly/2AqoLOc	[{'allele': 'ATGCA	ATGCAA	21			ATGCAAATGCA	delins	N.A	N.A	N.A
rs6681482	chr1:g.17333750	10.583611	http://bit.ly/2AqoLOc	[{'allele': 'G', 'freq	A	1	173337507	173337507	G	snv	N.A	N.A	N.A
rs6619397	chrX:g.79110630	21.619312	http://bit.ly/2AqoLOc	[{'allele': 'T', 'freq'	C	X	79110630	79110630	T	snv	N.A	N.A	N.A
rs6619397	chrX:g.79110630	17.145582	http://bit.ly/2AqoLOc	[{'allele': 'T', 'freq'	A	X	79110630	79110630	T	snv	N.A	N.A	N.A
rs660442	chr11:g.6427552	10.685255	http://bit.ly/2AqoLOc	[{'allele': 'G', 'freq	C	11	64275525	64275525	G	snv	N.A	N.A	N.A
rs660442	chr11:g.6427552	9.420311	http://bit.ly/2AqoLOc	[{'allele': 'G', 'freq	A	11	64275525	64275525	G	snv	N.A	N.A	N.A
rs6583441	chr7:g.50322278	10.685255	http://bit.ly/2AqoLOc	[{'allele': 'A', 'freq'	T	7	50322278	50322278	A	snv	N.A	IKAROS family zinc finger 1	IKZF1
rs657075	chr5:g.13209442	10.598043	http://bit.ly/2AqoLOc	[{'allele': 'G', 'freq	A	5	132094425	132094425	G	snv	[23577190, 2684	N.A	N.A
rs6546146	chr2:g.65329190	14.244385	http://bit.ly/2AqoLOc	[{'allele': 'C', 'freq	T	2	65329190	65329190	C	snv	N.A	sprouty related EVH1 domain containing 2	SPRED2
rs6546146	chr2:g.65329190	12.235353	http://bit.ly/2AqoLOc	[{'allele': 'C', 'freq	A	2	65329190	65329190	C	snv	N.A	sprouty related EVH1 domain containing 2	SPRED2
rs6495979	chr15:g.3855515	12.272539	http://bit.ly/2AqoLOc	[{'allele': 'C', 'freq	T	15	38555158	38555158	C	snv	27804980	RAS guanyl releasing protein 1	RASGRP1
rs6479800	chr10:g.6227712	17.2473	http://bit.ly/2AqoLOc	[{'allele': 'G', 'freq	T	10	62277122	62277122	G	snv	N.A	N.A	N.A
rs6479800	chr10:g.6227712	17.120924	http://bit.ly/2AqoLOc	[{'allele': 'G', 'freq	A	10	62277122	62277122	G	snv	N.A	N.A	N.A
rs6479800	chr10:g.6227712	14.211805	http://bit.ly/2AqoLOc	[{'allele': 'G', 'freq	C	10	62277122	62277122	G	snv	N.A	N.A	N.A
rs6478109	chr9:g.11480648	12.210293	http://bit.ly/2AqoLOc	[{'allele': 'A', 'freq'	G	9	114806486	114806486	A	snv	[17663424, 1875	TNF superfamily member 15	TNFSF15
rs641252	chr11:g.1082575	1	http://bit.ly/2AqoLOc	[{'allele': 'A', 'freq'	C	11	108257592	108257592	A	snv	[22529920, 2299	ATM serine/threonine kinase	ATM
rs629326	chr6:g.15907568	10.586767	http://bit.ly/2AqoLOc	[{'allele': 'G', 'freq	T	6	159075681	159075681	G	snv	27338350	uncharacterized LOC112267968	LOC112267968
rs624988	chr1:g.11672116	17.110794	http://bit.ly/2AqoLOc	[{'allele': 'T', 'freq'	A	1	116721168	116721168	T	snv	26843965	N.A	N.A
rs624988	chr1:g.11672116	14.241655	http://bit.ly/2AqoLOc	[{'allele': 'T', 'freq'	C	1	116721168	116721168	T	snv	26843965	N.A	N.A
rs62264113	chr3:g.12757349	17.23261	http://bit.ly/2AqoLOc	[{'allele': 'G', 'freq	C	3	127573490	127573490	G	snv	N.A	transmembrane protein adipocyte associated 1	TPRA1
rs62264113	chr3:g.12757349	7.9496937	http://bit.ly/2AqoLOc	[{'allele': 'G', 'freq	A	3	127573490	127573490	G	snv	N.A	transmembrane protein adipocyte associated 1	TPRA1
rs61944750	chr13:g.2806079	14.259037	http://bit.ly/2AqoLOc	[{'allele': 'G', 'freq	A	13	28060796	28060796	G	snv	N.A	fms related receptor tyrosine kinase 3	FLT3
rs61828284	chr1:g.17333060	12.235353	http://bit.ly/2AqoLOc	[{'allele': 'C', 'freq	T	1	173330604	173330604	C	snv	N.A	N.A	N.A
rs6032662	chr20:g.4610567	14.245945	http://bit.ly/2AqoLOc	[{'allele': 'C', 'freq	A	20	46105671	46105671	C	snv	27338350	N.A	N.A
rs6032662	chr20:g.4610567	12.180801	http://bit.ly/2AqoLOc	[{'allele': 'C', 'freq	T	20	46105671	46105671	C	snv	27338350	N.A	N.A
rs6011186	chr20:g.6385265	17.188879	http://bit.ly/2AqoLOc	[{'allele': 'C', 'freq	T	20	63852655	63852655	C	snv	N.A	N.A	N.A
rs5987194	chrX:g.15403601	12.154573	http://bit.ly/2AqoLOc	[{'allele': 'C', 'freq	G	X	154036016	154036016	C	snv	N.A	methyl-CpG binding protein 2	MECP2
rs59578717	chr11:g.6909240	17.238781	http://bit.ly/2AqoLOc	[{'allele': 'GGCTA	GGCTAAGGGG	11			GGCTAAGGGG	delins	N.A	uncharacterized LOC107984344	LOC107984344
rs59578717	chr11:g.6909240	17.233637	http://bit.ly/2AqoLOc	[{'allele': 'GGCTA	GGCTAAGGGG	11			GGCTAAGGGG	delins	N.A	uncharacterized LOC107984344	LOC107984344
rs59578717	chr11:g.6909240	17.223833	http://bit.ly/2AqoLOc	[{'allele': 'GGCTA	GGCTAAGGGG	11			GGCTAAGGGG	delins	N.A	uncharacterized LOC107984344	LOC107984344
rs59578717	chr11:g.6909240	17.211634	http://bit.ly/2AqoLOc	[{'allele': 'GGCTA	GGCTAAGGGG	11			GGCTAAGGGG	delins	N.A	uncharacterized LOC107984344	LOC107984344
rs59578717	chr11:g.6909240	17.201046	http://bit.ly/2AqoLOc	[{'allele': 'GGCTA	GGCTAAGGGG	11			GGCTAAGGGG	delins	N.A	uncharacterized LOC107984344	LOC107984344
rs59578717	chr11:g.6909240	17.17861	http://bit.ly/2AqoLOc	[{'allele': 'GGCTA	GGCTAAGGGG	11			GGCTAAGGGG	delins	N.A	uncharacterized LOC107984344	LOC107984344
rs59578717	chr11:g.6909240	17.164366	http://bit.ly/2AqoLOc	[{'allele': 'GGCTA	GGCTAAGGGG	11			GGCTAAGGGG	delins	N.A	uncharacterized LOC107984344	LOC107984344
rs59578717	chr11:g.6909240	17.161749	http://bit.ly/2AqoLOc	[{'allele': 'GGCTA	GGCTAAGGGG	11			GGCTAAGGGG	delins	N.A	uncharacterized LOC107984344	LOC107984344
rs59578717	chr11:g.6909240	17.155005	http://bit.ly/2AqoLOc	[{'allele': 'GGCTA	GGCTAAGGGG	11			GGCTAAGGGG	delins	N.A	uncharacterized LOC107984344	LOC107984344
rs59578717	chr11:g.6909240	17.152428	http://bit.ly/2AqoLOc	[{'allele': 'GGCTA	GGCTAAGGGG	11			GGCTAAGGGG	delins	N.A	uncharacterized LOC107984344	LOC107984344
rs595158	chr11:g.6114210	14.224991	http://bit.ly/2AqoLOc	[{'allele': 'C', 'freq	T	11	61142109	61142109	C	snv	[26843965, 3283	VPS37C subunit of ESCRT-I	VPS37C
rs595158	chr11:g.6114210	12.128408	http://bit.ly/2AqoLOc	[{'allele': 'C', 'freq	A	11	61142109	61142109	C	snv	[26843965, 3283	VPS37C subunit of ESCRT-I	VPS37C
rs59466457	chr6:g.16712426	12.243217	http://bit.ly/2AqoLOc	[{'allele': 'A', 'freq'	G	6	167124266	167124266	A	snv	N.A	N.A	N.A
rs591549	chr18:g.3542249	10.610268	http://bit.ly/2AqoLOc	[{'allele': 'T', 'freq'	G	18	3542249	3542249	T	snv	N.A	DLG associated protein 1	DLGAP1

dbsnp.rsid	_id	_score	dbsnp._license	dbsnp.alleles	dbsnp.alt	dbsnp.chrom	dbsnp.hg38.end	dbsnp.hg38.start	dbsnp.ref	dbsnp.vartype	dbsnp.citations	dbsnp.gene.name	dbsnp.gene.symbol
rs591549	chr18:g.3542249	9.4939995	http://bit.ly/2AqoLOc	[{'allele': 'T', 'freq'	C	18	3542249	3542249	T	snv	N.A	DLG associated protein 1	DLGAP1
rs587781471	chr13:g.3233669	1	http://bit.ly/2AqoLOc	[{'allele': 'C', 'freq	G	13	32336694	32336694	C	snv	25741868	BRCA2 DNA repair associated	BRCA2
rs58107865	chr4:g.10814046	17.211634	http://bit.ly/2AqoLOc	[{'allele': 'G', 'freq	T	4	108140462	108140462	G	snv	N.A	lymphoid enhancer binding factor 1	LEF1
rs58107865	chr4:g.10814046	14.187836	http://bit.ly/2AqoLOc	[{'allele': 'G', 'freq	C	4	108140462	108140462	G	snv	N.A	lymphoid enhancer binding factor 1	LEF1
rs5756407	chr22:g.3692021	8.539988	http://bit.ly/2AqoLOc	[{'allele': 'T', 'freq'	G	22	36920217	36920217	T	snv	N.A	N.A	N.A
rs5756407	chr22:g.3692021	8.516718	http://bit.ly/2AqoLOc	[{'allele': 'T', 'freq'	A	22	36920217	36920217	T	snv	N.A	N.A	N.A
rs5756407	chr22:g.3692021	7.75305	http://bit.ly/2AqoLOc	[{'allele': 'T', 'freq'	C	22	36920217	36920217	T	snv	N.A	N.A	N.A
rs5754104	chr22:g.2156207	17.132929	http://bit.ly/2AqoLOc	[{'allele': 'G', 'freq	T	22	21562072	21562072	G	snv	N.A	ubiquitin conjugating enzyme E2 L3	UBE2L3
rs5754104	chr22:g.2156207	14.314938	http://bit.ly/2AqoLOc	[{'allele': 'G', 'freq	A	22	21562072	21562072	G	snv	N.A	ubiquitin conjugating enzyme E2 L3	UBE2L3
rs56787183	chr5:g.40499188	21.549582	http://bit.ly/2AqoLOc	[{'allele': 'G', 'freq	T	5	40499188	40499188	G	snv	N.A	N.A	N.A
rs56787183	chr5:g.40499188	17.165886	http://bit.ly/2AqoLOc	[{'allele': 'G', 'freq	A	5	40499188	40499188	G	snv	N.A	N.A	N.A
rs56750287	chr17:g.3990669	14.196209	http://bit.ly/2AqoLOc	[{'allele': 'A', 'freq'	C	17	39906691	39906691	A	snv	N.A	gasdermin B	GSDMB
rs55914168	chr17:g.4309318	1	http://bit.ly/2AqoLOc	[{'allele': 'G', 'freq	A	17	43093180	43093180	G	snv	[10923033, 1253	BRCA1 DNA repair associated	BRCA1
rs55762233	chr19:g.1925651	14.245218	http://bit.ly/2AqoLOc	[{'allele': 'C', 'freq	G	19	19256510	19256510	C	snv	N.A	hyaluronan and proteoglycan link protein 4	HAPLN4
rs548234	chr6:g.10612015	14.25194	http://bit.ly/2AqoLOc	[{'allele': 'C', 'freq	G	6	106120159	106120159	C	snv	[19898481, 2007	N.A	N.A
rs548234	chr6:g.10612015	13.9042015	http://bit.ly/2AqoLOc	[{'allele': 'C', 'freq	A	6	106120159	106120159	C	snv	[19898481, 2007	N.A	N.A
rs548234	chr6:g.10612015	11.892628	http://bit.ly/2AqoLOc	[{'allele': 'C', 'freq	T	6	106120159	106120159	C	snv	[19898481, 2007	N.A	N.A
rs534731345	chr5:g.13260344	1	http://bit.ly/2AqoLOc	[{'allele': 'T', 'freq'	C	5	132603444	132603444	T	snv	N.A	RAD50 double strand break repair protein	RAD50
rs502919	chr10:g.6475205	14.233133	http://bit.ly/2AqoLOc	[{'allele': 'T', 'freq'	C	10	6475205	6475205	T	snv	N.A	protein kinase C theta	PRKCQ
rs4963581	chr12:g.2466034	10.722702	http://bit.ly/2AqoLOc	[{'allele': 'G', 'freq	C	12	24660347	24660347	G	snv	N.A	uncharacterized LOC105369698	LOC105369698
rs4963581	chr12:g.2466034	9.43775	http://bit.ly/2AqoLOc	[{'allele': 'G', 'freq	A	12	24660347	24660347	G	snv	N.A	uncharacterized LOC105369698	LOC105369698
rs4938573	chr11:g.1188711	17.233637	http://bit.ly/2AqoLOc	[{'allele': 'C', 'freq	G	11	118871133	118871133	C	snv	[24968232, 2527	N.A	N.A
rs4938573	chr11:g.1188711	17.165134	http://bit.ly/2AqoLOc	[{'allele': 'C', 'freq	A	11	118871133	118871133	C	snv	[24968232, 2527	N.A	N.A
rs4938573	chr11:g.1188711	14.199593	http://bit.ly/2AqoLOc	[{'allele': 'C', 'freq	T	11	118871133	118871133	C	snv	[24968232, 2527	N.A	N.A
rs4840565	chr8:g.11488036	17.165134	http://bit.ly/2AqoLOc	[{'allele': 'G', 'freq	A	8	11488036	11488036	G	snv	[24702955, 2496	N.A	N.A
rs4840565	chr8:g.11488036	14.329393	http://bit.ly/2AqoLOc	[{'allele': 'G', 'freq	C	8	11488036	11488036	G	snv	[24702955, 2496	N.A	N.A
rs4810485	chr20:g.4611930	9.453314	http://bit.ly/2AqoLOc	[{'allele': 'T', 'freq'	G	20	46119308	46119308	T	snv	[18794853, 1915	CD40 molecule	CD40
rs4810485	chr20:g.4611930	9.384951	http://bit.ly/2AqoLOc	[{'allele': 'T', 'freq'	A	20	46119308	46119308	T	snv	[18794853, 1915	CD40 molecule	CD40
rs4809371	chr20:g.6384954	17.22592	http://bit.ly/2AqoLOc	[{'allele': 'C', 'freq	G	20	63849545	63849545	C	snv	N.A	uncharacterized C20orf181	C20orf181
rs4809371	chr20:g.6384954	14.198082	http://bit.ly/2AqoLOc	[{'allele': 'C', 'freq	T	20	63849545	63849545	C	snv	N.A	uncharacterized C20orf181	C20orf181
rs479777	chr11:g.6434000	9.420315	http://bit.ly/2AqoLOc	[{'allele': 'T', 'freq'	C	11	64340005	64340005	T	snv	32823753	N.A	N.A
rs4780401	chr16:g.1174547	17.171694	http://bit.ly/2AqoLOc	[{'allele': 'G', 'freq	C	16	11745470	11745470	G	snv	32831971	N.A	N.A
rs4780401	chr16:g.1174547	17.13274	http://bit.ly/2AqoLOc	[{'allele': 'G', 'freq	A	16	11745470	11745470	G	snv	32831971	N.A	N.A
rs4780401	chr16:g.1174547	14.211927	http://bit.ly/2AqoLOc	[{'allele': 'G', 'freq	T	16	11745470	11745470	G	snv	32831971	N.A	N.A
rs4690029	chr4:g.2721088T	14.198082	http://bit.ly/2AqoLOc	[{'allele': 'T', 'freq'	A	4	2721088	2721088	T	snv	N.A	family with sequence similarity 193 member A	FAM193A
rs4690029	chr4:g.2721088T	12.162506	http://bit.ly/2AqoLOc	[{'allele': 'T', 'freq'	C	4	2721088	2721088	T	snv	N.A	family with sequence similarity 193 member A	FAM193A
rs4687070	chr3:g.18958886	17.176159	http://bit.ly/2AqoLOc	[{'allele': 'A', 'freq'	T	3	189588861	189588861	A	snv	N.A	N.A	N.A
rs4655698	chr1:g.67332644	14.346088	http://bit.ly/2AqoLOc	[{'allele': 'T', 'freq'	C	1	67332644	67332644	T	snv	N.A	interleukin 12 receptor subunit beta 2	IL12RB2
rs4655698	chr1:g.67332644	14.26969	http://bit.ly/2AqoLOc	[{'allele': 'T', 'freq'	A	1	67332644	67332644	T	snv	N.A	interleukin 12 receptor subunit beta 2	IL12RB2
rs4655698	chr1:g.67332644	12.235353	http://bit.ly/2AqoLOc	[{'allele': 'T', 'freq'	G	1	67332644	67332644	T	snv	N.A	interleukin 12 receptor subunit beta 2	IL12RB2
rs4622308	chr12:g.5607540	12.220966	http://bit.ly/2AqoLOc	[{'allele': 'C', 'freq	T	12	56075401	56075401	C	snv	28494655	N.A	N.A
rs4602367	chr3:g.17012007	14.293186	http://bit.ly/2AqoLOc	[{'allele': 'A', 'freq'	C	3	17012007	17012007	A	snv	N.A	phospholipase C like 2	PLCL2
rs4602367	chr3:g.17012007	14.191605	http://bit.ly/2AqoLOc	[{'allele': 'A', 'freq'	T	3	17012007	17012007	A	snv	N.A	phospholipase C like 2	PLCL2
rs4602367	chr3:g.17012007	7.6419773	http://bit.ly/2AqoLOc	[{'allele': 'A', 'freq'	G	3	17012007	17012007	A	snv	N.A	phospholipase C like 2	PLCL2
rs4584833	chr16:g.1170490	10.648554	http://bit.ly/2AqoLOc	[{'allele': 'C', 'freq	A	16	11704902	11704902	C	snv	N.A	thioredoxin domain containing 11	TXNDC11
rs4584833	chr16:g.1170490	10.629059	http://bit.ly/2AqoLOc	[{'allele': 'C', 'freq	G	16	11704902	11704902	C	snv	N.A	thioredoxin domain containing 11	TXNDC11
rs4584833	chr16:g.1170490	9.384951	http://bit.ly/2AqoLOc	[{'allele': 'C', 'freq	T	16	11704902	11704902	C	snv	N.A	thioredoxin domain containing 11	TXNDC11
rs4452313	chr3:g.17005540	16.734375	http://bit.ly/2AqoLOc	[{'allele': 'A', 'freq'	C	3	17005540	17005540	A	snv	32831971	phospholipase C like 2	PLCL2
rs4452313	chr3:g.17005540	13.903788	http://bit.ly/2AqoLOc	[{'allele': 'A', 'freq'	T	3	17005540	17005540	A	snv	32831971	phospholipase C like 2	PLCL2
rs4409785	chr11:g.9557825	14.211779	http://bit.ly/2AqoLOc	[{'allele': 'T', 'freq'	C	11	95578258	95578258	T	snv	[22922229, 2687	N.A	N.A
rs4272	chr7:g.92607515	9.40262	http://bit.ly/2AqoLOc	[{'allele': 'A', 'freq'	T	7	92607515	92607515	A	snv	[21194676, 2145	cyclin dependent kinase 6	CDK6
rs4272	chr7:g.92607515	8.535658	http://bit.ly/2AqoLOc	[{'allele': 'A', 'freq'	G	7	92607515	92607515	A	snv	[21194676, 2145	cyclin dependent kinase 6	CDK6
rs42044	chr7:g.92620826	12.198084	http://bit.ly/2AqoLOc	[{'allele': 'T', 'freq'	G	7	92620826	92620826	T	snv	N.A	cyclin dependent kinase 6	CDK6
rs419598	chr2:g.11312963	2.2980957	http://bit.ly/2AqoLOc	[{'allele': 'T', 'freq'	C	2	113129630	113129630	T	snv	[16820586, 1684	interleukin 1 receptor antagonist	IL1RN
rs41313373	chr1:g.92474854	17.113205	http://bit.ly/2AqoLOc	[{'allele': 'C', 'freq	A	1	92474854	92474854	C	snv	N.A	growth factor independent 1 transcriptional repress	GFI1
rs41313373	chr1:g.92474854	14.266296	http://bit.ly/2AqoLOc	[{'allele': 'C', 'freq	T	1	92474854	92474854	C	snv	N.A	growth factor independent 1 transcriptional repress	GFI1
rs41269479	chr1:g.41701111	14.198082	http://bit.ly/2AqoLOc	[{'allele': 'G', 'freq	C	1	41701111	41701111	G	snv	N.A	HIVEP zinc finger 3	HIVEP3
rs41269479	chr1:g.41701111	12.164055	http://bit.ly/2AqoLOc	[{'allele': 'G', 'freq	A	1	41701111	41701111	G	snv	N.A	HIVEP zinc finger 3	HIVEP3
rs403214	chr5:g.10326800	12.161425	http://bit.ly/2AqoLOc	[{'allele': 'A', 'freq'	T	5	103268000	103268000	A	snv	N.A	macrophage immunometabolism regulator	MACIR
rs403214	chr5:g.10326800	10.713871	http://bit.ly/2AqoLOc	[{'allele': 'A', 'freq'	G	5	103268000	103268000	A	snv	N.A	macrophage immunometabolism regulator	MACIR

dbsnp.rsid	_id	_score	dbsnp._license	dbsnp.alleles	dbsnp.alt	dbsnp.chrom	dbsnp.hg38.end	dbsnp.hg38.start	dbsnp.ref	dbsnp.vartype	dbsnp.citations	dbsnp.gene.name	dbsnp.gene.symbol
rs403214	chr5:g.10326800	10.707184	http://bit.ly/2AqoLOc	[{'allele': 'A', 'freq'	C	5	103268000	103268000	A	snv	N.A	macrophage immunometabolism regulator	MACIR
rs3890745	chr1:g.2622185T	10.7073345	http://bit.ly/2AqoLOc	[{'allele': 'T', 'freq'	C	1	2622185	2622185	T	snv	[18794853, 1989	membrane metalloendopeptidase like 1	MMEL1
rs3825568	chr14:g.6879387	17.211634	http://bit.ly/2AqoLOc	[{'allele': 'C', 'freq	G	14	68793871	68793871	C	snv	N.A	ZFP36 ring finger protein like 1	ZFP36L1
rs3825568	chr14:g.6879387	14.305805	http://bit.ly/2AqoLOc	[{'allele': 'C', 'freq	T	14	68793871	68793871	C	snv	N.A	ZFP36 ring finger protein like 1	ZFP36L1
rs3824660	chr10:g.8062759	12.164055	http://bit.ly/2AqoLOc	[{'allele': 'C', 'freq	G	10	8062759	8062759	C	snv	N.A	GATA binding protein 3	GATA3
rs3824660	chr10:g.8062759	10.707134	http://bit.ly/2AqoLOc	[{'allele': 'C', 'freq	T	10	8062759	8062759	C	snv	N.A	GATA binding protein 3	GATA3
rs3810936	chr9:g.11479060	7.5711994	http://bit.ly/2AqoLOc	[{'allele': 'T', 'freq'	C	9	114790605	114790605	T	snv	[17663424, 1842	TNF superfamily member 15	TNFSF15
rs3807306	chr7:g.12894062	9.1830635	http://bit.ly/2AqoLOc	[{'allele': 'G', 'freq	C	7	128940626	128940626	G	snv	[15657875, 1718	interferon regulatory factor 5	IRF5
rs3807306	chr7:g.12894062	8.535658	http://bit.ly/2AqoLOc	[{'allele': 'G', 'freq	A	7	128940626	128940626	G	snv	[15657875, 1718	interferon regulatory factor 5	IRF5
rs3807306	chr7:g.12894062	8.254841	http://bit.ly/2AqoLOc	[{'allele': 'G', 'freq	T	7	128940626	128940626	G	snv	[15657875, 1718	interferon regulatory factor 5	IRF5
rs3806624	chr3:g.27723132	12.243217	http://bit.ly/2AqoLOc	[{'allele': 'A', 'freq'	T	3	27723132	27723132	A	snv	[19602281, 2081	eomesodermin	EOMES
rs3806624	chr3:g.27723132	10.610268	http://bit.ly/2AqoLOc	[{'allele': 'A', 'freq'	G	3	27723132	27723132	A	snv	[19602281, 2081	eomesodermin	EOMES
rs3804333	chr6:g.10627934	14.256571	http://bit.ly/2AqoLOc	[{'allele': 'C', 'freq	A	6	106279340	106279340	C	snv	22040902	autophagy related 5	ATG5
rs3804333	chr6:g.10627934	12.220966	http://bit.ly/2AqoLOc	[{'allele': 'C', 'freq	T	6	106279340	106279340	C	snv	22040902	autophagy related 5	ATG5
rs3783782	chr14:g.6147395	14.196209	http://bit.ly/2AqoLOc	[{'allele': 'G', 'freq	A	14	61473957	61473957	G	snv	31875586	protein kinase C eta	PRKCH
rs3781913	chr11:g.7266245	7.776531	http://bit.ly/2AqoLOc	[{'allele': 'T', 'freq'	G	11	72662452	72662452	T	snv	[23577190, 2684	N.A	N.A
rs3764880	chrX:g.12906707	8.852985	http://bit.ly/2AqoLOc	[{'allele': 'A', 'freq'	G	X	12906707	12906707	A	snv	[18605904, 1892	N.A	N.A
rs3764880	chrX:g.12906707	8.449535	http://bit.ly/2AqoLOc	[{'allele': 'A', 'freq'	T	X	12906707	12906707	A	snv	[18605904, 1892	N.A	N.A
rs3764879	chrX:g.12906578	10.629059	http://bit.ly/2AqoLOc	[{'allele': 'C', 'freq	T	X	12906578	12906578	C	snv	[18927625, 1894	N.A	N.A
rs3764879	chrX:g.12906578	9.43775	http://bit.ly/2AqoLOc	[{'allele': 'C', 'freq	G	X	12906578	12906578	C	snv	[18927625, 1894	N.A	N.A
rs3761847	chr9:g.12092796	13.837212	http://bit.ly/2AqoLOc	[{'allele': 'G', 'freq	C	9	120927961	120927961	G	snv	[17804836, 1822	TNF receptor associated factor 1	TRAF1
rs3761847	chr9:g.12092796	11.827368	http://bit.ly/2AqoLOc	[{'allele': 'G', 'freq	A	9	120927961	120927961	G	snv	[17804836, 1822	TNF receptor associated factor 1	TRAF1
rs3753389	chr1:g.16083736	14.282234	http://bit.ly/2AqoLOc	[{'allele': 'T', 'freq'	A	1	160837363	160837363	T	snv	[20345977, 2045	CD244 molecule	CD244
rs3753389	chr1:g.16083736	14.224991	http://bit.ly/2AqoLOc	[{'allele': 'T', 'freq'	G	1	160837363	160837363	T	snv	[20345977, 2045	CD244 molecule	CD244
rs3753389	chr1:g.16083736	12.248341	http://bit.ly/2AqoLOc	[{'allele': 'T', 'freq'	C	1	160837363	160837363	T	snv	[20345977, 2045	CD244 molecule	CD244
rs372017932	chr17:g.4309317	1	http://bit.ly/2AqoLOc	[{'allele': 'C', 'freq	T	17	43093179	43093179	C	snv	[16267036, 1994	BRCA1 DNA repair associated	BRCA1
rs35156883	chr22:g.4535027	10.65172	http://bit.ly/2AqoLOc	[{'allele': 'TTTTTT	TTTTTTT	22			TTTTTTTT	delins	N.A	structural maintenance of chromosomes 1B	SMC1B
rs35156883	chr22:g.4535027	10.598043	http://bit.ly/2AqoLOc	[{'allele': 'TTTTTT	TTTTTTTTTTT	22			TTTTTTTT	delins	N.A	structural maintenance of chromosomes 1B	SMC1B
rs35156883	chr22:g.4535027	10.2616005	http://bit.ly/2AqoLOc	[{'allele': 'TTTTTT	TTTTTTTTTT	22			TTTTTTTT	delins	N.A	structural maintenance of chromosomes 1B	SMC1B
rs35156883	chr22:g.4535027	10.2616005	http://bit.ly/2AqoLOc	[{'allele': 'TTTTTT	TTTTTTTTT	22			TTTTTTTT	delins	N.A	structural maintenance of chromosomes 1B	SMC1B
rs34695944	chr2:g.60897715	12.154573	http://bit.ly/2AqoLOc	[{'allele': 'T', 'freq'	C	2	60897715	60897715	T	snv	32831971	REL proto-oncogene, NF-kB subunit	REL
rs34536443	chr19:g.1035244	4.658821	http://bit.ly/2AqoLOc	[{'allele': 'G', 'freq	C	19	10352442	10352442	G	snv	[18270328, 1929	tyrosine kinase 2	TYK2
rs34480360	chr16:g.3078736	12.194769	http://bit.ly/2AqoLOc	[{'allele': 'G', 'freq	A	16	30787368	30787368	G	snv	N.A	N.A	N.A
rs3184504	chr12:g.1114468	9.486883	http://bit.ly/2AqoLOc	[{'allele': 'T', 'freq'	A	12	111446804	111446804	T	snv	[17554260, 1825	SH2B adaptor protein 3	SH2B3
rs3184504	chr12:g.1114468	9.40262	http://bit.ly/2AqoLOc	[{'allele': 'T', 'freq'	G	12	111446804	111446804	T	snv	[17554260, 1825	SH2B adaptor protein 3	SH2B3
rs3184504	chr12:g.1114468	2.5119514	http://bit.ly/2AqoLOc	[{'allele': 'T', 'freq'	C	12	111446804	111446804	T	snv	[17554260, 1825	SH2B adaptor protein 3	SH2B3
rs315952	chr2:g.11313272	8.468439	http://bit.ly/2AqoLOc	[{'allele': 'T', 'freq'	A	2	113132727	113132727	T	snv	[16820586, 1717	interleukin 1 receptor antagonist	IL1RN
rs315952	chr2:g.11313272	2.315319	http://bit.ly/2AqoLOc	[{'allele': 'T', 'freq'	C	2	113132727	113132727	T	snv	[16820586, 1717	interleukin 1 receptor antagonist	IL1RN
rs3134883	chr10:g.6058762	10.628252	http://bit.ly/2AqoLOc	[{'allele': 'G', 'freq	C	10	6058762	6058762	G	snv	[19956099, 1995	interleukin 2 receptor subunit alpha	IL2RA
rs3134883	chr10:g.6058762	9.40271	http://bit.ly/2AqoLOc	[{'allele': 'G', 'freq	A	10	6058762	6058762	G	snv	[19956099, 1995	interleukin 2 receptor subunit alpha	IL2RA
rs3125734	chr10:g.6219835	9.40262	http://bit.ly/2AqoLOc	[{'allele': 'T', 'freq'	G	10	62198353	62198353	T	snv	N.A	rhotekin 2	RTKN2
rs3125734	chr10:g.6219835	8.543114	http://bit.ly/2AqoLOc	[{'allele': 'T', 'freq'	C	10	62198353	62198353	T	snv	N.A	rhotekin 2	RTKN2
rs3093023	chr6:g.16712080	8.478025	http://bit.ly/2AqoLOc	[{'allele': 'G', 'freq	T	6	167120802	167120802	G	snv	[20453842, 2051	N.A	N.A
rs3093023	chr6:g.16712080	8.460394	http://bit.ly/2AqoLOc	[{'allele': 'G', 'freq	C	6	167120802	167120802	G	snv	[20453842, 2051	N.A	N.A
rs3093023	chr6:g.16712080	7.714218	http://bit.ly/2AqoLOc	[{'allele': 'G', 'freq	A	6	167120802	167120802	G	snv	[20453842, 2051	N.A	N.A
rs3087243	chr2:g.20387419	10.707184	http://bit.ly/2AqoLOc	[{'allele': 'G', 'freq	T	2	203874196	203874196	G	snv	[15452244, 1638	cytotoxic T-lymphocyte associated protein 4	CTLA4
rs3087243	chr2:g.20387419	4.891577	http://bit.ly/2AqoLOc	[{'allele': 'G', 'freq	A	2	203874196	203874196	G	snv	[15452244, 1638	cytotoxic T-lymphocyte associated protein 4	CTLA4
rs3001423	chr14:g.1049201	12.233271	http://bit.ly/2AqoLOc	[{'allele': 'G', 'freq	C	14	104920174	104920174	G	snv	N.A	N.A	N.A
rs3001423	chr14:g.1049201	10.699627	http://bit.ly/2AqoLOc	[{'allele': 'G', 'freq	A	14	104920174	104920174	G	snv	N.A	N.A	N.A
rs2918392	chr5:g.10704685	14.293186	http://bit.ly/2AqoLOc	[{'allele': 'T', 'freq'	A	5	10704685	10704685	T	snv	N.A	death associated protein	DAP
rs2918392	chr5:g.10704685	14.211779	http://bit.ly/2AqoLOc	[{'allele': 'T', 'freq'	G	5	10704685	10704685	T	snv	N.A	death associated protein	DAP
rs2918392	chr5:g.10704685	12.198084	http://bit.ly/2AqoLOc	[{'allele': 'T', 'freq'	C	5	10704685	10704685	T	snv	N.A	death associated protein	DAP
rs2910164	chr5:g.16048541	12.857092	http://bit.ly/2AqoLOc	[{'allele': 'C', 'freq	G	5	160485411	160485411	C	snv	[18474871, 1852	N.A	N.A
rs2872507	chr17:g.3988451	14.196209	http://bit.ly/2AqoLOc	[{'allele': 'G', 'freq	T	17	39884510	39884510	G	snv	[18587394, 1906	N.A	N.A
rs2872507	chr17:g.3988451	12.141737	http://bit.ly/2AqoLOc	[{'allele': 'G', 'freq	A	17	39884510	39884510	G	snv	[18587394, 1906	N.A	N.A
rs2867461	chr4:g.78592061	14.293955	http://bit.ly/2AqoLOc	[{'allele': 'A', 'freq'	C	4	78592061	78592061	A	snv	[23577190, 2647	annexin A3	ANXA3
rs2867461	chr4:g.78592061	14.234728	http://bit.ly/2AqoLOc	[{'allele': 'A', 'freq'	T	4	78592061	78592061	A	snv	[23577190, 2647	annexin A3	ANXA3
rs2867461	chr4:g.78592061	12.174286	http://bit.ly/2AqoLOc	[{'allele': 'A', 'freq'	G	4	78592061	78592061	A	snv	[23577190, 2647	annexin A3	ANXA3
rs2847297	chr18:g.1279769	10.629059	http://bit.ly/2AqoLOc	[{'allele': 'A', 'freq'	G	18	12797695	12797695	A	snv	[20722033, 2133	protein tyrosine phosphatase non-receptor type 2	PTPN2
rs2843401	chr1:g.2596694T	12.141735	http://bit.ly/2AqoLOc	[{'allele': 'T', 'freq'	G	1	2596694	2596694	T	snv	32831971	membrane metalloendopeptidase like 1	MMEL1

dbsnp.rsid	_id	_score	dbsnp._license	dbsnp.alleles	dbsnp.alt	dbsnp.chrom	dbsnp.hg38.end	dbsnp.hg38.start	dbsnp.ref	dbsnp.vartype	dbsnp.citations	dbsnp.gene.name	dbsnp.gene.symbol
rs2843401	chr1:g.2596694T	10.454222	http://bit.ly/2AqoLOc	[{'allele': 'T', 'freq'	C	1	2596694	2596694	T	snv	32831971	membrane metalloendopeptidase like 1	MMEL1
rs2841277	chr14:g.1049246	12.126574	http://bit.ly/2AqoLOc	[{'allele': 'C', 'freq	A	14	104924668	104924668	C	snv	[23124809, 2357	phospholipase D family member 4	PLD4
rs2841277	chr14:g.1049246	10.628252	http://bit.ly/2AqoLOc	[{'allele': 'C', 'freq	T	14	104924668	104924668	C	snv	[23124809, 2357	phospholipase D family member 4	PLD4
rs28411352	chr1:g.37812907	14.256571	http://bit.ly/2AqoLOc	[{'allele': 'C', 'freq	T	1	37812907	37812907	C	snv	32831971	metal regulatory transcription factor 1	MTF1
rs28398409	chr1:g.19861489	17.145582	http://bit.ly/2AqoLOc	[{'allele': 'A', 'freq'	G	1	198614892	198614892	A	snv	N.A	N.A	N.A
rs28373672	chr19:g.3572217	17.145582	http://bit.ly/2AqoLOc	[{'allele': 'A', 'freq'	T	19	35722170	35722170	A	snv	N.A	lysine methyltransferase 2B	KMT2B
rs28373672	chr19:g.3572217	3.26865	http://bit.ly/2AqoLOc	[{'allele': 'A', 'freq'	G	19	35722170	35722170	A	snv	N.A	lysine methyltransferase 2B	KMT2B
rs28362855	chr6:g.44266884	12.242195	http://bit.ly/2AqoLOc	[{'allele': 'G', 'freq	A	6	44266884	44266884	G	snv	N.A	N.A	N.A
rs28362855	chr6:g.44266884	10.722702	http://bit.ly/2AqoLOc	[{'allele': 'G', 'freq	T	6	44266884	44266884	G	snv	N.A	N.A	N.A
rs2833522	chr21:g.3180705	14.282234	http://bit.ly/2AqoLOc	[{'allele': 'G', 'freq	A	21	31807059	31807059	G	snv	26077402	N.A	N.A
rs2833522	chr21:g.3180705	14.259037	http://bit.ly/2AqoLOc	[{'allele': 'G', 'freq	C	21	31807059	31807059	G	snv	26077402	N.A	N.A
rs2812378	chr9:g.34710263	10.707184	http://bit.ly/2AqoLOc	[{'allele': 'G', 'freq	C	9	34710263	34710263	G	snv	[18794853, 1918	C-C motif chemokine ligand 21	CCL21
rs2812378	chr9:g.34710263	9.382816	http://bit.ly/2AqoLOc	[{'allele': 'G', 'freq	A	9	34710263	34710263	G	snv	[18794853, 1918	C-C motif chemokine ligand 21	CCL21
rs2736340	chr8:g.11486464	17.176159	http://bit.ly/2AqoLOc	[{'allele': 'C', 'freq	G	8	11486464	11486464	C	snv	[19503088, 1964	N.A	N.A
rs2736340	chr8:g.11486464	17.171694	http://bit.ly/2AqoLOc	[{'allele': 'C', 'freq	A	8	11486464	11486464	C	snv	[19503088, 1964	N.A	N.A
rs2736340	chr8:g.11486464	14.224991	http://bit.ly/2AqoLOc	[{'allele': 'C', 'freq	T	8	11486464	11486464	C	snv	[19503088, 1964	N.A	N.A
rs2671692	chr10:g.4888977	14.293186	http://bit.ly/2AqoLOc	[{'allele': 'G', 'freq	T	10	48889774	48889774	G	snv	[20017967, 2001	WDFY family member 4	WDFY4
rs2671692	chr10:g.4888977	12.128408	http://bit.ly/2AqoLOc	[{'allele': 'G', 'freq	A	10	48889774	48889774	G	snv	[20017967, 2001	WDFY family member 4	WDFY4
rs2664035	chr4:g.48218822	12.129879	http://bit.ly/2AqoLOc	[{'allele': 'G', 'freq	A	4	48218822	48218822	G	snv	32831971	tec protein tyrosine kinase	TEC
rs2582532	chr14:g.1049265	12.256277	http://bit.ly/2AqoLOc	[{'allele': 'T', 'freq'	C	14	104926500	104926500	T	snv	N.A	phospholipase D family member 4	PLD4
rs2561477	chr5:g.10327322	12.119709	http://bit.ly/2AqoLOc	[{'allele': 'G', 'freq	A	5	103273223	103273223	G	snv	N.A	macrophage immunometabolism regulator	MACIR
rs2542151	chr18:g.1277994	10.673527	http://bit.ly/2AqoLOc	[{'allele': 'G', 'freq	T	18	12779948	12779948	G	snv	[17554260, 1822	N.A	N.A
rs2476601	chr1:g.11383494	7.75305	http://bit.ly/2AqoLOc	[{'allele': 'A', 'freq'	T	1	113834946	113834946	A	snv	[15004560, 1520	N.A	N.A
rs2476601	chr1:g.11383494	4.918457	http://bit.ly/2AqoLOc	[{'allele': 'A', 'freq'	G	1	113834946	113834946	A	snv	[15004560, 1520	N.A	N.A
rs2469434	chr18:g.6987681	9.474564	http://bit.ly/2AqoLOc	[{'allele': 'T', 'freq'	C	18	69876810	69876810	T	snv	31875586	CD226 molecule	CD226
rs2451258	chr6:g.15908556	10.639566	http://bit.ly/2AqoLOc	[{'allele': 'C', 'freq	A	6	159085568	159085568	C	snv	[23886662, 2565	uncharacterized LOC112267968	LOC112267968
rs2451258	chr6:g.15908556	10.635252	http://bit.ly/2AqoLOc	[{'allele': 'C', 'freq	G	6	159085568	159085568	C	snv	[23886662, 2565	uncharacterized LOC112267968	LOC112267968
rs2451258	chr6:g.15908556	9.432503	http://bit.ly/2AqoLOc	[{'allele': 'C', 'freq	T	6	159085568	159085568	C	snv	[23886662, 2565	uncharacterized LOC112267968	LOC112267968
rs244685	chr5:g.13408819	10.663349	http://bit.ly/2AqoLOc	[{'allele': 'T', 'freq'	A	5	134088199	134088199	T	snv	N.A	uncharacterized LOC105379185	LOC105379185
rs244685	chr5:g.13408819	9.453314	http://bit.ly/2AqoLOc	[{'allele': 'T', 'freq'	G	5	134088199	134088199	T	snv	N.A	uncharacterized LOC105379185	LOC105379185
rs244468	chr5:g.14322485	9.48501	http://bit.ly/2AqoLOc	[{'allele': 'A', 'freq'	C	5	143224856	143224856	A	snv	N.A	Rho GTPase activating protein 26	ARHGAP26
rs244468	chr5:g.14322485	9.473919	http://bit.ly/2AqoLOc	[{'allele': 'A', 'freq'	T	5	143224856	143224856	A	snv	N.A	Rho GTPase activating protein 26	ARHGAP26
rs244468	chr5:g.14322485	8.552681	http://bit.ly/2AqoLOc	[{'allele': 'A', 'freq'	G	5	143224856	143224856	A	snv	N.A	Rho GTPase activating protein 26	ARHGAP26
rs2317231	chr1:g.15771654	14.18914	http://bit.ly/2AqoLOc	[{'allele': 'G', 'freq	T	1	157716547	157716547	G	snv	N.A	N.A	N.A
rs2317230	chr1:g.15770520	17.24686	http://bit.ly/2AqoLOc	[{'allele': 'G', 'freq	T	1	157705207	157705207	G	snv	27140173	N.A	N.A
rs2305480	chr17:g.3990594	4.317501	http://bit.ly/2AqoLOc	[{'allele': 'G', 'freq	A	17	39905943	39905943	G	snv	[18923164, 1945	gasdermin B	GSDMB
rs2301888	chr1:g.17346235	12.221716	http://bit.ly/2AqoLOc	[{'allele': 'G', 'freq	A	1	17346235	17346235	G	snv	[27272985, 3283	peptidyl arginine deiminase 4	PADI4
rs2300373	chr21:g.3341643	17.171694	http://bit.ly/2AqoLOc	[{'allele': 'A', 'freq'	G	21	33416432	33416432	A	snv	N.A	interferon gamma receptor 2	IFNGR2
rs2300373	chr21:g.3341643	17.152433	http://bit.ly/2AqoLOc	[{'allele': 'A', 'freq'	C	21	33416432	33416432	A	snv	N.A	interferon gamma receptor 2	IFNGR2
rs2300373	chr21:g.3341643	14.329586	http://bit.ly/2AqoLOc	[{'allele': 'A', 'freq'	T	21	33416432	33416432	A	snv	N.A	interferon gamma receptor 2	IFNGR2
rs2275806	chr10:g.8053377	12.141855	http://bit.ly/2AqoLOc	[{'allele': 'G', 'freq	C	10	8053377	8053377	G	snv	[18410415, 2684	N.A	N.A
rs2275806	chr10:g.8053377	10.65172	http://bit.ly/2AqoLOc	[{'allele': 'G', 'freq	A	10	8053377	8053377	G	snv	[18410415, 2684	N.A	N.A
rs227163	chr1:g.7901146C	10.699627	http://bit.ly/2AqoLOc	[{'allele': 'C', 'freq	A	1	7901146	7901146	C	snv	[21559414, 3187	urotensin 2	UTS2
rs227163	chr1:g.7901146C	10.598043	http://bit.ly/2AqoLOc	[{'allele': 'C', 'freq	G	1	7901146	7901146	C	snv	[21559414, 3187	urotensin 2	UTS2
rs227163	chr1:g.7901146C	9.382816	http://bit.ly/2AqoLOc	[{'allele': 'C', 'freq	T	1	7901146	7901146	C	snv	[21559414, 3187	urotensin 2	UTS2
rs2258734	chr1:g.2552522G	12.256277	http://bit.ly/2AqoLOc	[{'allele': 'G', 'freq	C	1	2552522	2552522	G	snv	N.A	TNFRSF14 antisense RNA 1	TNFRSF14-AS1
rs2258734	chr1:g.2552522G	10.663349	http://bit.ly/2AqoLOc	[{'allele': 'G', 'freq	A	1	2552522	2552522	G	snv	N.A	TNFRSF14 antisense RNA 1	TNFRSF14-AS1
rs2240336	chr1:g.17347907	11.922869	http://bit.ly/2AqoLOc	[{'allele': 'C', 'freq	T	1	17347907	17347907	C	snv	[21698003, 2684	peptidyl arginine deiminase 4	PADI4
rs2234067	chr6:g.36387877	10.583611	http://bit.ly/2AqoLOc	[{'allele': 'A', 'freq'	T	6	36387877	36387877	A	snv	32831971	N.A	N.A
rs2234067	chr6:g.36387877	9.402603	http://bit.ly/2AqoLOc	[{'allele': 'A', 'freq'	C	6	36387877	36387877	A	snv	32831971	N.A	N.A
rs2233945	chr6:g.31139584	8.466198	http://bit.ly/2AqoLOc	[{'allele': 'C', 'freq	A	6	31139584	31139584	C	snv	[24322967, 2536	N.A	N.A
rs2233424	chr6:g.44266184	12.126574	http://bit.ly/2AqoLOc	[{'allele': 'C', 'freq	A	6	44266184	44266184	C	snv	N.A	N.A	N.A
rs2233424	chr6:g.44266184	10.693783	http://bit.ly/2AqoLOc	[{'allele': 'C', 'freq	G	6	44266184	44266184	C	snv	N.A	N.A	N.A
rs2233424	chr6:g.44266184	10.673527	http://bit.ly/2AqoLOc	[{'allele': 'C', 'freq	T	6	44266184	44266184	C	snv	N.A	N.A	N.A
rs2228145	chr1:g.15445449	8.516718	http://bit.ly/2AqoLOc	[{'allele': 'A', 'freq'	T	1	154454494	154454494	A	snv	[15306846, 1735	interleukin 6 receptor	IL6R
rs2228145	chr1:g.15445449	4.179987	http://bit.ly/2AqoLOc	[{'allele': 'A', 'freq'	C	1	154454494	154454494	A	snv	[15306846, 1735	interleukin 6 receptor	IL6R
rs2147161	chr13:g.4240816	12.272539	http://bit.ly/2AqoLOc	[{'allele': 'A', 'freq'	T	13	42408166	42408166	A	snv	N.A	N.A	N.A
rs2147161	chr13:g.4240816	12.220966	http://bit.ly/2AqoLOc	[{'allele': 'A', 'freq'	C	13	42408166	42408166	A	snv	N.A	N.A	N.A
rs2141331	chr2:g.20128676	2.3186417	http://bit.ly/2AqoLOc	[{'allele': 'C', 'freq	T	2	201286769	201286769	C	snv	N.A	caspase 8	CASP8
rs2105325	chr1:g.17338058	8.490061	http://bit.ly/2AqoLOc	[{'allele': 'A', 'freq'	T	1	173380586	173380586	A	snv	32831971	N.A	N.A

dbsnp.rsid	_id	_score	dbsnp._license	dbsnp.alleles	dbsnp.alt	dbsnp.chrom	dbsnp.hg38.end	dbsnp.hg38.start	dbsnp.ref	dbsnp.vartype	dbsnp.citations	dbsnp.gene.name	dbsnp.gene.symbol
rs2105325	chr1:g.17338058	8.477684	http://bit.ly/2AqoLOc	[{'allele': 'A', 'freq	G	1	173380586	173380586	A	snv	32831971	N.A	N.A
rs2105325	chr1:g.17338058	7.7690945	http://bit.ly/2AqoLOc	[{'allele': 'A', 'freq	C	1	173380586	173380586	A	snv	32831971	N.A	N.A
rs2075876	chr21:g.4428927	12.242195	http://bit.ly/2AqoLOc	[{'allele': 'G', 'freq	A	21	44289270	44289270	G	snv	[21505073, 2165	autoimmune regulator	AIRE
rs2069235	chr22:g.3935177	14.336613	http://bit.ly/2AqoLOc	[{'allele': 'G', 'freq	T	22	39351775	39351775	G	snv	26993500	synaptogyrin 1	SYNGR1
rs2069235	chr22:g.3935177	14.232042	http://bit.ly/2AqoLOc	[{'allele': 'G', 'freq	C	22	39351775	39351775	G	snv	26993500	synaptogyrin 1	SYNGR1
rs2069235	chr22:g.3935177	12.174967	http://bit.ly/2AqoLOc	[{'allele': 'G', 'freq	A	22	39351775	39351775	G	snv	26993500	synaptogyrin 1	SYNGR1
rs201408742	chrX:g.79209119	21.638985	http://bit.ly/2AqoLOc	[{'allele': 'C', 'freq	G	X	79209119	79209119	C	snv	N.A	N.A	N.A
rs201408742	chrX:g.79209119	17.17861	http://bit.ly/2AqoLOc	[{'allele': 'C', 'freq	A	X	79209119	79209119	C	snv	N.A	N.A	N.A
rs199894206	chr15:g.9049566	14.333988	http://bit.ly/2AqoLOc	[{'allele': 'TTTTTT	TTTTTTTTT	15			TTTTTTTTTTTT	delins	N.A	IQ motif containing GTPase activating protein 1	IQGAP1
rs199894206	chr15:g.9049565	14.245218	http://bit.ly/2AqoLOc	[{'allele': 'TTTTTT	TTTTTTTTTTTTT	15			TTTTTTTTTTTT	delins	N.A	IQ motif containing GTPase activating protein 1	IQGAP1
rs199894206	chr15:g.9049566	14.244385	http://bit.ly/2AqoLOc	[{'allele': 'TTTTTT	TTTTTTTTTTT	15			TTTTTTTTTTTT	delins	N.A	IQ motif containing GTPase activating protein 1	IQGAP1
rs199894206	chr15:g.9049566	14.233133	http://bit.ly/2AqoLOc	[{'allele': 'TTTTTT	TTTTTTTTTT	15			TTTTTTTTTTTT	delins	N.A	IQ motif containing GTPase activating protein 1	IQGAP1
rs199894206	chr15:g.9049566	14.211779	http://bit.ly/2AqoLOc	[{'allele': 'TTTTTT	TTTTTTTTTTTTT	15			TTTTTTTTTTTT	delins	N.A	IQ motif containing GTPase activating protein 1	IQGAP1
rs199894206	chr15:g.9049566	14.18914	http://bit.ly/2AqoLOc	[{'allele': 'TTTTTT	TTTTTTTTTTTTT	15			TTTTTTTTTTTT	delins	N.A	IQ motif containing GTPase activating protein 1	IQGAP1
rs1980422	chr2:g.20374567	12.129879	http://bit.ly/2AqoLOc	[{'allele': 'C', 'freq	A	2	203745673	203745673	C	snv	[19898481, 2007	N.A	N.A
rs1980422	chr2:g.20374567	10.685255	http://bit.ly/2AqoLOc	[{'allele': 'C', 'freq	T	2	203745673	203745673	C	snv	[19898481, 2007	N.A	N.A
rs1950897	chr14:g.6829342	14.196209	http://bit.ly/2AqoLOc	[{'allele': 'C', 'freq	G	14	68293424	68293424	C	snv	32831971	RAD51 paralog B	RAD51B
rs1950897	chr14:g.6829342	12.161425	http://bit.ly/2AqoLOc	[{'allele': 'C', 'freq	T	14	68293424	68293424	C	snv	32831971	RAD51 paralog B	RAD51B
rs1943199	chr18:g.7575089	17.212355	http://bit.ly/2AqoLOc	[{'allele': 'T', 'freq'	C	18	75750899	75750899	T	snv	N.A	N.A	N.A
rs1943199	chr18:g.7575089	17.117672	http://bit.ly/2AqoLOc	[{'allele': 'T', 'freq'	A	18	75750899	75750899	T	snv	N.A	N.A	N.A
rs1943199	chr18:g.7575089	14.211927	http://bit.ly/2AqoLOc	[{'allele': 'T', 'freq'	G	18	75750899	75750899	T	snv	N.A	N.A	N.A
rs1893592	chr21:g.4243495	10.586767	http://bit.ly/2AqoLOc	[{'allele': 'A', 'freq	G	21	42434957	42434957	A	snv	[22057235, 2584	ubiquitin associated and SH3 domain containing A	UBASH3A
rs1893592	chr21:g.4243495	2.9035187	http://bit.ly/2AqoLOc	[{'allele': 'A', 'freq	C	21	42434957	42434957	A	snv	[22057235, 2584	ubiquitin associated and SH3 domain containing A	UBASH3A
rs1885013	chr14:g.6828797	17.238781	http://bit.ly/2AqoLOc	[{'allele': 'G', 'freq	T	14	68287978	68287978	G	snv	N.A	RAD51 paralog B	RAD51B
rs1885013	chr14:g.6828797	17.166286	http://bit.ly/2AqoLOc	[{'allele': 'G', 'freq	C	14	68287978	68287978	G	snv	N.A	RAD51 paralog B	RAD51B
rs1885013	chr14:g.6828797	14.282234	http://bit.ly/2AqoLOc	[{'allele': 'G', 'freq	A	14	68287978	68287978	G	snv	N.A	RAD51 paralog B	RAD51B
rs1883832	chr20:g.4611834	10.694756	http://bit.ly/2AqoLOc	[{'allele': 'T', 'freq'	A	20	46118343	46118343	T	snv	[18045485, 1809	CD40 molecule	CD40
rs1883832	chr20:g.4611834	10.693783	http://bit.ly/2AqoLOc	[{'allele': 'T', 'freq'	G	20	46118343	46118343	T	snv	[18045485, 1809	CD40 molecule	CD40
rs1883832	chr20:g.4611834	3.2707634	http://bit.ly/2AqoLOc	[{'allele': 'T', 'freq'	C	20	46118343	46118343	T	snv	[18045485, 1809	CD40 molecule	CD40
rs1877030	chr17:g.3958390	17.155005	http://bit.ly/2AqoLOc	[{'allele': 'T', 'freq'	A	17	39583908	39583908	T	snv	N.A	N.A	N.A
rs1877030	chr17:g.3958390	14.293955	http://bit.ly/2AqoLOc	[{'allele': 'T', 'freq'	C	17	39583908	39583908	T	snv	N.A	N.A	N.A
rs187579	chr5:g.10326923	12.233271	http://bit.ly/2AqoLOc	[{'allele': 'A', 'freq	T	5	103269232	103269232	A	snv	N.A	macrophage immunometabolism regulator	MACIR
rs187579	chr5:g.10326923	12.118441	http://bit.ly/2AqoLOc	[{'allele': 'A', 'freq	G	5	103269232	103269232	A	snv	N.A	macrophage immunometabolism regulator	MACIR
rs187579	chr5:g.10326923	10.687241	http://bit.ly/2AqoLOc	[{'allele': 'A', 'freq	C	5	103269232	103269232	A	snv	N.A	macrophage immunometabolism regulator	MACIR
rs1858037	chr2:g.65371166	14.282234	http://bit.ly/2AqoLOc	[{'allele': 'T', 'freq'	C	2	65371166	65371166	T	snv	N.A	sprouty related EVH1 domain containing 2	SPRED2
rs1858037	chr2:g.65371166	12.220966	http://bit.ly/2AqoLOc	[{'allele': 'T', 'freq'	A	2	65371166	65371166	T	snv	N.A	sprouty related EVH1 domain containing 2	SPRED2
rs182199544	chr7:g.27044962	21.638985	http://bit.ly/2AqoLOc	[{'allele': 'C', 'freq	A	7	27044962	27044962	C	snv	N.A	N.A	N.A
rs182199544	chr7:g.27044962	17.166286	http://bit.ly/2AqoLOc	[{'allele': 'C', 'freq	T	7	27044962	27044962	C	snv	N.A	N.A	N.A
rs1800896	chr1:g.20677355	2.1327171	http://bit.ly/2AqoLOc	[{'allele': 'T', 'freq'	C	1	206773552	206773552	T	snv	[15726497, 1644	N.A	N.A
rs1800872	chr1:g.20677306	3.8152466	http://bit.ly/2AqoLOc	[{'allele': 'T', 'freq'	G	1	206773062	206773062	T	snv	[15726497, 1644	N.A	N.A
rs1800797	chr7:g.22726602	8.534794	http://bit.ly/2AqoLOc	[{'allele': 'A', 'freq	T	7	22726602	22726602	A	snv	[16449530, 1664	N.A	N.A
rs1800797	chr7:g.22726602	8.429804	http://bit.ly/2AqoLOc	[{'allele': 'A', 'freq	C	7	22726602	22726602	A	snv	[16449530, 1664	N.A	N.A
rs1800797	chr7:g.22726602	5.092169	http://bit.ly/2AqoLOc	[{'allele': 'A', 'freq	G	7	22726602	22726602	A	snv	[16449530, 1664	N.A	N.A
rs1800796	chr7:g.22726627	9.40271	http://bit.ly/2AqoLOc	[{'allele': 'G', 'freq	A	7	22726627	22726627	G	snv	[15726497, 1598	N.A	N.A
rs1800796	chr7:g.22726627	8.527176	http://bit.ly/2AqoLOc	[{'allele': 'G', 'freq	C	7	22726627	22726627	G	snv	[15726497, 1598	N.A	N.A
rs1800470	chr19:g.4135301	3.5197582	http://bit.ly/2AqoLOc	[{'allele': 'G', 'freq	A	19	41353016	41353016	G	snv	[15113441, 1517	transforming growth factor beta 1	TGFB1
rs1800470	chr19:g.4135301	2.1976204	http://bit.ly/2AqoLOc	[{'allele': 'G', 'freq	C	19	41353016	41353016	G	snv	[15113441, 1517	transforming growth factor beta 1	TGFB1
rs1800469	chr19:g.4135439	4.792942	http://bit.ly/2AqoLOc	[{'allele': 'A', 'freq	G	19	41354391	41354391	A	snv	[15113403, 1515	N.A	N.A
rs175714	chr14:g.7551551	14.31599	http://bit.ly/2AqoLOc	[{'allele': 'T', 'freq'	A	14	75515513	75515513	T	snv	N.A	uncharacterized LOC105370572	LOC105370572
rs175714	chr14:g.7551551	12.2216835	http://bit.ly/2AqoLOc	[{'allele': 'T', 'freq'	C	14	75515513	75515513	T	snv	N.A	uncharacterized LOC105370572	LOC105370572
rs1696466	chr12:g.5772261	17.253649	http://bit.ly/2AqoLOc	[{'allele': 'T', 'freq'	G	12	57722614	57722614	T	snv	N.A	N.A	N.A
rs1696466	chr12:g.5772261	17.171694	http://bit.ly/2AqoLOc	[{'allele': 'T', 'freq'	A	12	57722614	57722614	T	snv	N.A	N.A	N.A
rs1696466	chr12:g.5772261	14.314938	http://bit.ly/2AqoLOc	[{'allele': 'T', 'freq'	C	12	57722614	57722614	T	snv	N.A	N.A	N.A
rs16903108	chr8:g.12856423	14.199593	http://bit.ly/2AqoLOc	[{'allele': 'T', 'freq'	C	8	128564231	128564231	T	snv	N.A	long intergenic non-protein coding RNA 824	LINC00824
rs1571878	chr6:g.16712735	14.336613	http://bit.ly/2AqoLOc	[{'allele': 'C', 'freq	G	6	167127354	167127354	C	snv	N.A	N.A	N.A
rs1571878	chr6:g.16712735	12.189585	http://bit.ly/2AqoLOc	[{'allele': 'C', 'freq	T	6	167127354	167127354	C	snv	N.A	N.A	N.A
rs1538981	chr10:g.3112242	10.699627	http://bit.ly/2AqoLOc	[{'allele': 'C', 'freq	T	10	31122426	31122426	C	snv	N.A	uncharacterized LOC105376481	LOC105376481
rs1516971	chr8:g.12852985	14.329393	http://bit.ly/2AqoLOc	[{'allele': 'T', 'freq'	C	8	128529854	128529854	T	snv	32831971	long intergenic non-protein coding RNA 824	LINC00824
rs149041927	chr16:g.2385988	17.253649	http://bit.ly/2AqoLOc	[{'allele': 'AGAGA	AGAGAGAGAG/	16			AGAGAGAGAG/	delins	N.A	protein kinase C beta	PRKCB
rs149041927	chr16:g.2385988	17.153454	http://bit.ly/2AqoLOc	[{'allele': 'AGAGA	AGAGAGAGAG/	16			AGAGAGAGAG/	delins	N.A	protein kinase C beta	PRKCB

dbsnp.rsid	_id	_score	dbsnp._license	dbsnp.alleles	dbsnp.alt	dbsnp.chrom	dbsnp.hg38.end	dbsnp.hg38.start	dbsnp.ref	dbsnp.vartype	dbsnp.citations	dbsnp.gene.name	dbsnp.gene.symbol
rs149041927	chr16:g.2385988	17.113205	http://bit.ly/2AqoLOc	[{'allele': 'AGAGA	AGAGAGAGAG/	16			AGAGAGAGAG/	delins	N.A	protein kinase C beta	PRKCB
rs149041927	chr16:g.2385988	17.109528	http://bit.ly/2AqoLOc	[{'allele': 'AGAGA	AGAGAGAGAG/	16			AGAGAGAGAG/	delins	N.A	protein kinase C beta	PRKCB
rs147736385	chr15:g.2784502	1	http://bit.ly/2AqoLOc	[{'allele': 'G', 'freq	A	15	27845028	27845028	G	snv	N.A	OCA2 melanosomal transmembrane protein	OCA2
rs147622113	chr19:g.1066126	14.256571	http://bit.ly/2AqoLOc	[{'allele': 'C', 'freq	T	19	10661265	10661265	C	snv	N.A	interleukin enhancer binding factor 3	ILF3
rs146492555	chr14:g.6146002	14.31599	http://bit.ly/2AqoLOc	[{'allele': 'TTTTTT	TTTTTTTT	14			TTTTTTT	delins	N.A	protein kinase C eta	PRKCH
rs146492555	chr14:g.6146002	14.233133	http://bit.ly/2AqoLOc	[{'allele': 'TTTTTT	TTTTTT	14			TTTTTTT	delins	N.A	protein kinase C eta	PRKCH
rs143259280	chr2:g.69982037	10.711418	http://bit.ly/2AqoLOc	[{'allele': 'ATCCA	ATCCATCCATC	2			ATCCATCCATC	delins	N.A	N.A	N.A
rs143259280	chr2:g.69982037	10.693783	http://bit.ly/2AqoLOc	[{'allele': 'ATCCA	ATCCATCCATC	2			ATCCATCCATC	delins	N.A	N.A	N.A
rs143259280	chr2:g.69982037	10.687241	http://bit.ly/2AqoLOc	[{'allele': 'ATCCA	ATCCATCCATC	2			ATCCATCCATC	delins	N.A	N.A	N.A
rs143259280	chr2:g.69982037	10.685255	http://bit.ly/2AqoLOc	[{'allele': 'ATCCA	ATCCATCCATC	2			ATCCATCCATC	delins	N.A	N.A	N.A
rs143259280	chr2:g.69982037	10.639566	http://bit.ly/2AqoLOc	[{'allele': 'ATCCA	ATCCATCCATC	2			ATCCATCCATC	delins	N.A	N.A	N.A
rs143259280	chr2:g.69982037	10.619305	http://bit.ly/2AqoLOc	[{'allele': 'ATCCA	ATCCATCCATC	2			ATCCATCCATC	delins	N.A	N.A	N.A
rs143259280	chr2:g.69982037	10.61783	http://bit.ly/2AqoLOc	[{'allele': 'ATCCA	ATCCATCCATC	2			ATCCATCCATC	delins	N.A	N.A	N.A
rs143259280	chr2:g.69982037	10.598057	http://bit.ly/2AqoLOc	[{'allele': 'ATCCA	ATCCATCCATC	2			ATCCATCCATC	delins	N.A	N.A	N.A
rs143259280	chr2:g.69982037	10.598043	http://bit.ly/2AqoLOc	[{'allele': 'ATCCA	ATCCATCCATC	2			ATCCATCCATC	delins	N.A	N.A	N.A
rs143259280	chr2:g.69982037	10.2616005	http://bit.ly/2AqoLOc	[{'allele': 'ATCCA	ATCCATCCATC	2			ATCCATCCATC	delins	N.A	N.A	N.A
rs1427749	chr12:g.4597633	14.314938	http://bit.ly/2AqoLOc	[{'allele': 'C', 'freq	T	12	45976333	45976333	C	snv	N.A	SR-related CTD associated factor 11	SCAF11
rs1427749	chr12:g.4597633	12.210293	http://bit.ly/2AqoLOc	[{'allele': 'C', 'freq	G	12	45976333	45976333	C	snv	N.A	SR-related CTD associated factor 11	SCAF11
rs1422673	chr5:g.15105942	12.260717	http://bit.ly/2AqoLOc	[{'allele': 'C', 'freq	G	5	151059427	151059427	C	snv	[21963258, 2269	TNFAIP3 interacting protein 1	TNIP1
rs1422673	chr5:g.15105942	12.210293	http://bit.ly/2AqoLOc	[{'allele': 'C', 'freq	A	5	151059427	151059427	C	snv	[21963258, 2269	TNFAIP3 interacting protein 1	TNIP1
rs1422673	chr5:g.15105942	10.70771	http://bit.ly/2AqoLOc	[{'allele': 'C', 'freq	T	5	151059427	151059427	C	snv	[21963258, 2269	TNFAIP3 interacting protein 1	TNIP1
rs138193887	chr11:g.1080966	14.346088	http://bit.ly/2AqoLOc	[{'allele': 'A', 'freq'	G	11	108096623	108096623	A	snv	N.A	cullin 5	CUL5
rs13426947	chr2:g.19106852	14.329424	http://bit.ly/2AqoLOc	[{'allele': 'G', 'freq	A	2	191068528	191068528	G	snv	[25332064, 2685	signal transducer and activator of transcription 4	STAT4
rs13397	chrX:g.15398279	8.777958	http://bit.ly/2AqoLOc	[{'allele': 'G', 'freq	A	X	153982797	153982797	G	snv	[22057235, 2409	N.A	N.A
rs13330176	chr16:g.8598548	21.555641	http://bit.ly/2AqoLOc	[{'allele': 'T', 'freq'	G	16	85985481	85985481	T	snv	[26843965, 3283	N.A	N.A
rs13330176	chr16:g.8598548	21.549582	http://bit.ly/2AqoLOc	[{'allele': 'T', 'freq'	C	16	85985481	85985481	T	snv	[26843965, 3283	N.A	N.A
rs13330176	chr16:g.8598548	17.13281	http://bit.ly/2AqoLOc	[{'allele': 'T', 'freq'	A	16	85985481	85985481	T	snv	[26843965, 3283	N.A	N.A
rs13142500	chr4:g.10725733	17.171694	http://bit.ly/2AqoLOc	[{'allele': 'T', 'freq'	A	4	10725733	10725733	T	snv	N.A	cytokine dependent hematopoietic cell linker	CLNK
rs13142500	chr4:g.10725733	14.305805	http://bit.ly/2AqoLOc	[{'allele': 'T', 'freq'	C	4	10725733	10725733	T	snv	N.A	cytokine dependent hematopoietic cell linker	CLNK
rs13103285	chr4:g.10724896	17.176159	http://bit.ly/2AqoLOc	[{'allele': 'C', 'freq	A	4	10724896	10724896	C	snv	N.A	cytokine dependent hematopoietic cell linker	CLNK
rs13103285	chr4:g.10724896	17.145582	http://bit.ly/2AqoLOc	[{'allele': 'C', 'freq	G	4	10724896	10724896	C	snv	N.A	cytokine dependent hematopoietic cell linker	CLNK
rs13103285	chr4:g.10724896	14.244385	http://bit.ly/2AqoLOc	[{'allele': 'C', 'freq	T	4	10724896	10724896	C	snv	N.A	cytokine dependent hematopoietic cell linker	CLNK
rs13031237	chr2:g.60908994	10.687241	http://bit.ly/2AqoLOc	[{'allele': 'G', 'freq	T	2	60908994	60908994	G	snv	[19503088, 1994	REL proto-oncogene, NF-kB subunit	REL
rs12918327	chr16:g.3061529	17.17861	http://bit.ly/2AqoLOc	[{'allele': 'C', 'freq	T	16	30615295	30615295	C	snv	N.A	N.A	N.A
rs12795702	chr11:g.1282864	17.223833	http://bit.ly/2AqoLOc	[{'allele': 'A', 'freq'	C	11	128286419	128286419	A	snv	N.A	N.A	N.A
rs12795702	chr11:g.1282864	14.199593	http://bit.ly/2AqoLOc	[{'allele': 'A', 'freq'	G	11	128286419	128286419	A	snv	N.A	N.A	N.A
rs12764378	chr10:g.6204024	12.174967	http://bit.ly/2AqoLOc	[{'allele': 'G', 'freq	T	10	62040245	62040245	G	snv	[26843965, 3283	AT-rich interaction domain 5B	ARID5B
rs12764378	chr10:g.6204024	10.687241	http://bit.ly/2AqoLOc	[{'allele': 'G', 'freq	A	10	62040245	62040245	G	snv	[26843965, 3283	AT-rich interaction domain 5B	ARID5B
rs12712065	chr2:g.10014464	14.241655	http://bit.ly/2AqoLOc	[{'allele': 'C', 'freq	T	2	100144643	100144643	C	snv	N.A	N.A	N.A
rs12712065	chr2:g.10014464	12.242195	http://bit.ly/2AqoLOc	[{'allele': 'C', 'freq	G	2	100144643	100144643	C	snv	N.A	N.A	N.A
rs1264600	chr8:g.10144112	17.211634	http://bit.ly/2AqoLOc	[{'allele': 'T', 'freq'	C	8	101441122	101441122	T	snv	N.A	N.A	N.A
rs1264600	chr8:g.10144112	17.119461	http://bit.ly/2AqoLOc	[{'allele': 'T', 'freq'	A	8	101441122	101441122	T	snv	N.A	N.A	N.A
rs12530098	chr6:g.14106966	14.25194	http://bit.ly/2AqoLOc	[{'allele': 'C', 'freq	A	6	14106966	14106966	C	snv	N.A	uncharacterized LOC105374939	LOC105374939
rs12530098	chr6:g.14106966	12.235353	http://bit.ly/2AqoLOc	[{'allele': 'C', 'freq	T	6	14106966	14106966	C	snv	N.A	uncharacterized LOC105374939	LOC105374939
rs12529514	chr6:g.14096427	14.244385	http://bit.ly/2AqoLOc	[{'allele': 'T', 'freq'	C	6	14096427	14096427	T	snv	[23577190, 2684	N.A	N.A
rs12212067	chr6:g.10865999	10.707184	http://bit.ly/2AqoLOc	[{'allele': 'T', 'freq'	G	6	108659993	108659993	T	snv	[20306291, 2084	forkhead box O3	FOXO3
rs12145329	chr1:g.18663537	21.658997	http://bit.ly/2AqoLOc	[{'allele': 'T', 'freq'	G	1	186635373	186635373	T	snv	N.A	N.A	N.A
rs12145329	chr1:g.18663537	17.13274	http://bit.ly/2AqoLOc	[{'allele': 'T', 'freq'	C	1	186635373	186635373	T	snv	N.A	N.A	N.A
rs12126142	chr1:g.15445298	17.152433	http://bit.ly/2AqoLOc	[{'allele': 'G', 'freq	C	1	154452980	154452980	G	snv	N.A	interleukin 6 receptor	IL6R
rs12126142	chr1:g.15445298	14.25194	http://bit.ly/2AqoLOc	[{'allele': 'G', 'freq	A	1	154452980	154452980	G	snv	N.A	interleukin 6 receptor	IL6R
rs12026490	chr1:g.16044736	17.211634	http://bit.ly/2AqoLOc	[{'allele': 'T', 'freq'	C	1	160447367	160447367	T	snv	N.A	N.A	N.A
rs11933540	chr4:g.26118379	17.22592	http://bit.ly/2AqoLOc	[{'allele': 'T', 'freq'	C	4	26118379	26118379	T	snv	22199011	N.A	N.A
rs11900673	chr2:g.62225526	14.333988	http://bit.ly/2AqoLOc	[{'allele': 'C', 'freq	T	2	62225526	62225526	C	snv	[23577190, 2684	N.A	N.A
rs11777380	chr8:g.13319972	17.117672	http://bit.ly/2AqoLOc	[{'allele': 'G', 'freq	C	8	133199722	133199722	G	snv	N.A	cellular communication network factor 4	CCN4
rs11777380	chr8:g.13319972	14.198082	http://bit.ly/2AqoLOc	[{'allele': 'G', 'freq	A	8	133199722	133199722	G	snv	N.A	cellular communication network factor 4	CCN4
rs11586238	chr1:g.11672051	12.180801	http://bit.ly/2AqoLOc	[{'allele': 'C', 'freq	G	1	116720516	116720516	C	snv	[19898481, 2007	N.A	N.A
rs11574914	chr9:g.34710341	9.388975	http://bit.ly/2AqoLOc	[{'allele': 'G', 'freq	A	9	34710341	34710341	G	snv	[24493450, 2491	C-C motif chemokine ligand 21	CCL21
rs11571302	chr2:g.20387821	17.22592	http://bit.ly/2AqoLOc	[{'allele': 'G', 'freq	A	2	203878211	203878211	G	snv	[17280620, 1820	N.A	N.A
rs11571302	chr2:g.20387821	14.2609825	http://bit.ly/2AqoLOc	[{'allele': 'G', 'freq	T	2	203878211	203878211	G	snv	[17280620, 1820	N.A	N.A
rs115284761	chr15:g.7703449	14.336613	http://bit.ly/2AqoLOc	[{'allele': 'T', 'freq'	G	15	77034495	77034495	T	snv	N.A	proline-serine-threonine phosphatase interacting pı	PSTPIP1

dbsnp.rsid	_id	_score	dbsnp._license	dbsnp.alleles	dbsnp.alt	dbsnp.chrom	dbsnp.hg38.end	dbsnp.hg38.start	dbsnp.ref	dbsnp.vartype	dbsnp.citations	dbsnp.gene.name	dbsnp.gene.symbol
rs115284761	chr15:g.7703449	12.128408	http://bit.ly/2AqoLOc	[{'allele': 'T', 'freq'	C	15	77034495	77034495	T	snv	N.A	proline-serine-threonine phosphatase interacting pr	PSTPIP1
rs11454989	chr21:g.4428928	8.476402	http://bit.ly/2AqoLOc	[{'allele': 'CCC', 'f	CCCC	21			CCC	delins	N.A	autoimmune regulator	AIRE
rs1143634	chr2:g.11283281	5.5058136	http://bit.ly/2AqoLOc	[{'allele': 'G', 'freq	A	2	112832813	112832813	G	snv	[15726497, 1650	interleukin 1 beta	IL1B
rs1143627	chr2:g.11283681	5.590023	http://bit.ly/2AqoLOc	[{'allele': 'G', 'freq	A	2	112836810	112836810	G	snv	[10746728, 1134	interleukin 1 beta	IL1B
rs11420145	chr6:g.36414161	6.572605	http://bit.ly/2AqoLOc	[{'allele': 'AA', 'fre	AAA	6			AA	delins	N.A	peroxisomal testis enriched protein 1	PXT1
rs11375064	chr17:g.2757706	7.776531	http://bit.ly/2AqoLOc	[{'allele': 'TTTTTT	TTTTTTTTTTT	17			TTTTTTTTTTTT	delins	N.A	kinase suppressor of ras 1	KSR1
rs11375064	chr17:g.2757705	7.767023	http://bit.ly/2AqoLOc	[{'allele': 'TTTTTT	TTTTTTTTTTTTT	17			TTTTTTTTTTTT	delins	N.A	kinase suppressor of ras 1	KSR1
rs11375064	chr17:g.2757706	7.757128	http://bit.ly/2AqoLOc	[{'allele': 'TTTTTT	TTTTTTTTTTTTT	17			TTTTTTTTTTTT	delins	N.A	kinase suppressor of ras 1	KSR1
rs11375064	chr17:g.2757705	7.7357864	http://bit.ly/2AqoLOc	[{'allele': 'TTTTTT	TTTTTTTTTTTTT	17			TTTTTTTTTTTT	delins	N.A	kinase suppressor of ras 1	KSR1
rs11375064	chr17:g.2757705	7.703064	http://bit.ly/2AqoLOc	[{'allele': 'TTTTTT	TTTTTTTTTTTTT	17			TTTTTTTTTTTT	delins	N.A	kinase suppressor of ras 1	KSR1
rs113532504	chr6:g.15195451	17.262783	http://bit.ly/2AqoLOc	[{'allele': 'C', 'freq	T	6	15195451	15195451	C	snv	N.A	N.A	N.A
rs113066392	chr7:g.74611834	14.305805	http://bit.ly/2AqoLOc	[{'allele': 'CCC', 'f	CC	7			CCC	delins	N.A	N.A	N.A
rs11089637	chr22:g.2162480	17.110794	http://bit.ly/2AqoLOc	[{'allele': 'T', 'freq'	G	22	21624807	21624807	T	snv	N.A	N.A	N.A
rs11089637	chr22:g.2162480	14.282234	http://bit.ly/2AqoLOc	[{'allele': 'T', 'freq'	C	22	21624807	21624807	T	snv	N.A	N.A	N.A
rs10985070	chr9:g.12087384	17.13274	http://bit.ly/2AqoLOc	[{'allele': 'C', 'freq	G	9	120873843	120873843	C	snv	[17880261, 1864	PHD finger protein 19	PHF19
rs10985070	chr9:g.12087384	14.246359	http://bit.ly/2AqoLOc	[{'allele': 'C', 'freq	A	9	120873843	120873843	C	snv	[17880261, 1864	PHD finger protein 19	PHF19
rs10917571	chr1:g.16154962	14.198082	http://bit.ly/2AqoLOc	[{'allele': 'C', 'freq	T	1	161549621	161549621	C	snv	27338556	Fc fragment of IgG receptor IIIa	FCGR3A
rs10917571	chr1:g.16154962	12.174967	http://bit.ly/2AqoLOc	[{'allele': 'C', 'freq	A	1	161549621	161549621	C	snv	27338556	Fc fragment of IgG receptor IIIa	FCGR3A
rs10911902	chr1:g.18666318	12.126574	http://bit.ly/2AqoLOc	[{'allele': 'C', 'freq	T	1	186663185	186663185	C	snv	[18768181, 1996	N.A	N.A
rs10905284	chr10:g.8073399	14.259037	http://bit.ly/2AqoLOc	[{'allele': 'C', 'freq	G	10	8073399	8073399	C	snv	[26807920, 3055	GATA binding protein 3	GATA3
rs10905284	chr10:g.8073399	12.272539	http://bit.ly/2AqoLOc	[{'allele': 'C', 'freq	A	10	8073399	8073399	C	snv	[26807920, 3055	GATA binding protein 3	GATA3
rs10905284	chr10:g.8073399	12.119709	http://bit.ly/2AqoLOc	[{'allele': 'C', 'freq	T	10	8073399	8073399	C	snv	[26807920, 3055	GATA binding protein 3	GATA3
rs10892279	chr11:g.1187410	17.176159	http://bit.ly/2AqoLOc	[{'allele': 'G', 'freq	T	11	118741072	118741072	G	snv	[21383967, 2444	N.A	N.A
rs10892279	chr11:g.1187410	14.321266	http://bit.ly/2AqoLOc	[{'allele': 'G', 'freq	A	11	118741072	118741072	G	snv	[21383967, 2444	N.A	N.A
rs10821944	chr10:g.6202533	12.233271	http://bit.ly/2AqoLOc	[{'allele': 'G', 'freq	T	10	62025330	62025330	G	snv	[23577190, 2684	AT-rich interaction domain 5B	ARID5B
rs10790268	chr11:g.1188586	21.628277	http://bit.ly/2AqoLOc	[{'allele': 'A', 'freq'	T	11	118858682	118858682	A	snv	N.A	N.A	N.A
rs10790268	chr11:g.1188586	17.110794	http://bit.ly/2AqoLOc	[{'allele': 'A', 'freq'	G	11	118858682	118858682	A	snv	N.A	N.A	N.A
rs10774624	chr12:g.1113959	14.244385	http://bit.ly/2AqoLOc	[{'allele': 'G', 'freq	C	12	111395984	111395984	G	snv	[27744395, 3283	uncharacterized LINC02356	LINC02356
rs10774624	chr12:g.1113959	12.189585	http://bit.ly/2AqoLOc	[{'allele': 'G', 'freq	A	12	111395984	111395984	G	snv	[27744395, 3283	uncharacterized LINC02356	LINC02356
rs10683701	chr12:g.5769830	12.248341	http://bit.ly/2AqoLOc	[{'allele': 'ACTT',	ACTTACTT	12			ACTT	delins	24092415	OS9 endoplasmic reticulum lectin	OS9
rs10556591	chr11:g.1286162	8.449614	http://bit.ly/2AqoLOc	[{'allele': 'TGTG',	TG	11			TGTG	delins	N.A	uncharacterized LOC105369568	LOC105369568
rs10499194	chr6:g.13768150	17.165134	http://bit.ly/2AqoLOc	[{'allele': 'C', 'freq	T	6	137681500	137681500	C	snv	[17982456, 1879	N.A	N.A
rs10497813	chr2:g.19804934	12.162506	http://bit.ly/2AqoLOc	[{'allele': 'G', 'freq	T	2	198049348	198049348	G	snv	23817569	phospholipase C like 1 (inactive)	PLCL1
rs10488631	chr7:g.12895412	10.663349	http://bit.ly/2AqoLOc	[{'allele': 'T', 'freq'	C	7	128954129	128954129	T	snv	[17412832, 1756	transportin 3	TNPO3
rs10453119	chr8:g.80223447	17.246706	http://bit.ly/2AqoLOc	[{'allele': 'T', 'freq'	A	8	80223447	80223447	T	snv	N.A	uncharacterized LOC105375920	LOC105375920
rs10453119	chr8:g.80223447	14.346088	http://bit.ly/2AqoLOc	[{'allele': 'T', 'freq'	C	8	80223447	80223447	T	snv	N.A	uncharacterized LOC105375920	LOC105375920
rs1044165	chrX:g.66021884	8.516718	http://bit.ly/2AqoLOc	[{'allele': 'G', 'freq	A	X	66021884	66021884	G	snv	[15902657, 2343	V-set and immunoglobulin domain containing 4	VSIG4
rs10415976	chr19:g.941603A	9.443935	http://bit.ly/2AqoLOc	[{'allele': 'A', 'freq'	G	19	941603	941603	A	snv	30643196	AT-rich interaction domain 3A	ARID3A
rs10209110	chr2:g.10005623	14.199593	http://bit.ly/2AqoLOc	[{'allele': 'C', 'freq	T	2	100056230	100056230	C	snv	N.A	AF4/FMR2 family member 3	AFF3
rs10175798	chr2:g.30226728	17.262783	http://bit.ly/2AqoLOc	[{'allele': 'G', 'freq	T	2	30226728	30226728	G	snv	[27140173, 3283	N.A	N.A
rs10175798	chr2:g.30226728	14.241655	http://bit.ly/2AqoLOc	[{'allele': 'G', 'freq	A	2	30226728	30226728	G	snv	[27140173, 3283	N.A	N.A
