# Supplementary material for: Integrative molecular network analysis of genetic risk factors to infer biomarkers and therapeutic targets for rheumatoid arthritis
Source: PLoS One. 2025 Aug 21;20(8):e0329101. doi: 10.1371/journal.pone.0329101 (PMC12370121; doi:10.1371/journal.pone.0329101)
Supplement: S3 Table — (PDF) [file pone.0329101.s003.pdf]

Pasted	Symbol	Ensembl Gene ID	Entrez	Type	Species	Chr	Position (Mbp)	Description	centage_gc_cont	transcript_count	genomeSpan	cds_length	transcript_length	FiveUTR	ThreeUTR	nExons
PVT1	PVT1	NSG0000024985	NA	lncRNA	Human	8	127.794526	urce:HGNC Symb	45.87	176	392575	NA	1169.5	NA	NA	5
LINC02341	LINC02341	NSG0000028355	105370177	lncRNA	Human	13	42.339188	RNA 2341 [Source	40.21	2	146768	NA	1059.5	NA	NA	6
LINC01898	LINC01898	NSG0000026119	NA	lncRNA	Human	18	75.696044	RNA 1898 [Source	39.46	3	16807	NA	1903	NA	NA	2
MIR146A	MIR146A	NSG0000028373	406938	miRNA	Human	5	160.485352	urce:HGNC Symbo	45.45	1	98	NA	99	NA	NA	1
KIF4CP	KIF4CP	NSG0000022502	NA	cessed_pseudoge	Human	X	79.323446	dogene [Source:H	42.83	1	1771	NA	1279	NA	NA	3
TNFRSF9	TNFRSF9	NSG0000004924	3604	protein_coding	Human	1	7.915871	ber 9 [Source:HG	43.92	5	27294	346	607	83	174.5	5
MTF1	MTF1	NSG0000018878	4520	protein_coding	Human	1	37.809574	actor 1 [Source:H	43.6	2	50018	2262	7937	55.5	5561	6.5
POU3F1	POU3F1	NSG0000018566	5453	protein_coding	Human	1	38.043829	I [Source:HGNC S	63.14	1	2964	1356	2965	49	1558	1
HIVEP3	HIVEP3	NSG0000012712	59269	protein_coding	Human	1	41.506365	ource:HGNC Sym	46.45	7	529560	7218	12319	246	4085	5
IL12RB2	IL12RB2	NSG0000008198	3595	protein_coding	Human	1	67.307364	beta 2 [Source:H	41.73	8	91360	2331	3773	87	905	15
GFI1	GFI1	NSG0000016267	2672	protein_coding	Human	1	92.473043	onal repressor [So	53.67	4	13882	1269	2855	98	1266	7
PTPN22	PTPN22	NSG0000013424	26191	protein_coding	Human	1	113.813811	eptor type 22 [Sou	38.85	11	57942	474	2421	88	84.5	16
CD2	CD2	NSG0000011682	914	protein_coding	Human	1	116.75443	rce:HGNC Symbo	47.11	2	14799	1056	1565	43.5	247	3.5
IL6R	IL6R	NSG0000016071	3570	protein_coding	Human	1	154.405193	Source:HGNC Sy	49.12	8	64257	1078.5	1496	346	2890	6.5
FCRL3	FCRL3	NSG0000016085	115352	protein_coding	Human	1	157.674321	urce:HGNC Symb	40.66	12	26448	2205	2732	95	258.5	15
IFI16	IFI16	NSG0000016356	3428	protein_coding	Human	1	158.999968	tein 16 [Source:H	38.34	14	55187	732	2609	158	249	9.5
AIM2	AIM2	NSG0000016356	9447	protein_coding	Human	1	159.062484	[Source:HGNC S	40.1	4	84612	1032	832	48	207	5.5
CRP	CRP	NSG0000013269	1401	protein_coding	Human	1	159.712289	ource:HGNC Sym	48.59	7	2300	309	769	103	76	3
SLAMF6	SLAMF6	NSG0000016273	114836	protein_coding	Human	1	160.48503	Source:HGNC Sy	39.67	3	38232	996	2733	62	1670	8
CD244	CD244	NSG0000012222	51744	protein_coding	Human	1	160.83016	urce:HGNC Symb	43.81	5	32727	990	1341	52	163	9
FCGR3A	FCGR3A	NSG0000020374	2214	protein_coding	Human	1	161.541759	IIIa [Source:HGN	44.74	6	8978	765	2086	62.5	1207.5	5
TNFSF4	TNFSF4	NSG0000011758	7292	protein_coding	Human	1	173.183731	4 [Source:HGNC S	39.89	3	23600	477	3429	199	2782.5	3
PTGS2	PTGS2	NSG0000007375	5743	protein_coding	Human	1	186.671791	thase 2 [Source:H	36.06	6	9131	492	4506	132	246	10
LBH	LBH	NSG0000021362	81606	protein_coding	Human	2	30.231534	pathway [Source:H	46.96	9	92196	159	550	201	138	3
REL	REL	NSG0000016292	5966	protein_coding	Human	2	60.881521	ubunit [Source:H	37.61	3	50091	1764	11108	292.5	5475.5	10
B3GNT2	B3GNT2	NSG0000017034	10678	protein_coding	Human	2	62.196115	saminyltransferase	45.39	2	28616	1194	2598.5	52.5	1225	2
SPRED2	SPRED2	NSG0000019836	200734	protein_coding	Human	2	65.310851	taining 2 [Source:	43.76	8	121786	701	788.5	96	323	5
PCBP1	PCBP1	NSG0000016956	5093	protein_coding	Human	2	70.087477	[Source:HGNC S	54.89	1	1726	1071	1727	266	388	1
DGUOK	DGUOK	NSG0000011495	1716	protein_coding	Human	2	73.926826	[Source:HGNC S	42.81	8	32135	270	880	59.5	131	5
AFF3	AFF3	NSG0000014421	3899	protein_coding	Human	2	99.545419	3 [Source:HGNC	41.69	28	647009	431	730	79	139	5
ACOXL	ACOXL	NSG0000015309	55289	protein_coding	Human	2	110.732539	Source:HGNC Sy	42.77	11	388071	840	2188	80.5	453	6
IL1RN	IL1RN	NSG0000013668	3557	protein_coding	Human	2	113.107214	onist [Source:HGN	46.04	9	26802	432	1856.5	114	1143	4
DPP4	DPP4	NSG0000019763	1803	protein_coding	Human	2	161.992245	Source:HGNC Sy	39.64	23	82149	357	3375	85.5	80	24
STAT4	STAT4	NSG0000013837	6775	protein_coding	Human	2	191.029576	nscription 4 [Sour	37.27	11	122020	354	709	38	263	4
CASP8	CASP8	NSG0000006401	841	protein_coding	Human	2	201.233443	e:HGNC Symbol;	43.9	21	54268	837	1283	96	206	6
CD28	CD28	NSG0000017856	940	protein_coding	Human	2	203.706475	urce:HGNC Symb	40	3	32437	663	4526	139	3998	4
CTLA4	CTLA4	NSG0000016359	1493	protein_coding	Human	2	203.867771	I protein 4 [Source	42.23	6	6194	382.5	632.5	171	36	3
EOMES	EOMES	NSG0000016350	8320	protein_coding	Human	3	27.715949	urce:HGNC Symb	47.95	3	6762	2061	3264	115.5	1116	6
DNASE1L3	DNASE1L3	NSG0000016368	1776	protein_coding	Human	3	58.192257	3 [Source:HGNC	46.13	7	22440	828	1321	88	429	6
TPRA1	TPRA1	NSG0000016387	131601	protein_coding	Human	3	127.571232	sociated 1 [Sourc	54.83	12	27035	720	1725	133	527	9.5
IL20RB	IL20RB	NSG0000017456	53833	protein_coding	Human	3	136.94623	t beta [Source:HG	41.89	5	64855	168	1787	104	150	6
TPRG1	TPRG1	NSG0000018800	285386	protein_coding	Human	3	188.947214	1 [Source:HGNC	39.36	15	378090	828	1129	153	240	4
TP63	TP63	NSG0000007328	8626	protein_coding	Human	3	189.631389	urce:HGNC Symb	37.13	14	265887	1533	2032	125	1317	11
FAM193A	FAM193A	NSG0000012538	8603	protein_coding	Human	4	2.536647	member A [Source	45.62	11	195926	3636	4202	112	189	19
CLNK	CLNK	NSG0000010968	116449	protein_coding	Human	4	10.486395	cell linker [Source	41.51	4	198373	510	1539	52	1965.5	15.5
RBPJ	RBPJ	NSG0000016821	3516	protein_coding	Human	4	26.163455	globulin kappa J r	40.79	48	271676	472	1673	73	140	11
TEC	TEC	NSG0000013560	7006	protein_coding	Human	4	48.135783	e [Source:HGNC S	40.43	5	134055	303	2446	44	118	7
ANXA3	ANXA3	NSG0000013877	306	protein_coding	Human	4	78.551747	ce:HGNC Symbo	39.9	10	58704	855	1335	52	299	6
ANTXR2	ANTXR2	NSG0000016329	118429	protein_coding	Human	4	79.901146	le 2 [Source:HGN	35.81	13	224308	1227	2026	71	86	17
LEF1	LEF1	NSG0000013879	51176	protein_coding	Human	4	108.047545	ctor 1 [Source:HG	40.02	21	121411	990	1280	440	101	6
KIAA1109	KIAA1109	NSG0000013868	84162	protein_coding	Human	4	122.152331	e:HGNC Symbol;A	34.76	17	210427	10021.5	4967	87	503	18
DAP	DAP	NSG0000011297	1611	protein_coding	Human	5	10.67923	[Source:HGNC S	46.29	6	82004	462	1048	162	1048.5	3
PTGER4	PTGER4	NSG0000017152	5734	protein_coding	Human	5	40.679915	4 [Source:HGNC S	41.96	5	13820	1467	1279	302.5	1356	2
ANKRD55	ANKRD55	NSG0000016451	79722	protein_coding	Human	5	56.09968	[Source:HGNC S	41.57	6	133650	1348.5	1518	42.5	354.5	6.5
MACIR	MACIR	NSG0000018175	90355	protein_coding	Human	5	103.258763	regulator [Source:	37.42	3	19897	621	2933	89	1830	3
CSF2	CSF2	NSG0000016440	1437	protein_coding	Human	5	132.073789	2 [Source:HGNC S	55.5	1	2381	435	788	34	317	4
ARHGAP26	ARHGAP26	NSG0000014581	23092	protein_coding	Human	5	142.770377	n 26 [Source:HGN	42.17	23	458634	485	782	194	117	6
TNIP1	TNIP1	NSG0000014590	10318	protein_coding	Human	5	151.029945	1 [Source:HGNC	50.01	21	63632	1735.5	2238	35	193	16

IRF4	IRF4	NSG0000013726	3662	protein_coding	Human	6	0.391752	4 [Source:HGNC	48.32	5	19691	1233	1607	54	163	4
CD83	CD83	NSG0000011214	9308	protein_coding	Human	6	14.117256	urce:HGNC Symb	45.18	2	19662	441	2307	41	1675	5
JARID2	JARID2	NSG0000000808	3720	protein_coding	Human	6	15.246069	containing 2 [So	43.92	3	275973	3225	5595	138	1789	18
NRSN1	NRSN1	NSG0000015295	140767	protein_coding	Human	6	24.126186	e:HGNC Symbol;	40.22	7	28714	588	800	72	1582	3
ETV7	ETV7	NSG0000001003	51513	protein_coding	Human	6	36.354091	or 7 [Source:HGN	49.34	8	33709	795	1570	127	449	7
POLR1C	POLR1C	NSG0000017145	9533	protein_coding	Human	6	43.509702	nit C [Source:HG	45.68	16	52717	891	1307	20.5	153.5	9
BACH2	BACH2	NSG0000011218	60468	protein_coding	Human	6	89.926528	og 2 [Source:HGN	40.5	10	370380	2526	3981	112	5878	4
FOXO3	FOXO3	NSG0000011868	2309	protein_coding	Human	6	108.559835	urce:HGNC Symb	41.71	3	124939	2022	7296	180	478.5	3
TNFAIP3	TNFAIP3	NSG0000011850	7128	protein_coding	Human	6	137.867214	3 [Source:HGNC	43.95	11	16098	1504.5	3817	16	919	7
PPIL4	PPIL4	NSG0000013101	85313	protein_coding	Human	6	149.504495	4 [Source:HGNC	38.44	2	41548	931.5	1782	37	563.5	8
TAGAP	TAGAP	NSG0000016469	117289	protein_coding	Human	6	159.034468	ng protein [Sourc	42.41	4	10684	1231.5	3382	75	189	9
CCR6	CCR6	NSG0000011248	1235	protein_coding	Human	6	167.111807	or 6 [Source:HGN	45.54	4	27889	1125	2980	96	1687.5	3.5
SKAP2	SKAP2	NSG0000000502	8935	protein_coding	Human	7	26.667068	otein 2 [Source:H	36.94	9	328171	1080	581	112	1298	7
HOXA1	HOXA1	NSG0000010599	3198	protein_coding	Human	7	27.092993	rce:HGNC Symbo	46.78	2	3007	414	2016	85	1446	2.5
JAZF1	JAZF1	NSG0000015381	221895	protein_coding	Human	7	27.830573	urce:HGNC Symb	41.44	9	350222	243	581.5	44.5	182.5	5
IKZF1	IKZF1	NSG0000018581	10320	protein_coding	Human	7	50.304068	1 [Source:HGNC	43.45	22	101033	885	2647.5	22	3204	5
GTF2IRD1	GTF2IRD1	NSG0000000670	9569	protein_coding	Human	7	74.45379	ing 1 [Source:HG	52.72	7	148815	2880	3315	105.5	170.5	26
NCF1	NCF1	NSG0000015851	653361	protein_coding	Human	7	74.773962	1 [Source:HGNC	54.16	11	15414	437	946	69	151	6
CDK6	CDK6	NSG0000010581	1021	protein_coding	Human	7	92.604921	6 [Source:HGNC	38.19	5	231652	981	11612	230.5	10218	2
SPDYE3	SPDYE3	NSG0000021430	441272	protein_coding	Human	7	100.307702	ly member E3 [So	50.81	2	14494	1650	3185	183	674.5	8
IRF5	IRF5	NSG0000012860	3663	protein_coding	Human	7	128.937457	5 [Source:HGNC	56.44	18	12581	444	1652	37	257	5.5
BLK	BLK	NSG0000013657	640	protein_coding	Human	8	11.486894	sine kinase [Sour	46.62	7	77705	1305	2088	51	489	4
TPD52	TPD52	NSG0000007655	7163	protein_coding	Human	8	80.034745	urce:HGNC Symb	41.15	29	196487	336	785	120	87	5
GRHL2	GRHL2	NSG0000008330	79977	protein_coding	Human	8	101.492439	ctor 2 [Source:HG	40.92	6	177287	1830	2140	91	1605.5	4
CCN4	CCN4	NSG0000010441	8840	protein_coding	Human	8	133.191039	actor 4 [Source:H	48.12	5	40651	468	1205	75	269.5	3
PLGRKT	PLGRKT	NSG0000010702	55848	protein_coding	Human	9	5.357971	inal lysine [Source	39.9	4	79954	444	981	125	267	4.5
CCL21	CCL21	NSG0000013707	6366	protein_coding	Human	9	34.709005	21 [Source:HGNC	59.1	2	1131	390	731.5	35.5	304	3.5
CDK5RAP2	CDK5RAP2	NSG0000013686	55755	protein_coding	Human	9	120.388869	l protein 2 [Source	43.21	19	191301	1383	5826	85	130	6
IL2RA	IL2RA	NSG0000013446	3559	protein_coding	Human	10	6.010689	alpha [Source:HG	44.48	6	51681	792	1562	157	800	5.5
PRKCQ	PRKCQ	NSG0000006567	5588	protein_coding	Human	10	6.427143	Source:HGNC Sy	43.1	4	153158	1932	3224	90	1063	17
GATA3	GATA3	NSG0000010748	2625	protein_coding	Human	10	8.045378	[Source:HGNC Sy	51.55	5	29820	1332	2650	285.5	1020	3
ZNF438	ZNF438	NSG0000018362	220929	protein_coding	Human	10	30.820207	Source:HGNC Sy	39.39	9	211730	2457	3164	80	322	8
WDFY4	WDFY4	NSG0000012881	57705	protein_coding	Human	10	48.684876	[Source:HGNC Sy	45.39	6	298080	1965	2193	70.5	193	11.5
ARID5B	ARID5B	NSG0000015034	84159	protein_coding	Human	10	61.901684	5B [Source:HGNC	40.32	5	195260	3190.5	3141	17.5	2280	7
RTKN2	RTKN2	NSG0000018201	219790	protein_coding	Human	10	62.183035	e:HGNC Symbol;A	35.19	3	85809	927	2383	168	1486	9
ASCC1	ASCC1	NSG0000013830	51008	protein_coding	Human	10	72.096032	ex subunit 1 [Sou	41.33	26	121102	457	1501	68	121.5	6
SFTPD	SFTPD	NSG0000013366	6441	protein_coding	Human	10	79.937467	ource:HGNC Sym	44.62	4	45147	1128	1283	3	111	5.5
TSPAN32	TSPAN32	NSG0000006420	10077	protein_coding	Human	11	2.301997	rce:HGNC Symbo	63.03	17	16207	777	1202	136	179	8
SWAP70	SWAP70	NSG0000013378	23075	protein_coding	Human	11	9.664077	SWAP70 [Source:	39.97	7	88916	450	977	102	161	7
FADS2	FADS2	NSG0000013482	9415	protein_coding	Human	11	61.79298	[Source:HGNC Sy	51.23	15	74374	736	693.5	68.5	348.5	5
FADS1	FADS1	NSG0000014948	3992	protein_coding	Human	11	61.799627	[Source:HGNC Sy	50.81	21	29691	459	666.5	91	164.5	5
FADS3	FADS3	NSG0000022196	3995	protein_coding	Human	11	61.873519	[Source:HGNC Sy	57.37	13	18532	689	841	96	182.5	8
BAD	BAD	NSG0000000233	572	protein_coding	Human	11	64.26983	ll death [Source:H	53.92	7	14874	492	631	34	81	3
TPCN2	TPCN2	NSG0000016234	219931	protein_coding	Human	11	69.048932	2 [Source:HGNC	56.48	8	87384	1755	3824	63	137	19
CEP57	CEP57	NSG0000016603	9702	protein_coding	Human	11	95.789965	Source:HGNC Sy	37.69	16	42728	249	1739	177	121	6
CUL5	CUL5	NSG0000016626	8065	protein_coding	Human	11	108.008898	HGNC Symbol;A	39.36	6	98863	1365.5	1109	428	166	4.5
DDX6	DDX6	NSG0000011036	1656	protein_coding	Human	11	118.747763	Source:HGNC Sy	41.56	8	43401	1452	3628	179.5	1061.5	6.5
CXCR5	CXCR5	NSG0000016068	643	protein_coding	Human	11	118.883892	tor 5 [Source:HG	54.2	1	13895	1119	4293	49	3123	2
SCAF11	SCAF11	NSG0000013921	9169	protein_coding	Human	12	45.919131	tor 11 [Source:HG	36.52	15	72989	457	1931	179.5	745	4
CDK2	CDK2	NSG0000012337	1017	protein_coding	Human	12	55.966781	2 [Source:HGNC	47.7	12	6008	717	1546	178	274	6
TBX3	TBX3	NSG0000013511	6926	protein_coding	Human	12	114.670255	3 [Source:HGNC	47.3	4	13920	2202	4208	681.5	1585	4.5
FLT3	FLT3	NSG0000012202	2322	protein_coding	Human	13	28.003274	nase 3 [Source:H	43.22	3	97318	2220	3634	73	118	24
COG6	COG6	NSG0000013310	57511	protein_coding	Human	13	39.655627	mplex 6 [Source:H	35.35	12	136038	228	3449	64	93	4.5
AKAP11	AKAP11	NSG0000002351	11215	protein_coding	Human	13	42.272152	11 [Source:HGN	36.45	1	51109	5706	9915	49	4032	13
CLYBL	CLYBL	NSG0000012524	171425	protein_coding	Human	13	99.606669	ource:HGNC Sym	43.21	7	290465	733.5	919.5	15.5	165	4
PRKCH	PRKCH	NSG0000002707	5583	protein_coding	Human	14	61.187559	Source:HGNC Sym	42.48	28	363417	285	548.5	63	1144	4
RAD51B	RAD51B	NSG0000018218	5890	protein_coding	Human	14	67.819779	ource:HGNC Sym	40.99	24	910439	840	1536	23	136	5
ZFP36L1	ZFP36L1	NSG0000018565	677	protein_coding	Human	14	68.78766	e 1 [Source:HGN	53.26	6	8593	623	1872	83.5	204	2
SLC8A3	SLC8A3	NSG0000010067	6547	protein_coding	Human	14	70.044215	r A3 [Source:HGN	43.55	10	144855	1863	5133	72.5	1708.5	6.5

BATF	BATF	NSG0000015612	10538	protein_coding	Human	14	75.522455	ription factor [Sou	44.51	3	24538	300	617	167.5	167.5	3
PLD4	PLD4	NSG0000016642	122618	protein_coding	Human	14	104.924713	er 4 [Source:HGN	61.65	7	13048	1444.5	1905	139	271.5	7
RASGRP1	RASGRP1	NSG0000017257	10125	protein_coding	Human	15	38.488103	n 1 [Source:HGN	41.82	13	77472	1446	1910	108	120	15
TLE3	TLE3	NSG0000014033	7090	protein_coding	Human	15	70.04779	corepressor [Sour	52.15	25	50386	1882.5	3647	374	247	19
PSTPIP1	PSTPIP1	NSG0000014036	9051	protein_coding	Human	15	76.993359	eracting protein 1	56.5	18	44116	411	1678	164.5	98	9.5
IQGAP1	IQGAP1	NSG0000014057	8826	protein_coding	Human	15	90.388242	ng protein 1 [Sour	40.76	18	113997	3576	2313	87	135	3
TXNDC11	TXNDC11	NSG0000015306	51061	protein_coding	Human	16	11.679083	j 11 [Source:HGN	43.53	9	63774	444	863	116.5	111	5
PRKCB	PRKCB	NSG0000016650	5579	protein_coding	Human	16	23.835983	Source:HGNC Sy	43.77	12	384628	391	569	183	497	4
ZNF689	ZNF689	NSG0000015685	115509	protein_coding	Human	16	30.602558	Source:HGNC Sy	48.18	6	21454	1503	1309.5	347	1705	3
IRF8	IRF8	NSG0000014096	3394	protein_coding	Human	16	85.899162	8 [Source:HGNC	49.68	11	23444	661	946	49	904	4
C1QBP	C1QBP	NSG0000010856	708	protein_coding	Human	17	5.432777	otein [Source:HGN	46.67	7	16053	537	901	79	225	3
KSR1	KSR1	NSG0000014106	8844	protein_coding	Human	17	27.45647	1 [Source:HGNC	49.31	20	169968	2286	2220	140	177.5	4.5
MED1	MED1	NSG0000012568	5469	protein_coding	Human	17	39.404285	1 [Source:HGNC	45.01	5	46987	294	4584	207	100	16
GSDMB	GSDMB	NSG0000007360	55876	protein_coding	Human	17	39.904595	ce:HGNC Symbol	48.85	15	15259	1185	1277	56	145	8
DLGAP1	DLGAP1	NSG0000017057	9229	protein_coding	Human	18	3.496032	[Source:HGNC S	39.67	22	959275	2052	3525	75.5	3152	8
PTPN2	PTPN2	NSG0000017535	5771	protein_coding	Human	18	12.785478	ceptor type 2 [Sou	42.19	21	144165	501	1326	93	157	5
TNFRSF11A	TNFRSF11A	NSG0000014165	8792	protein_coding	Human	18	62.325287	er 11a [Source:H	44.92	7	66001	900	3756	65	2812.5	8
CD226	CD226	NSG0000015063	10666	protein_coding	Human	18	69.831158	urce:HGNC Symb	39.64	8	130645	546	1009	125	203	5
ARID3A	ARID3A	NSG0000011601	1820	protein_coding	Human	19	0.925781	3A [Source:HGNC	58.94	7	50158	1399	572.5	145	140	2
TYK2	TYK2	NSG0000010539	7297	protein_coding	Human	19	10.350529	urce:HGNC Symb	54.33	21	30043	429	720	101	127	4
ILF3	ILF3	NSG0000012935	3609	protein_coding	Human	19	10.654261	ctor 3 [Source:H	50.9	24	38156	422	791	89	111	7
HAPLN4	HAPLN4	NSG0000018766	404037	protein_coding	Human	19	19.254756	protein 4 [Source:	58.5	2	8048	1209	4342	71	3060	4
KMT2B	KMT2B	NSG0000027233	9757	protein_coding	Human	19	35.717973	3 [Source:HGNC	59.82	9	20905	347	5791	30	115	6
TGFB1	TGFB1	NSG0000010532	7040	protein_coding	Human	19	41.301587	ta 1 [Source:HGN	53.14	5	52335	1113	1250	877	162	5
SIGLEC6	SIGLEC6	NSG0000010549	946	protein_coding	Human	19	51.517819	n 6 [Source:HGN	46.97	9	14037	1188	1746	149.5	484.5	6
CD40	CD40	NSG0000010101	958	protein_coding	Human	20	46.118278	rce:HGNC Symb	49.19	7	11585	479	1522	29	192	8
RUNX1	RUNX1	NSG0000015921	861	protein_coding	Human	21	34.787801	tor 1 [Source:HGN	41.54	18	1216866	420	1590	111.5	388	4.5
UBASH3A	UBASH3A	NSG0000016018	53347	protein_coding	Human	21	42.403447	containing A [Sou	49.32	9	44237	1356	2005	33	140.5	12
AIRE	AIRE	NSG0000016022	326	protein_coding	Human	21	44.285838	[Source:HGNC S	63.87	5	12810	1638	2690	130	920	12
UBE2L3	UBE2L3	NSG0000018565	7332	protein_coding	Human	22	21.549447	E2 L3 [Source:HG	49.73	4	74587	465	2932	30	2364	4
YDJC	YDJC	NSG0000016117	150223	protein_coding	Human	22	21.628089	se homolog [Sour	67.56	7	1975	450	1410	20	328	4
CSF2RB	CSF2RB	NSG0000010036	1439	protein_coding	Human	22	36.913628	subunit beta [Sour	51.95	4	26811	2670	4087	95.5	1199	13.5
SYNGR1	SYNGR1	NSG0000010032	9145	protein_coding	Human	22	39.349925	rce:HGNC Symb	55.76	7	35650	510	1111.5	23.5	229	4
SMC1B	SMC1B	NSG0000007793	27127	protein_coding	Human	22	45.344063	somes 1B [Source	39.89	2	69556	3597	3934.5	41	294.5	24
TLR8	TLR8	NSG0000010191	51311	protein_coding	Human	X	12.90662	urce:HGNC Symb	41.32	2	16549	3180	3367	79	518.5	2.5
VSIG4	VSIG4	NSG0000015565	11326	protein_coding	Human	X	66.021738	ontaining 4 [Sourc	40.15	5	18387	918	1941	97.5	419.5	7
GPR174	GPR174	NSG0000014713	84636	protein_coding	Human	X	79.144688	74 [Source:HGN	36.53	1	30630	1002	5494	529	3308	3
IRAK1	IRAK1	NSG0000018421	3654	protein_coding	Human	X	154.010506	kinase 1 [Source:	61.62	13	9396	1020	2242.5	84	203.5	7
C20ORF181	C20orf181	NSG0000027961	NA	TEC	Human	20	63.843436	me 181 [Source:H	63.13	1	479	NA	480	NA	NA	1
CASTOR3	CASTOR3	NSG0000023952	352954	l_unprocessed_ps	Human	7	100.200653	3 [Source:HGNC S	51.53	12	71565	NA	847	NA	NA	5
A20	NA	Not mapped	NA	NA	NA	NA	NA	NA	NA	NA	NA	NA	NA	NA	NA	NA
ACPA	NA	Not mapped	NA	NA	NA	NA	NA	NA	NA	NA	NA	NA	NA	NA	NA	NA
AGR3-AHR	NA	Not mapped	NA	NA	NA	NA	NA	NA	NA	NA	NA	NA	NA	NA	NA	NA
CSE	NA	Not mapped	NA	NA	NA	NA	NA	NA	NA	NA	NA	NA	NA	NA	NA	NA
DR4	NA	Not mapped	NA	NA	NA	NA	NA	NA	NA	NA	NA	NA	NA	NA	NA	NA
FCGR	NA	Not mapped	NA	NA	NA	NA	NA	NA	NA	NA	NA	NA	NA	NA	NA	NA
FLI1-ETS1	NA	Not mapped	NA	NA	NA	NA	NA	NA	NA	NA	NA	NA	NA	NA	NA	NA
FLT-1	NA	Not mapped	NA	NA	NA	NA	NA	NA	NA	NA	NA	NA	NA	NA	NA	NA
GNG4	NA	Not mapped	NA	NA	NA	NA	NA	NA	NA	NA	NA	NA	NA	NA	NA	NA
HLA-DRB1	NA	Not mapped	NA	NA	NA	NA	NA	NA	NA	NA	NA	NA	NA	NA	NA	NA
IFI144	NA	Not mapped	NA	NA	NA	NA	NA	NA	NA	NA	NA	NA	NA	NA	NA	NA
IFNGR2	NA	Not mapped	NA	NA	NA	NA	NA	NA	NA	NA	NA	NA	NA	NA	NA	NA
IL-10	NA	Not mapped	NA	NA	NA	NA	NA	NA	NA	NA	NA	NA	NA	NA	NA	NA
IL-17A	NA	Not mapped	NA	NA	NA	NA	NA	NA	NA	NA	NA	NA	NA	NA	NA	NA
IL-1BETA	NA	Not mapped	NA	NA	NA	NA	NA	NA	NA	NA	NA	NA	NA	NA	NA	NA
IL-6	NA	Not mapped	NA	NA	NA	NA	NA	NA	NA	NA	NA	NA	NA	NA	NA	NA
LMP2	NA	Not mapped	NA	NA	NA	NA	NA	NA	NA	NA	NA	NA	NA	NA	NA	NA
LOC105369698	NA	Not mapped	NA	NA	NA	NA	NA	NA	NA	NA	NA	NA	NA	NA	NA	NA
LOC105373023	NA	Not mapped	NA	NA	NA	NA	NA	NA	NA	NA	NA	NA	NA	NA	NA	NA

LOC107984408	NA	Not mapped	NA	NA	NA	NA	NA	NA	NA	NA	NA	NA	NA	NA	NA	NA
MIC-1	NA	Not mapped	NA	NA	NA	NA	NA	NA	NA	NA	NA	NA	NA	NA	NA	NA
MMEL1	NA	Not mapped	NA	NA	NA	NA	NA	NA	NA	NA	NA	NA	NA	NA	NA	NA
NFKB	NA	Not mapped	NA	NA	NA	NA	NA	NA	NA	NA	NA	NA	NA	NA	NA	NA
NRAMP1	NA	Not mapped	NA	NA	NA	NA	NA	NA	NA	NA	NA	NA	NA	NA	NA	NA
OS9-AGAP2	NA	Not mapped	NA	NA	NA	NA	NA	NA	NA	NA	NA	NA	NA	NA	NA	NA
PADI4	NA	Not mapped	NA	NA	NA	NA	NA	NA	NA	NA	NA	NA	NA	NA	NA	NA
PDE2A-ARAP1	NA	Not mapped	NA	NA	NA	NA	NA	NA	NA	NA	NA	NA	NA	NA	NA	NA
PLCL1-LINC01923	NA	Not mapped	NA	NA	NA	NA	NA	NA	NA	NA	NA	NA	NA	NA	NA	NA
PLCL2	NA	Not mapped	NA	NA	NA	NA	NA	NA	NA	NA	NA	NA	NA	NA	NA	NA
PRDM1-ATG5	NA	Not mapped	NA	NA	NA	NA	NA	NA	NA	NA	NA	NA	NA	NA	NA	NA
PSORS1C1	NA	Not mapped	NA	NA	NA	NA	NA	NA	NA	NA	NA	NA	NA	NA	NA	NA
PTPRC	NA	Not mapped	NA	NA	NA	NA	NA	NA	NA	NA	NA	NA	NA	NA	NA	NA
SH2B3-PTPN11	NA	Not mapped	NA	NA	NA	NA	NA	NA	NA	NA	NA	NA	NA	NA	NA	NA
TAMM41-SYN2	NA	Not mapped	NA	NA	NA	NA	NA	NA	NA	NA	NA	NA	NA	NA	NA	NA
TGF-BETA1	NA	Not mapped	NA	NA	NA	NA	NA	NA	NA	NA	NA	NA	NA	NA	NA	NA
TL1A	NA	Not mapped	NA	NA	NA	NA	NA	NA	NA	NA	NA	NA	NA	NA	NA	NA
TNFALPHA	NA	Not mapped	NA	NA	NA	NA	NA	NA	NA	NA	NA	NA	NA	NA	NA	NA
TRAF1-C5	NA	Not mapped	NA	NA	NA	NA	NA	NA	NA	NA	NA	NA	NA	NA	NA	NA
