# Supplementary material for: Integrative molecular network analysis of genetic risk factors to infer biomarkers and therapeutic targets for rheumatoid arthritis
Source: PLoS One. 2025 Aug 21;20(8):e0329101. doi: 10.1371/journal.pone.0329101 (PMC12370121; doi:10.1371/journal.pone.0329101)
Supplement: S4 Table — (PDF) [file pone.0329101.s004.pdf]

Network #	term ID	term description	observed gene count	background gene count	strength	false discovery rate	Proteins	Lable
Network 1	WP254	Apoptosis	21	84	1.55E+00	8.75E-23	TNFSF10,MDM2,RIPK1,TP53,TNFRSF10B,CASP10,FADD,CASP3,CFLAR,TRADD,CASP8,JUN,TNFRSF1B,TNF,PIK3R1,CASP1,AKT1,IRF3,I	wikipathways
Network 1	WP1772	Apoptosis modulation and signaling	19	90	1.48E+00	1.48E-19	,TNFRSF10A,TNFSF10,RIPK1,TP53,TNFRSF10B,CASP10,FADD,CASP3,CFLAR,IL1R2,TRADD,CASP8,JUN,TNFRSF1B,IL1R1,MYD88,CAS	wikipathways
Network 1	HSA-1280215	Cytokine Signaling in Immune system	65	681	1.13E+00	4.24E-52	2,IL10RA,IL23A,IFNG,IL4,PLCG1,IL1RN,IRAK3,IL12RB2,IL1B,STAT3,IRF8,TP53,RPS27A,IL10RB,CCR2,IL17RC,CCR1,CXCL8,CASP3,STAT2<br>MMP9,TNFSF13B,TNFRSF1B,ISG15,IL6ST,JAK2,RSAD2,STAT4,CXCL1,YWHAZ,IRF9,MX1,IL6,FOXO3,IL1R1,TNF,MYD88,IL10,PIK3R1,TYK2,	REACTOME
Network 1	hsa04060	Cytokine-cytokine receptor interaction	36	282	1.26E+00	9.31E-32	F10,GDF15,IL1RN,IL12RB2,IL1B,TNFRSF10B,IL10RB,CCR2,IL17RC,CCR1,CXCL8,IL17RA,IL23R,IL1R2,IL17A,TGFBR2,IL6R,TGFBR1,TNFS	KEGG
Network 1	WP4655	Cytosolic DNA-sensing pathway	17	73	1.52E+00	4.41E-18	PYCARD,RIPK1,IL1B,CASP10,FADD,TBK1,TMEM173,TRADD,CASP8,AIM2,MB21D1,ISG15,IL6,MAVS,CASP1,IRF3,IKBKG	wikipathways
Network 1	HSA-168928	DDX58/IFIH1-mediated induction of interferon-alpha/beta	15	76	1.45E+00	1.49E-14	RIPK1,EP300,RPS27A,CASP10,FADD,TBK1,CASP8,ISG15,TAX1BP1,MAVS,TRAF6,UBC,IRF3,TNFAIP3,IKBKG	REACTOME
Network 1	HSA-73887	Death Receptor Signalling	20	140	1.31E+00	3.30E-17	RSF10A,TNFSF10,RIPK1,RPS27A,TNFRSF10B,CASP10,FADD,CASP3,CFLAR,TRADD,CASP8,HDAC1,TAX1BP1,TNF,MYD88,TRAF6,UBC,	REACTOME
Network 1	HSA-1643685	Disease	47	1548	6.40E-01	5.93E-16	S27A,FADD,STAT2,TBK1,NLRP3,PTPN11,TRADD,JAK1,CTNNB1,CASP8,VCP,TGFBR2,STAT1,IL6R,JUN,HDAC1,TGFBR1,ISG15,JAK2,IL6,F	REACTOME
Network 1	hsa05169	Epstein-Barr virus infection	29	193	1.33E+00	2.73E-27	,TP53,FADD,CASP3,STAT2,TBK1,TRADD,JAK1,CASP8,STAT1,JUN,HDAC1,ISG15,IRF9,IL6,TNF,MYD88,MAVS,PIK3R1,TYK2,TRAF6,AKT1,I	KEGG
Network 1	hsa05161	Hepatitis B	32	159	1.46E+00	2.08E-33	10,FADD,CXCL8,CASP3,STAT2,TBK1,JAK1,CASP8,TGFBR2,STAT1,JUN,MMP9,TGFBR1,JAK2,STAT4,YWHAZ,IL6,TNF,MYD88,MAVS,PIK3F	KEGG
Network 1	WP4666	Hepatitis B infection	30	151	1.45E+00	2.95E-30	DD,CXCL8,CASP3,STAT2,TBK1,JAK1,CASP8,TGFBR2,STAT1,JUN,MMP9,TGFBR1,JAK2,STAT4,YWHAZ,IL6,TNF,MYD88,MAVS,PIK3R1,TYK	wikipathways
Network 1	hsa05160	Hepatitis C	30	156	1.44E+00	5.78E-31	DK2,TP53,FADD,CASP3,CFLAR,STAT2,TBK1,TRADD,JAK1,CTNNB1,CASP8,STAT1,IFIT1,RSAD2,YWHAZ,IRF9,MX1,TNF,MAVS,PIK3R1,TYK	KEGG
Network 1	WP3646	Hepatitis C and hepatocellular carcinoma	15	49	1.64E+00	1.98E-17	TGFB1,STAT3,TP53,CXCL8,CASP3,PTPN11,JAK1,IL6R,JUN,TGFBR1,IL6,BRCA1,AKT1,VEGFA,MYC	wikipathways
Network 1	hsa05163	Human cytomegalovirus infection	31	218	1.31E+00	1.47E-28	,ITGAV,IL1B,STAT3,TP53,IL10RB,CCR1,FADD,CXCL8,CASP3,TBK1,TMEM173,TRADD,JAK1,CTNNB1,CASP8,IL6R,MB21D1,IL6,IL1R1,TNF,	KEGG
Network 1	HSA-168256	Immune System	90	1956	8.20E-01	5.36E-52	K1,ITGAV,IRAK3,FAF2,IL12RB2,EP300,IL1B,STAT3,IRF8,TP53,RPS27A,CASP10,STOM,IL10RB,CCR2,IL17RC,CCR1,FADD,CXCL8,CASP3,T<br>,JUN,IFIT1,IFIT3,MMP9,TNFSF13B,TNFRSF1B,ISG15,IL6ST,JAK2,RSAD2,STAT4,CXCL1,YWHAZ,TAX1BP1,IRF9,MX1,NLRC4,IL6,FOXO3,IL<br>TYK2,CASP1,TRAF6,UBC,AKT1,IL12RB1,IRF3,NLRP1,VEGFA,MYC,TNFAIP3,IKBKG	REACTOME
Network 1	HSA-168249	Innate Immune System	45	1025	8.00E-01	4.73E-21	CCR2,FADD,TLR8,TNIP2,TBK1,TMEM173,NLRP3,PTPN11,CTNNB1,CASP8,VCP,AIM2,IFI16,MB21D1,JUN,MMP9,TNFRSF1B,ISG15,CXCL1,	REACTOME
Network 1	WP1984	Integrated breast cancer pathway	20	151	1.28E+00	3.09E-17	RT1,MDM2,EP300,CDK2,TP53,FADD,CASP3,TRADD,JAK1,CTNNB1,CASP8,TGFBR2,STAT1,JUN,HDAC1,TGFBR1,BRCA1,AKT1,VEGFA,MY	wikipathways
Network 1	HSA-6783783	Interleukin-10 signaling	19	45	1.78E+00	8.01E-24	TNFRSF1A,CCL2,IL10RA,IL1RN,IL1B,STAT3,IL10RB,CCR2,CCR1,CXCL8,IL1R2,JAK1,TNFRSF1B,CXCL1,IL6,IL1R1,TNF,IL10,TYK2	REACTOME
Network 1	HSA-6785807	Interleukin-4 and Interleukin-13 signaling	25	107	1.52E+00	1.98E-26	CL2,IL23A,IL4,IL1B,STAT3,TP53,CXCL8,IL23R,JAK1,IL17A,STAT1,IL6R,MMP9,TNFRSF1B,JAK2,IL6,FOXO3,TNF,IL10,PIK3R1,TYK2,AKT1,VE	REACTOME
Network 1	hsa05167	Kaposi sarcoma-associated herpesvirus infection	31	187	1.37E+00	2.39E-30	53,RPS27A,CCR1,FADD,CXCL8,CASP3,STAT2,TBK1,TRADD,JAK1,CTNNB1,CASP8,STAT1,JUN,IL6ST,JAK2,CXCL1,IRF9,IL6,PIK3R1,TYK2,	KEGG
Network 1	hsa05162	Measles	25	138	1.41E+00	3.28E-25	CDK2,TP53,FADD,CASP3,STAT2,TBK1,TRADD,JAK1,CASP8,STAT1,JUN,IRF9,MX1,IL6,MYD88,MAVS,PIK3R1,TYK2,TRAF6,AKT1,IRF3,TNF	KEGG
Network 1	WP5083	Neuroinflammation and glutamatergic signaling	23	140	1.37E+00	1.50E-21	,TGFBR3,TGFB1,IL10RA,IFNG,IL4,IL1B,STAT3,IL10RB,IL1R2,JAK1,TGFBR2,STAT1,IL6R,TGFBR1,TNFRSF1B,IL6ST,IL6,IL1R1,TNF,IL10,SL	wikipathways
Network 1	hsa04621	NOD-like receptor signaling pathway	33	174	1.43E+00	1.35E-33	L8,NLRP6,STAT2,TBK1,TMEM173,NLRP3,JAK1,CASP8,STAT1,AIM2,JUN,TP53BP1,CASP5,CXCL1,IRF9,NLRC4,IL6,TNF,MYD88,MAVS,TYK2	KEGG
Network 1	hsa05200	Pathways in cancer	44	517	1.08E+00	2.22E-32	3,FADD,CXCL8,CASP3,VEGFB,STAT2,IL23R,JAK1,CTNNB1,CASP8,TGFBR2,STAT1,IL6R,JUN,MMP9,HDAC1,TGFBR1,ZBTB17,IL6ST,JAK2	KEGG
Network 1	WP1449	Regulation of toll-like receptor signaling pathway	19	139	1.29	1.33E-16	RIPK1,IRAK3,IL1B,FADD,CXCL8,TLR8,TBK1,CASP8,STAT1,JUN,IL6,TNF,MYD88,PIK3R1,TRAF6,AKT1,IRF3,TNFAIP3,IKBKG	wikipathways
Network 1	WP5039	SARS-CoV-2 innate immunity evasion and cell-specific immune response	21	66	1.66	1.73E-24	TGFB1,CCL2,RIPK1,EP300,FADD,CXCL8,STAT2,TBK1,TRADD,JAK1,CASP8,STAT1,IL6R,CXCL1,MX1,IL6,TNF,MAVS,IL10,TRAF6,IRF3	wikipathways
Network 1	HSA-162582	Signal Transduction	62	2741	0.51	4.64E-16	NFSF10,PLCG1,MDM2,RIPK1,ITGAV,EP300,KDR,STAT3,NRP1,CDK2,TP53,RPS27A,TNFRSF10B,FLT1,CASP10,CCR2,CCR1,FADD,CXCL8,<br>,IL6R,JUN,MMP9,HDAC1,TGFBR1,IL6ST,JAK2,ACVRL1,CXCL1,YWHAZ,TAX1BP1,MDK,IL6,FOXO3,TNF,MYD88,PIK3R1,TYK2,TRAF6,UBC,	REACTOME
Network 1	HSA-449147	Signaling by Interleukins	55	440	1.25	4.67E-49	CCR1,CXCL8,CASP3,STAT2,IL17RA,TNIP2,IL23R,TBK1,IL1R2,PTPN11,JAK1,IL17A,STAT1,IL6R,JUN,MMP9,TNFRSF1B,IL6ST,JAK2,STAT4,	REACTOME
Network 1	WP5098	T-cell activation SARS-CoV-2	19	86	1.5	8.32E-20	IL23A,IFNG,IL4,PLCG1,IL12RB2,TP53,IL23R,IL17A,JUN,JAK2,STAT4,FOXO3,TNF,MYD88,PIK3R1,TYK2,AKT1,IL12RB1,IKBKG	wikipathways
Network 2	WP2805	exRNA mechanism of action and biogenesis	2	6	2.54E+00	0.0084	DGCR8,XPO4	wikipathways
Network 2	HSA-6803529	FGFR2 alternative splicing	3	26	2.07	0.0012	HNRNPM,HNRNPA1,PTBP0	REACTOME
Network 2	WP2338	miRNA biogenesis	2	6	2.54E+00	0.0084	DGCR8,XPO4	wikipathways
Network 2	WP411	mRNA processing	12	125	2.00E+00	2.75E-19	HNRNPL,RBM39,FUS,NONO,HNRNPD,HNRNPM,HNRNPA1,HNRNPA2B1,PTBP1,PCBP2,DHX9,HNRNPC	wikipathways
Network 2	HSA-72163	mRNA Splicing - Major Pathway	12	178	1.84E+00	5.05E-17	HNRNPL,FUS,PCBP1,HNRNPD,HNRNPM,HNRNPA1,HNRNPA2B1,PTBP1,PCBP2,DHX9,HNRNPA3,HNRNPC	REACTOME
Network 2	hsa03040	Spliceosome	6	132	1.67	9.28E-07	FUS,PCBP1,HNRNPM,HNRNPA1,HNRNPA3,HNRNPC	KEGG
Network 3	HSA-5620912	Anchoring of the basal body to the plasma membrane	5	97	1.96E+00	2.84E-07	CDK5RAP2,AKAP9,PCNT,CEP152,CENPJ	REACTOME
Network 3	HSA-8854518	AURKA Activation by TPX2	5	72	2.09E+00	9.47E-08	CDK5RAP2,AKAP9,PCNT,CEP152,CENPJ	REACTOME
Network 3	HSA-380259	Loss of Nlp from mitotic centrosomes	5	69	2.11E+00	9.47E-08	CDK5RAP2,AKAP9,PCNT,CEP152,CENPJ	REACTOME
Network 3	HSA-380270	Recruitment of mitotic centrosome proteins and complexes	7	80	2.19	1.89E-11	MZT2A,CDK5RAP2,AKAP9,PCNT,CEP152,CENPJ,TUBGCP1	REACTOME
Network 3	HSA-380320	Recruitment of NuMA to mitotic centrosomes	7	93	2.13	1.89E-11	MZT2A,CDK5RAP2,AKAP9,PCNT,CEP152,CENPJ,TUBGCP1	REACTOME
Network 3	HSA-2565942	Regulation of PLK1 Activity at G2/M Transition	5	87	2.01	1.83E-07	CDK5RAP2,AKAP9,PCNT,CEP152,CENPJ	REACTOME
Network 4	HSA-1640170	Cell Cycle	10	647	1.44E+00	1.65E-11	STAG2,ESPL1,NIPBL,RAD21,SMC1B,SGOL2,SMC3,STAG1,STAG3,REC7	REACTOME
Network 4	hsa04110	Cell cycle	6	120	1.95E+00	9.53E-09	STAG2,ESPL1,RAD21,SMC1B,SMC3,STAG0	KEGG
Network 4	WP179	Cell cycle	6	119	1.95E+00	1.83E-08	STAG2,ESPL1,RAD21,SMC1B,SMC3,STAG0	wikipathways
Network 4	HSA-2470946	Cohesin Loading onto Chromatin	5	10	2.95E+00	3.14E-11	STAG2,NIPBL,RAD21,SMC3,STAG0	REACTOME
Network 4	HSA-2468052	Establishment of Sister Chromatid Cohesion	4	11	2.81E+00	2.28E-08	STAG2,RAD21,SMC3,STAG0	REACTOME
Network 4	HSA-9018519	Estrogen-dependent gene expression	4	119	1.78E+00	6.42E-05	STAG2,RAD21,SMC3,STAG0	REACTOME
Network 4	HSA-68886	M Phase	7	376	1.52E+00	8.70E-08	STAG2,ESPL1,NIPBL,RAD21,SGOL2,SMC3,STAG0	REACTOME
Network 4	HSA-1221632	Meiotic synapsis	7	60	2.32E+00	2.82E-12	STAG2,RAD21,SMC1B,SMC3,STAG1,STAG3,REC7	REACTOME
Network 4	hsa04114	Oocyte meiosis	5	120	1.87E+00	7.37E-07	ESPL1,SMC1B,SMC3,STAG3,REC7	KEGG
Network 4	WP3995	Prion disease pathway	2	32	2.05	0.036	RAD21,SMC2	wikipathways
Network 4	WP4240	Regulation of sister chromatid separation at the metaphase-anaphase transition	4	14	2.71	5.57E-08	ESPL1,RAD21,SMC3,STAG0	wikipathways
Network 4	HSA-2500257	Resolution of Sister Chromatid Cohesion	5	122	1.86	7.94E-07	STAG2,RAD21,SGOL2,SMC3,STAG0	REACTOME
Network 4	HSA-2467813	Separation of Sister Chromatids	6	184	1.76	8.70E-08	STAG2,ESPL1,RAD21,SGOL2,SMC3,STAG0	REACTOME
Network 4	HSA-3108214	SUMOylation of DNA damage response and repair proteins	4	75	1.98	1.15E-05	STAG2,RAD21,SMC3,STAG0	REACTOME
Network 5	HSA-1296041	Activation of G protein gated Potassium channels	7	29	2.63E+00	1.05E-15	GNB3,GNB4,GNG11,GNB5,GNB2,GNG4,GNB0	REACTOME
Network 5	HSA-991365	Activation of GABAB receptors	9	43	2.57E+00	5.38E-20	GNB3,GNB4,GNG11,GNB5,GNB2,GNAI1,GNAI3,GNG4,GNB0	REACTOME
Network 5	HSA-170670	Adenylate cyclase inhibitory pathway	2	14	2.41E+00	0.0011	GNAI1,GNAI2	REACTOME
Network 5	HSA-9660821	ADORA2B mediated anti-inflammatory cytokines production	9	132	2.08E+00	2.26E-16	GNB3,GNB4,GNG11,GNB5,GNB2,GNAI1,GNAI3,GNG4,GNB0	REACTOME
Network 5	HSA-418592	ADP signalling through P2Y purinoceptor 1	7	25	2.70E+00	4.58E-16	GNB3,GNB4,GNG11,GNB5,GNB2,GNG4,GNB0	REACTOME
Network 5	HSA-392170	ADP signalling through P2Y purinoceptor 12	9	22	2.86E+00	1.03E-21	GNB3,GNB4,GNG11,GNB5,GNB2,GNAI1,GNAI3,GNG4,GNB0	REACTOME
Network 5	HSA-400042	Adrenaline,noradrenaline inhibits insulin secretion	10	28	2.80E+00	4.96E-24	GNB3,GNB4,GNG11,GNB5,CACNA1C,GNB2,GNAI1,GNG4,CACNA2D2,GNB0	REACTOME
Network 5	hsa04261	Adrenergic signaling in cardiomyocytes	4	147	1.68E+00	1.90E-05	CACNA1C,GNAI1,GNAI3,CACNA2D1	KEGG
Network 5	hsa05034	Alcoholism	9	144	2.05E+00	1.42E-16	GNB3,GNB4,GNG11,GNB5,GNB2,GNAI1,GNAI3,GNG4,GNB0	KEGG
Network 5	hsa04371	Apelin signaling pathway	9	131	2.09E+00	6.88E-17	GNB3,GNB4,GNG11,GNB5,GNB2,GNAI1,GNAI3,GNG4,GNB0	KEGG
Network 5	hsa05412	Arrhythmogenic right ventricular cardiomyopathy	2	76	1.67E+00	0.0091	CACNA1C,CACNA2D1	KEGG
Network 5	WP2118	Arrhythmogenic right ventricular cardiomyopathy	2	74	1.68E+00	0.0387	CACNA1C,CACNA2D1	wikipathways
Network 5	hsa04360	Axon guidance	2	177	1.30E+00	0.0316	GNAI1,GNAI2	KEGG
Network 5	HSA-4086398	Ca2+ pathway	7	60	2.32E+00	6.27E-14	GNB3,GNB4,GNG11,GNB5,GNB2,GNG4,GNB0	REACTOME
Network 5	WP536	Calcium regulation in cardiac cells	10	148	2.08E+00	6.51E-18	GNB3,GNB4,GNG11,GNB5,CACNA1C,GNB2,GNAI1,GNAI3,GNG4,GNB0	wikipathways
Network 5	hsa04024	cAMP signaling pathway	3	208	1.41E+00	0.0026	CACNA1C,GNAI1,GNAI2	KEGG
Network 5	hsa04260	Cardiac muscle contraction	2	87	1.61E+00	0.0111	CACNA1C,CACNA2D1	KEGG
Network 5	hsa04022	cGMP-PKG signaling pathway	3	162	1.52E+00	0.0013	CACNA1C,GNAI1,GNAI2	KEGG
Network 5	hsa05142	Chagas disease	2	99	1.56E+00	0.0121	GNAI1,GNAI2	KEGG
Network 5	hsa04062	Chemokine signaling pathway	9	186	1.93E+00	1.21E-15	GNB3,GNB4,GNG11,GNB5,GNB2,GNAI1,GNAI3,GNG4,GNB0	KEGG
Network 5	WP3929	Chemokine signaling pathway	9	164	1.99E+00	4.89E-15	GNB3,GNB4,GNG11,GNB5,GNB2,GNAI1,GNAI3,GNG4,GNB0	wikipathways
Network 5	hsa04725	Cholinergic synapse	10	110	2.21E+00	5.25E-20	GNB3,GNB4,GNG11,GNB5,CACNA1C,GNB2,GNAI1,GNAI3,GNG4,GNB0	KEGG

Network #	term ID	term description	observed gene count	background gene count	strength	false discovery rate	Proteins	Lable
Network 5	hsa04713	Circadian entrainment	10	92	2.29E+00	1.83E-20	GNB3,GNB4,GNG11,GNB5,CACNA1C,GNB2,GNAI1,GNAI3,GNG4,GNB0	KEGG
Network 5	hsa05030	Cocaine addiction	2	49	1.86E+00	0.0047	GNAI1,GNAI2	KEGG
Network 5	HSA-6814122	Cooperation of PDCL (PhLP1) and TRiC/CCT in G-protein beta folding	9	42	2.58E+00	5.19E-20	GNB3,GNB4,GNG11,GNB5,GNB2,GNAI1,GNAI3,GNG4,GNB0	REACTOME
Network 5	WP2355	Corticotropin-releasing hormone signaling pathway	5	91	1.99E+00	1.12E-07	GNB3,GNB5,GNB2,GNAI1,GNB0	wikipathways
Network 5	hsa04934	Cushing syndrome	3	153	1.54E+00	0.0011	CACNA1C,GNAI1,GNAI2	KEGG
Network 5	hsa05414	Dilated cardiomyopathy	2	95	1.57E+00	0.0119	CACNA1C,CACNA2D1	KEGG
Network 5	hsa04728	Dopaminergic synapse	10	128	2.14E+00	1.33E-19	GNB3,GNB4,GNG11,GNB5,CACNA1C,GNB2,GNAI1,GNAI3,GNG4,GNB0	KEGG
Network 5	WP2197	Endothelin pathways	2	32	2.05E+00	0.009	GNB5,GNAI0	wikipathways
Network 5	hsa04915	Estrogen signaling pathway	2	133	1.43E+00	0.0186	GNAI1,GNAI2	KEGG
Network 5	HSA-9009391	Extra-nuclear estrogen signaling	9	74	2.34E+00	2.80E-18	GNB3,GNB4,GNG11,GNB5,GNB2,GNAI1,GNAI3,GNG4,GNB0	REACTOME
Network 5	WP3932	Focal adhesion: PI3K-Akt-mTOR-signaling pathway	6	302	1.55	5.29E-07	GNB3,GNB4,GNG11,GNB2,GNG4,GNB0	wikipathways
Network 5	HSA-416482	G alpha (12/13) signalling events	7	79	2.2	3.78E-13	GNB3,GNB4,GNG11,GNB5,GNB2,GNG4,GNB0	REACTOME
Network 5	HSA-416476	G alpha (q) signalling events	7	214	1.76	2.66E-10	GNB3,GNB4,GNG11,GNB5,GNB2,GNG4,GNB0	REACTOME
Network 5	HSA-418555	G alpha (s) signalling events	9	532	1.48	1.89E-11	GNB3,GNB4,GNG11,GNB5,GNB2,GNAI1,GNAI3,GNG4,GNB0	REACTOME
Network 5	HSA-418597	G alpha (z) signalling events	9	48	2.52	1.02E-19	GNB3,GNB4,GNG11,GNB5,GNB2,GNAI1,GNAI3,GNG4,GNB0	REACTOME
Network 5	HSA-8964315	G beta:gamma signalling through BTK	7	18	2.84	9.83E-17	GNB3,GNB4,GNG11,GNB5,GNB2,GNG4,GNB0	REACTOME
Network 5	HSA-8964616	G beta:gamma signalling through CDC42	7	20	2.79	1.61E-16	GNB3,GNB4,GNG11,GNB5,GNB2,GNG4,GNB0	REACTOME
Network 5	HSA-392451	G beta:gamma signalling through PI3Kgamma	7	25	2.7	4.58E-16	GNB3,GNB4,GNG11,GNB5,GNB2,GNG4,GNB0	REACTOME
Network 5	HSA-418217	G beta:gamma signalling through PLC beta	7	20	2.79	1.61E-16	GNB3,GNB4,GNG11,GNB5,GNB2,GNG4,GNB0	REACTOME
Network 5	WP35	G protein signaling pathways	8	91	2.19	1.18E-14	GNB3,GNG11,GNB5,GNB2,GNAI1,GNAI3,GNG4,GNB0	wikipathways
Network 5	HSA-202040	G-protein activation	9	28	2.76	4.25E-21	GNB3,GNB4,GNG11,GNB5,GNB2,GNAI1,GNAI3,GNG4,GNB0	REACTOME
Network 5	hsa04727	GABAergic synapse	10	86	2.32	1.83E-20	GNB3,GNB4,GNG11,GNB5,CACNA1C,GNB2,GNAI1,GNAI3,GNG4,GNB0	KEGG
Network 5	hsa04540	Gap junction	2	87	1.61	0.0111	GNAI1,GNAI2	KEGG
Network 5	hsa04971	Gastric acid secretion	2	73	1.69	0.009	GNAI1,GNAI2	KEGG
Network 5	HSA-163359	Glucagon signaling in metabolic regulation	6	33	2.51	8.67E-13	GNB3,GNB4,GNG11,GNB2,GNG4,GNB0	REACTOME
Network 5	HSA-381676	Glucagon-like Peptide-1 (GLP1) regulates insulin secretion	7	42	2.47	7.36E-15	GNB3,GNB4,GNG11,GNB5,GNB2,GNG4,GNB0	REACTOME
Network 5	HSA-420092	Glucagon-type ligand receptors	7	33	2.58	1.79E-15	GNB3,GNB4,GNG11,GNB5,GNB2,GNG4,GNB0	REACTOME
Network 5	hsa04724	Glutamatergic synapse	10	111	2.2	5.25E-20	GNB3,GNB4,GNG11,GNB5,CACNA1C,GNB2,GNAI1,GNAI3,GNG4,GNB0	KEGG
Network 5	hsa04935	Growth hormone synthesis, secretion and action	3	118	1.66	0.00056	CACNA1C,GNAI1,GNAI2	KEGG
Network 5	hsa05163	Human cytomegalovirus infection	9	218	1.87E+00	4.18E-15	GNB3,GNB4,GNG11,GNB5,GNB2,GNAI1,GNAI3,GNG4,GNB0	KEGG
Network 5	hsa05170	Human immunodeficiency virus 1 infection	9	204	1.89E+00	2.52E-15	GNB3,GNB4,GNG11,GNB5,GNB2,GNAI1,GNAI3,GNG4,GNB0	KEGG
Network 5	hsa05410	Hypertrophic cardiomyopathy	2	89	1.60E+00	0.0111	CACNA1C,CACNA2D1	KEGG
Network 5	HSA-2514859	Inactivation, recovery and regulation of the phototransduction cascade	2	33	2.03E+00	0.0053	GNB5,GNB0	REACTOME
Network 5	HSA-997272	Inhibition of voltage gated Ca2+ channels via Gbeta/gamma subunits	7	29	2.63E+00	1.05E-15	GNB3,GNB4,GNG11,GNB5,GNB2,GNG4,GNB0	REACTOME
Network 5	hsa05167	Kaposi sarcoma-associated herpesvirus infection	7	187	1.82E+00	5.64E-11	GNB3,GNB4,GNG11,GNB5,GNB2,GNG4,GNB0	KEGG
Network 5	hsa04670	Leukocyte transendothelial migration	2	109	1.51E+00	0.0136	GNAI1,GNAI2	KEGG
Network 5	hsa04730	Long-term depression	2	59	1.78E+00	0.0062	GNAI1,GNAI2	KEGG
Network 5	hsa04916	Melanogenesis	2	95	1.57E+00	0.0119	GNAI1,GNAI2	KEGG
Network 5	hsa05032	Morphine addiction	9	89	2.25E+00	3.10E-18	GNB3,GNB4,GNG11,GNB5,GNB2,GNAI1,GNAI3,GNG4,GNB0	KEGG
Network 5	WP289	Myometrial relaxation and contraction pathways	7	153	1.91E+00	1.16E-10	GNB3,GNB4,GNG11,GNB5,GNB2,GNG4,GNB0	wikipathways
Network 5	WP1602	Nicotine effect on dopaminergic neurons	2	21	2.23E+00	0.0049	GNAI1,GNB0	wikipathways
Network 5	hsa04921	Oxytocin signaling pathway	4	149	1.68E+00	1.91E-05	CACNA1C,GNAI1,GNAI3,CACNA2D1	KEGG
Network 5	hsa04928	Parathyroid hormone synthesis, secretion and action	2	103	1.54E+00	0.0128	GNAI1,GNAI2	KEGG
Network 5	hsa05200	Pathways in cancer	9	517	1.49E+00	8.01E-12	GNB3,GNB4,GNG11,GNB5,GNB2,GNAI1,GNAI3,GNG4,GNB0	KEGG
Network 5	hsa05133	Pertussis	2	74	1.68E+00	0.009	GNAI1,GNAI2	KEGG
Network 5	HSA-5576892	Phase 0 - rapid depolarisation	2	32	2.05E+00	0.0051	CACNA1C,CACNA2D1	REACTOME
Network 5	HSA-5576893	Phase 2 - plateau phase	2	15	2.38E+00	0.0013	CACNA1C,CACNA2D1	REACTOME
Network 5	hsa04151	PI3K-Akt signaling pathway	7	350	1.55E+00	3.66E-09	GNB3,GNB4,GNG11,GNB5,GNB2,GNG4,GNB0	KEGG
Network 5	WP4172	PI3K-Akt signaling pathway	7	336	1.57E+00	1.67E-08	GNB3,GNB4,GNG11,GNB5,GNB2,GNG4,GNB0	wikipathways
Network 5	hsa04611	Platelet activation	2	122	1.46E+00	0.0161	GNAI1,GNAI2	KEGG
Network 5	HSA-112043	PLC beta mediated events	2	53	1.83E+00	0.0128	GNAI1,GNAI2	REACTOME
Network 5	HSA-500657	Presynaptic function of Kainate receptors	7	21	2.77E+00	1.94E-16	GNB3,GNB4,GNG11,GNB5,GNB2,GNG4,GNB0	REACTOME
Network 5	hsa04914	Progesterone-mediated oocyte maturation	2	94	1.58	0.0119	GNAI1,GNAI2	KEGG
Network 5	HSA-392851	Prostacyclin signalling through prostacyclin receptor	7	19	2.82	1.27E-16	GNB3,GNB4,GNG11,GNB5,GNB2,GNG4,GNB0	REACTOME
Network 5	WP4900	Purinergic signaling	2	32	2.05	0.009	GNAI1,GNAI2	wikipathways
Network 5	hsa04015	Rap1 signaling pathway	2	202	1.25	0.0399	GNAI1,GNAI2	KEGG
Network 5	WP4223	Ras signaling	7	179	1.84	2.70E-10	GNB3,GNB4,GNG11,GNB5,GNB2,GNG4,GNB0	wikipathways
Network 5	hsa04014	Ras signaling pathway	7	226	1.74	1.94E-10	GNB3,GNB4,GNG11,GNB5,GNB2,GNG4,GNB0	KEGG
Network 5	hsa04923	Regulation of lipolysis in adipocytes	2	54	1.82	0.0054	GNAI1,GNAI2	KEGG
Network 5	hsa04926	Relaxin signaling pathway	9	128	2.1	6.26E-17	GNB3,GNB4,GNG11,GNB5,GNB2,GNAI1,GNAI3,GNG4,GNB0	KEGG
Network 5	hsa04924	Renin secretion	3	66	1.91	0.00011	CACNA1C,GNAI1,GNAI2	KEGG
Network 5	hsa04723	Retrograde endocannabinoid signaling	10	145	2.09	3.78E-19	GNB3,GNB4,GNG11,GNB5,CACNA1C,GNB2,GNAI1,GNAI3,GNG4,GNB0	KEGG
Network 5	WP26	S1P receptor signal transduction	2	25	2.15	0.0062	GNAI1,GNAI2	wikipathways
Network 5	hsa04726	Serotonergic synapse	10	108	2.22	5.25E-20	GNB3,GNB4,GNG11,GNB5,CACNA1C,GNB2,GNAI1,GNAI3,GNG4,GNB0	KEGG
Network 5	hsa04071	Sphingolipid signaling pathway	2	116	1.49	0.0149	GNAI1,GNAI2	KEGG
Network 5	hsa04742	Taste transduction	2	81	1.64	0.01	GNB3,CACNA1C	KEGG
Network 5	HSA-456926	Thrombin signalling through proteinase activated receptors (PARs)	7	32	2.59	1.57E-15	GNB3,GNB4,GNG11,GNB5,GNB2,GNG4,GNB0	REACTOME
Network 5	HSA-428930	Thromboxane signalling through TP receptor	7	24	2.71	3.74E-16	GNB3,GNB4,GNG11,GNB5,GNB2,GNG4,GNB0	REACTOME
Network 5	WP2032	Thyroid stimulating hormone (TSH) signaling pathway	3	66	1.91	0.00051	GNAI1,GNAI3,GNB0	wikipathways
Network 5	hsa05145	Toxoplasmosis	2	105	1.53	0.0129	GNAI1,GNAI2	KEGG
Network 5	HSA-112315	Transmission across Chemical Synapses	10	267	1.82	2.93E-16	GNB3,GNB4,GNG11,GNB5,GNB2,GNAI1,GNAI3,GNG4,CACNA2D2,GNB0	REACTOME
Network 5	HSA-432040	Vasopressin regulates renal water homeostasis via Aquaporins	7	43	2.46	8.34E-15	GNB3,GNB4,GNG11,GNB5,GNB2,GNG4,GNB0	REACTOME
Network 6	HSA-176187	Activation of ATR in response to replication stress	2	37	1.98E+00	0.031	RPA1,RAD0	REACTOME
Network 6	WP3875	ATR signaling	2	9	2.60E+00	0.0072	RPA1,RAD0	wikipathways
Network 6	HSA-1640170	Cell Cycle	4	647	1.04E+00	0.0424	RPA1,RAD51C,BRCA2,RAD0	REACTOME
Network 6	WP4016	DNA IR-damage and cellular response via ATR	3	77	1.84E+00	0.0072	RPA1,BRCA2,RAD0	wikipathways
Network 6	WP4946	DNA repair pathways, full network	3	118	1.66E+00	0.0072	RPA1,RAD51C,BRCA1	wikipathways
Network 6	HSA-6783310	Fanconi Anemia Pathway	2	37	1.98E+00	0.031	RPA1,MUS80	REACTOME
Network 6	hsa03460	Fanconi anemia pathway	4	51	2.14E+00	3.05E-06	RPA1,MUS81,RAD51C,BRCA1	KEGG
Network 6	HSA-5685942	HDR through Homologous Recombination (HRR)	10	65	2.44E+00	8.68E-21	RPA1,MUS81,GEN1,RAD51C,XRCC2,BRCA2,RAD1,RAD51B,XRCC3,RAD51D	REACTOME

Network #	term ID	term description	observed gene count	background gene count	strength	false discovery rate	Proteins	Lable
Network 6	HSA-5685938	HDR through Single Strand Annealing (SSA)	2	36	1.99E+00	0.031	RPA1,RAD0	REACTOME
Network 6	HSA-5693579	Homologous DNA Pairing and Strand Exchange	8	41	2.54E+00	4.30E-17	RPA1,RAD51C,XRCC2,BRCA2,RAD1,RAD51B,XRCC3,RAD51D	REACTOME
Network 6	hsa03440	Homologous recombination	8	39	2.56E+00	3.25E-17	RPA1,MUS81,RAD51C,XRCC2,BRCA2,RAD51B,XRCC3,ENSP00000466833	KEGG
Network 6	WP186	Homologous recombination	2	12	2.47E+00	0.0072	RPA1,BRCA1	wikipathways
Network 6	WP4673	Male infertility	3	141	1.58E+00	0.0084	XRCC2,BRCA2,XRCC2	wikipathways
Network 6	HSA-912446	Meiotic recombination	3	55	1.99E+00	0.00079	RPA1,RAD51C,BRCA1	REACTOME
Network 6	HSA-5693616	Presynaptic phase of homologous DNA pairing and strand exchange	7	38	2.52E+00	1.64E-14	RPA1,RAD51C,XRCC2,BRCA2,RAD1,RAD51B,RAD51D	REACTOME
Network 6	HSA-5693568	Resolution of D-loop Structures through Holliday Junction Intermediates	8	31	2.66	8.22E-18	MUS81,GEN1,RAD51C,XRCC2,BRCA2,RAD51B,XRCC3,RAD51D	REACTOME
Network 6	HSA-5693554	esolution of D-loop Structures through Synthesis-Dependent Strand Annealing (SDSA	6	25	2.63	9.40E-13	RAD51C,XRCC2,BRCA2,RAD51B,XRCC3,RAD51D	REACTOME
Network 7	HSA-1482788	Acyl chain remodelling of PC	2	27	2.12E+00	0.0292	LPCAT4,PLA2G15	REACTOME
Network 7	HSA-1482839	Acyl chain remodelling of PE	2	29	2.09E+00	0.0292	LPCAT4,PLA2G15	REACTOME
Network 7	HSA-1482801	Acyl chain remodelling of PS	2	22	2.21E+00	0.0244	LPCAT4,PLA2G15	REACTOME
Network 7	hsa00565	Ether lipid metabolism	6	47	2.36E+00	2.26E-11	CHPT1,EPT1,PLD2,LPCAT4,PLA2G16,PLD3	KEGG
Network 7	WP4722	Glycerolipids and glycerophospholipids	5	22	2.61	1.05E-09	CHPT1,PEMT,CDS1,PTDSS2,PISD	wikipathways
Network 7	HSA-1483206	Glycerophospholipid biosynthesis	10	128	2.14	5.16E-18	CHPT1,PEMT,EPT1,PLD2,CDS1,PTDSS2,LPCAT4,PLA2G16,PISD,PLD3	REACTOME
Network 7	WP2533	Glycerophospholipid biosynthetic pathway	2	30	2.07	0.0239	CHPT1,PEMT	wikipathways
Network 7	hsa00564	Glycerophospholipid metabolism	11	97	2.3	2.87E-23	CHPT1,PEMT,EPT1,PLD2,LCAT,CDS1,PTDSS2,LPCAT4,PLA2G16,PISD,PLD3	KEGG
Network 7	WP3933	Kennedy pathway from sphingolipids	4	14	2.71E+00	5.57E-08	CHPT1,PEMT,PTDSS2,PISD	wikipathways
Network 7	hsa01100	Metabolic pathways	10	1447	1.09E+00	5.82E-09	CHPT1,PEMT,EPT1,PLD2,CDS1,PTDSS2,LPCAT4,PLA2G16,PISD,PLD3	KEGG
Network 7	WP4288	MTHFR deficiency	2	25	2.15E+00	0.0226	CHPT1,PEMT	wikipathways
Network 7	hsa00440	Phosphonate and phosphinate metabolism	2	6	2.77E+00	0.00067	CHPT1,EPT0	KEGG
Network 7	HSA-2029485	Role of phospholipids in phagocytosis	2	26	2.14	0.0292	PLD2,PLD3	REACTOME
Network 7	HSA-1483166	Synthesis of PA	2	39	1.96	0.0421	PLD2,LPCAT3	REACTOME
Network 7	HSA-1483191	Synthesis of PC	2	28	2.1	0.0292	CHPT1,PEMT	REACTOME
Network 7	HSA-1483213	Synthesis of PE	2	13	2.44	0.0109	EPT1,PISD	REACTOME
Network 7	HSA-1483148	Synthesis of PG	2	8	2.65	0.0056	PLD2,PLD3	REACTOME
Network 8	HSA-6807878	COPI-mediated anterograde transport	9	100	2.20E+00	1.16E-16	COG3,STX5,COG5,COG1,COG7,COG8,COG4,COG2,COG5	REACTOME
Network 8	HSA-6811438	Intra-Golgi traffic	10	43	2.62E+00	2.05E-22	STX6,COG3,STX5,COG5,COG1,COG7,COG8,COG4,COG2,COG5	REACTOME
Network 8	HSA-6811440	Retrograde transport at the Trans-Golgi-Network	9	49	2.51	5.45E-19	STX6,COG3,COG5,COG1,COG7,COG8,COG4,COG2,COG5	REACTOME
Network 9	HSA-450385	Butyrate Response Factor 1 (BRF1) binds and destabilizes mRNA	2	17	2.36E+00	0.0217	DCP2,DCP1A	REACTOME
Network 9	HSA-429914	Deadenylation-dependent mRNA decay	7	55	2.40E+00	3.16E-13	PATL1,EDC3,CNOT1,EDC4,DCP2,DCP1A,DDX5	REACTOME
Network 9	HSA-430039	mRNA decay by 5 to 3 exoribonuclease	6	15	2.89E+00	3.16E-13	PATL1,EDC3,EDC4,DCP2,DCP1A,DDX5	REACTOME
Network 9	hsa03018	RNA degradation	6	75	2.19	2.89E-10	PATL1,EDC3,CNOT1,EDC4,DCP2,DDX5	KEGG
Network 9	HSA-450513	Tristetraprolin (TTP, ZFP36) binds and destabilizes mRNA	2	17	2.36	0.0217	DCP2,DCP1A	REACTOME
Network 10	HSA-5250924	B-WICH complex positively regulates rRNA expression	3	59	1.96E+00	0.00071	POLR1A,POLR2L,POLR1C	REACTOME
Network 10	hsa04623	Cytosolic DNA-sensing pathway	10	62	2.46E+00	4.35E-22	POLR3B,POLR3E,POLR1D,POLR2L,POLR3C,POLR3H,POLR3A,POLR1C,POLR3F,POLR3D	KEGG
Network 10	WP4655	Cytosolic DNA-sensing pathway	10	73	2.39E+00	3.98E-21	POLR3B,POLR3E,POLR1D,POLR2L,POLR3C,POLR3H,POLR3A,POLR1C,POLR3F,POLR3D	wikipathways
Network 10	HSA-1834949	Cytosolic sensors of pathogen-associated DNA	9	62	2.41E+00	8.46E-19	POLR3B,POLR3E,POLR2L,POLR3C,POLR3H,POLR3A,POLR1C,POLR3F,POLR3D	REACTOME
Network 10	WP405	Eukaryotic transcription initiation	6	42	2.41E+00	1.62E-11	POLR3B,POLR1A,POLR3E,POLR1D,POLR3H,POLR3D	wikipathways
Network 10	HSA-74160	Gene expression (Transcription)	10	1455	1.09	1.19E-08	POLR3B,POLR1A,POLR3E,POLR2L,POLR3C,POLR3H,POLR3A,POLR1C,POLR3F,POLR3D	REACTOME
Network 10	HSA-427413	NoRC negatively regulates rRNA expression	3	74	1.86E+00	0.0012	POLR1A,POLR2L,POLR1C	REACTOME
Network 10	WP4022	Pyrimidine metabolism	11	84	2.37	1.31E-23	POLR3B,POLR1A,POLR3E,POLR1D,POLR2L,POLR3C,POLR3H,POLR3A,POLR1C,POLR3F,POLR3D	wikipathways
Network 10	hsa03020	RNA polymerase	11	31	2.8	3.56E-28	POLR3B,POLR1A,POLR3E,POLR1D,POLR2L,POLR3C,POLR3H,POLR3A,POLR1C,POLR3F,POLR3D	KEGG
Network 10	HSA-73772	RNA Polymerase I Promoter Escape	3	59	1.96	0.00071	POLR1A,POLR2L,POLR1C	REACTOME
Network 10	HSA-73762	RNA Polymerase I Transcription Initiation	3	46	2.06	0.00037	POLR1A,POLR2L,POLR1C	REACTOME
Network 10	HSA-73863	RNA Polymerase I Transcription Termination	3	30	2.25	0.00012	POLR1A,POLR2L,POLR1C	REACTOME
Network 10	HSA-749476	RNA Polymerase III Abortive And Retractive Initiation	9	40	2.6	3.01E-20	POLR3B,POLR3E,POLR2L,POLR3C,POLR3H,POLR3A,POLR1C,POLR3F,POLR3D	REACTOME
Network 10	HSA-73780	RNA Polymerase III Chain Elongation	9	17	2.97	3.21E-22	POLR3B,POLR3E,POLR2L,POLR3C,POLR3H,POLR3A,POLR1C,POLR3F,POLR3D	REACTOME
Network 10	HSA-76061	RNA Polymerase III Transcription Initiation From Type 1 Promoter	9	27	2.77	2.41E-21	POLR3B,POLR3E,POLR2L,POLR3C,POLR3H,POLR3A,POLR1C,POLR3F,POLR3D	REACTOME
Network 10	HSA-76066	RNA Polymerase III Transcription Initiation From Type 2 Promoter	9	26	2.79	2.41E-21	POLR3B,POLR3E,POLR2L,POLR3C,POLR3H,POLR3A,POLR1C,POLR3F,POLR3D	REACTOME
Network 10	HSA-76071	RNA Polymerase III Transcription Initiation From Type 3 Promoter	9	27	2.77	2.41E-21	POLR3B,POLR3E,POLR2L,POLR3C,POLR3H,POLR3A,POLR1C,POLR3F,POLR3D	REACTOME
Network 10	HSA-73980	RNA Polymerase III Transcription Termination	9	22	2.86	1.03E-21	POLR3B,POLR3E,POLR2L,POLR3C,POLR3H,POLR3A,POLR1C,POLR3F,POLR3D	REACTOME
Network 11	HSA-5617472	ctivation of anterior HOX genes in hindbrain development during early embryogenesis	5	91	1.99E+00	3.13E-07	EED,SUZ12,EZH2,RBBP4,RBBP6	REACTOME
Network 11	HSA-606279	Deposition of new CENPA-containing nucleosomes at the centromere	2	52	1.84E+00	0.0338	RBBP4,RBBP6	REACTOME
Network 11	WP4320	Effect of progerin on genes involved in Hutchinson-Gilford progeria syndrome	2	25	2.15E+00	0.0085	RBBP4,RBBP6	wikipathways
Network 11	WP2853	Endoderm differentiation	4	140	1.71E+00	0.00012	EZH2,JARID2,MTF2,AEBP1	wikipathways
Network 11	WP4239	Epithelial to mesenchymal transition in colorectal cancer	4	160	1.65E+00	0.00017	EED,SUZ12,EZH2,RBBP3	wikipathways
Network 11	HSA-427389	ERCC6 (CSB) and EHMT2 (G9a) positively regulate rRNA expression	2	45	1.90E+00	0.0277	RBBP4,RBBP6	REACTOME
Network 11	WP4553	FBXL10 enhancement of MAP/ERK signaling in diffuse large B-cell lymphoma	3	22	2.38	5.11E-05	EED,SUZ12,EZH1	wikipathways
Network 11	HSA-9609690	HCMV Early Events	5	102	1.94E+00	3.94E-07	EED,SUZ12,EZH2,RBBP4,RBBP6	REACTOME
Network 11	HSA-3214815	HDACs deacetylate histones	2	60	1.77E+00	0.0399	RBBP4,RBBP6	REACTOME
Network 11	WP2369	Histone modifications	5	44	2.31E+00	1.23E-08	EED,SETDB1,EZH2,AEBP2,EZH0	wikipathways
Network 11	WP2916	Interactome of polycomb repressive complex 2 (PRC2)	9	16	3.00E+00	6.55E-23	EED,SUZ12,EZH2,JARID2,MTF2,RBBP4,RBBP7,AEBP2,EZH0	wikipathways
Network 11	hsa00310	Lysine degradation	3	60	1.95E+00	0.0017	SETDB1,EZH2,EZH0	KEGG
Network 11	WP2857	Mesodermal commitment pathway	3	145	1.57E+00	0.0065	JARID2,MTF2,AEBP1	wikipathways
Network 11	HSA-2559580	Oxidative Stress Induced Senescence	5	93	1.98E+00	3.13E-07	EED,SUZ12,EZH2,RBBP4,RBBP6	REACTOME
Network 11	HSA-3214841	PKMTs methylate histone lysines	7	47	2.42E+00	1.92E-13	EED,SETDB1,SUZ12,EZH2,RBBP4,RBBP7,AEBP1	REACTOME
Network 11	HSA-212300	PRC2 methylates histones and DNA	9	42	2.58E+00	3.12E-19	EED,SUZ12,EZH2,JARID2,MTF2,RBBP4,RBBP7,AEBP2,PHF18	REACTOME
Network 11	HSA-8943724	Regulation of PTEN gene transcription	5	60	2.17	4.87E-08	EED,SUZ12,EZH2,RBBP4,RBBP6	REACTOME
Network 11	HSA-6804758	Regulation of TP53 Activity through Acetylation	2	30	2.07	0.0146	RBBP4,RBBP6	REACTOME
Network 11	HSA-73762	RNA Polymerase I Transcription Initiation	2	46	1.89	0.0277	RBBP4,RBBP6	REACTOME
Network 11	HSA-8953750	Transcriptional Regulation by E2F6	5	32	2.44	4.53E-09	EED,SUZ12,EZH2,RBBP4,RBBP6	REACTOME
Network 11	WP4204	Tumor suppressor activity of SMARCB1	4	30	2.38	5.60E-07	EED,SUZ12,EZH2,RBBP3	wikipathways
Network 12	HSA-446203	Asparagine N-linked glycosylation	6	300	1.55E+00	1.30E-06	FUT3,B4GALT2,B4GALT1,B4GALT4,B4GALT3,ST3GAL5	REACTOME
Network 12	HSA-9033658	Blood group systems biosynthesis	5	20	2.65E+00	5.54E-10	FUT9,FUT3,FUT1,FUT5,ST3GAL5	REACTOME
Network 12	hsa00052	Galactose metabolism	2	30	2.07	0.0053	B4GALT2,B4GALT0	KEGG
Network 12	WP1424	Globo sphingolipid metabolism	2	21	2.23	0.0447	FUT9,FUT0	wikipathways
Network 12	hsa00533	Glycosaminoglycan biosynthesis - keratan sulfate	5	13	2.84	2.77E-11	B3GNT2,B4GALT2,B4GALT1,B4GALT4,B4GALT2	KEGG
Network 12	hsa00603	Glycosphingolipid biosynthesis - globo and isoglobo series	2	15	2.38	0.0016	FUT9,FUT0	KEGG
Network 12	hsa00601	Glycosphingolipid biosynthesis - lacto and neolacto series	11	26	2.88	7.10E-29	FUT9,B3GNT2,FUT3,B4GALT2,FUT1,GCNT2,B4GALT1,B4GALT4,FUT5,B4GALT3,ST3GAL5	KEGG

Network #	term ID	term description	observed gene count	background gene count	strength	false discovery rate	Proteins	Lable
Network 12	HSA-2022854	Keratan sulfate biosynthesis	6	27	2.60E+00	7.07E-12	B3GNT2,B4GALT2,B4GALT1,B4GALT4,B4GALT3,ST3GAL5	REACTOME
Network 12	HSA-9037629	Lewis blood group biosynthesis	4	17	2.62E+00	8.72E-08	FUT9,FUT3,FUT5,ST3GAL5	REACTOME
Network 12	hsa00515	Mannose type O-glycan biosynthesis	4	22	2.51E+00	6.73E-08	FUT9,B4GALT2,B4GALT1,B4GALT2	KEGG
Network 12	hsa01100	Metabolic pathways	11	1447	1.13E+00	4.23E-11	FUT9,B3GNT2,FUT3,B4GALT2,FUT1,GCNT2,B4GALT1,B4GALT4,FUT5,B4GALT3,ST3GAL5	KEGG
Network 12	HSA-71387	Metabolism of carbohydrates	10	292	1.78E+00	1.55E-14	FUT9,B3GNT2,FUT3,B4GALT2,FUT1,B4GALT1,B4GALT4,FUT5,B4GALT3,ST3GAL5	REACTOME
Network 12	WP4142	Metabolism of spingolipids in ER and Golgi apparatus	2	20	2.25E+00	0.0447	B4GALT2,B4GALT0	wikipathways
Network 12	HSA-975577	N-Glycan antennae elongation	4	15	2.68E+00	6.46E-08	B4GALT2,B4GALT1,B4GALT4,B4GALT2	REACTOME
Network 12	HSA-975576	N-glycan antennae elongation in the medial/trans-Golgi	5	25	2.55E+00	1.19E-09	FUT3,B4GALT2,B4GALT1,B4GALT4,B4GALT2	REACTOME
Network 12	hsa00510	N-Glycan biosynthesis	3	50	2.03E+00	0.00015	B4GALT2,B4GALT1,B4GALT2	KEGG
Network 12	hsa00514	Other types of O-glycan biosynthesis	3	43	2.09E+00	0.00011	B4GALT2,B4GALT1,B4GALT2	KEGG
Network 12	HSA-597592	Post-translational protein modification	7	1390	9.50E-01	0.00043	B3GNT2,FUT3,B4GALT2,B4GALT1,B4GALT4,B4GALT3,ST3GAL5	REACTOME
Network 12	HSA-1912420	Pre-NOTCH Processing in Golgi	2	17	2.32E+00	0.0076	B4GALT1,ST3GAL5	REACTOME
Network 12	hsa00513	Various types of N-glycan biosynthesis	3	38	2.15	9.34E-05	B4GALT2,B4GALT1,B4GALT2	KEGG
Network 13	HSA-264642	Acetylcholine Neurotransmitter Release Cycle	2	17	2.36E+00	0.0062	SYT1,VAMP1	REACTOME
Network 13	HSA-9609736	Assembly and cell surface presentation of NMDA receptors	5	42	2.37E+00	4.34E-09	LIN7B,APBA1,LIN7C,CASK,LIN7A	REACTOME
Network 13	HSA-212676	Dopamine Neurotransmitter Release Cycle	10	23	2.93E+00	8.85E-26	LIN7B,SYT1,APBA1,LIN7C,SYN1,VAMP2,SYN3,CASK,LIN7A,SYN1	REACTOME
Network 13	HSA-888590	GABA synthesis, release, reuptake and degradation	2	19	2.31	0.0067	SYT1,VAMP1	REACTOME
Network 13	HSA-210500	Glutamate Neurotransmitter Release Cycle	2	24	2.21	9.70E-03	SYT1,VAMP1	REACTOME
Network 13	HSA-6794361	Neurexins and neuroligins	6	55	2.33E+00	6.42E-11	LIN7B,SYT1,APBA1,LIN7C,CASK,LIN7A	REACTOME
Network 13	HSA-181430	Norepinephrine Neurotransmitter Release Cycle	2	18	2.34E+00	0.0064	SYT1,VAMP1	REACTOME
Network 13	HSA-181429	Serotonin Neurotransmitter Release Cycle	5	18	2.74	1.28E-10	SYT1,SYN1,VAMP2,SYN3,SYN1	REACTOME
Network 13	WP2267	Synaptic vesicle pathway	5	51	2.28	2.70E-08	SYT1,SYN1,VAMP2,SYN3,SYN1	wikipathways
Network 13	HSA-5250958	Toxicity of botulinum toxin type B (botB)	2	3	3.12	4.60E-04	SYT1,VAMP1	REACTOME
Network 13	HSA-5250989	Toxicity of botulinum toxin type G (botG)	2	3	3.12	0.00046	SYT1,VAMP1	REACTOME
Network 14	HSA-140875	Common Pathway of Fibrin Clot Formation	3	22	2.58E+00	1.40E-04	PROCR,PRTN3,CD176	REACTOME
Network 14	HSA-109582	Hemostasis	4	605	1.27E+00	1.31E-02	PROCR,PRTN3,SERPINA1,CD176	REACTOME
Network 14	HSA-6798695	Neutrophil degranulation	5	473	1.47E+00	1.90E-04	MPO,PRTN3,CAMP,SERPINA1,CD176	REACTOME
Network 15	hsa04514	Cell adhesion molecules	5	137	2.01E+00	1.31E-07	PVRL2,CD226,ITGAL,PVR,TIGIT	KEGG
Network 15	HSA-198933	Immunoregulatory interactions between a Lymphoid and a non-Lymphoid cell	5	129	2.03E+00	6.29E-07	KLRK1,PVRL2,CD226,ITGAL,PVR	REACTOME
Network 15	WP5092	Interactions of natural killer cells in pancreatic cancer	4	27	2.62E+00	1.22E-07	KLRK1,CD226,PVR,TIGIT	wikipathways
Network 15	hsa05144	Malaria	2	46	2.08E+00	2.06E-02	KLRK1,ITGAL	KEGG
Network 15	HSA-420597	Nectin/Necl trans heterodimerization	2	7	2.90E+00	0.0029	PVRL2,PVR	REACTOME
Network 16	HSA-499943	Interconversion of nucleotide di- and triphosphates	2	29	2.29E+00	0.0157	AK5,GUK0	REACTOME
Network 16	hsa01100	Metabolic pathways	7	1447	1.13E+00	2.07E-06	DGUOK,AK5,PNP,GUK1,CTH,ITPA,NT5M	KEGG
Network 16	HSA-1430728	Metabolism	7	2089	9.70E-01	0.00017	DGUOK,AK5,PNP,GUK1,CTH,ITPA,NT5M	REACTOME
Network 16	HSA-15869	Metabolism of nucleotides	6	99	2.23E+00	3.12E-10	DGUOK,AK5,PNP,GUK1,ITPA,NT5M	REACTOME
Network 16	hsa00760	Nicotinate and nicotinamide metabolism	2	34	2.22E+00	0.0077	PNP,NT5M	KEGG
Network 16	HSA-8956319	Nucleobase catabolism	3	36	2.37E+00	0.00018	PNP,ITPA,NT5M	REACTOME
Network 16	HSA-74259	Purine catabolism	2	18	2.49	0.009	PNP,ITPA	REACTOME
Network 16	hsa00230	Purine metabolism	6	127	2.12	2.06E-10	DGUOK,AK5,PNP,GUK1,ITPA,NT5M	KEGG
Network 16	WP4792	Purine metabolism	3	13	2.81	1.06E-05	DGUOK,PNP,ITPA	wikipathways
Network 16	WP4224	Purine metabolism and related disorders	3	22	2.58	2.18E-05	DGUOK,PNP,ITPA	wikipathways
Network 16	HSA-74217	Purine salvage	2	13	2.63	0.0062	DGUOK,PNP	REACTOME
Network 16	hsa00240	Pyrimidine metabolism	2	56	2	0.0151	PNP,NT5M	KEGG
Network 17	HSA-432722	Golgi Associated Vesicle Biogenesis	5	56	2.46	3.23E-09	TBC1D8B,DNAJC6,TPD52,HSPA8,TPD52L0	REACTOME
Network 17	HSA-432720	Lysosome Vesicle Biogenesis	2	35	2.27E+00	0.0226	DNAJC6,HSPA7	REACTOME
Network 18	hsa04060	Cytokine-cytokine receptor interaction	6	282	1.84E+00	1.08E-09	IL22RA1,IL20RA,IL20RB,IL19,IL20,IL23	KEGG
Network 18	HSA-8854691	Interleukin-20 family signaling	6	25	2.89E+00	2.05E-14	IL22RA1,IL20RA,IL20RB,IL19,IL20,IL23	REACTOME
Network 18	hsa04630	JAK-STAT signaling pathway	6	160	2.09E+00	5.71E-11	IL22RA1,IL20RA,IL20RB,IL19,IL20,IL23	KEGG
Network 18	WP4538	Regulatory circuits of the STAT3 signaling pathway	3	78	2.1	0.00092	IL22RA1,IL20RA,IL20RB	wikipathways
Network 18	hsa04061	Viral protein interaction with cytokine and cytokine receptor	6	96	2.31	5.80E-12	IL22RA1,IL20RA,IL20RB,IL19,IL20,IL23	KEGG
Network 19	HSA-112126	ALKBH3 mediated reversal of alkylation damage	4	4	3.59E+00	1.24E-10	ALKBH3,ASCC1,ASCC3,ASCC1	REACTOME
Network 21	HSA-5669034	TNFs bind their physiological receptors	3	28	2.72	3.12E-05	TNFRSF6B,TNFSF15,TNFRSF24	REACTOME
Network 22	hsa04728	Dopaminergic synapse	2	128	1.88E+00	0.0239	GNAS,ARRB0	KEGG
Network 22	hsa05163	Human cytomegalovirus infection	2	218	1.65E+00	0.0357	PTGER4,GNAS	KEGG
Network 22	hsa04750	Inflammatory mediator regulation of TRP channels	2	94	2.02E+00	0.0239	PTGER4,GNAS	KEGG
Network 22	hsa05032	Morphine addiction	2	89	2.04E+00	0.0239	GNAS,ARRB0	KEGG
Network 22	hsa04928	Parathyroid hormone synthesis, secretion and action	2	103	1.98E+00	0.0239	GNAS,ARRB0	KEGG
Network 22	hsa04926	Relaxin signaling pathway	2	128	1.88	0.0239	GNAS,ARRB0	KEGG
Network 22	hsa04924	Renin secretion	2	66	2.17	0.0239	PTGER4,GNAS	KEGG
Network 24	WP2328	Allograft Rejection	2	89	2.17E+00	0.0217	CXCL13,CXCR4	wikipathways
Network 25	HSA-380108	Chemokine receptors bind chemokines	2	57	2.36E+00	0.0291	CXCL13,CXCR4	REACTOME
Network 26	hsa04062	Chemokine signaling pathway	3	186	2.02E+00	0.0003	CXCL13,CXCR5,GNAI1	KEGG
Network 27	WP3929	Chemokine signaling pathway	3	164	2.08E+00	0.00041	CXCL13,CXCR5,GNAI1	wikipathways
Network 28	HSA-418594	G alpha (i) signalling events	3	396	1.69	0.0183	CXCL13,CXCR5,GNAI1	REACTOME
Network 29	hsa04061	Viral protein interaction with cytokine and cytokine receptor	2	96	2.13	0.0125	CXCL13,CXCR4	KEGG
Network 30	HSA-5210891	Uptake and function of anthrax toxins	3	11	3.25	6.33E-07	ANTXR1,ANTXR2,FURIN	REACTOME
Network 31	HSA-8949215	Mitochondrial calcium ion transport	2	23	2.93E+00	0.0034	SLC8A3,AKAP0	REACTOME
Network 32	hsa04020	Calcium signaling pathway	2	193	2.01E+00	0.0332	TPCN2,TPCN0	KEGG
Network 33	HSA-5620912	Anchoring of the basal body to the plasma membrane	2	97	2.30E+00	0.0282	CEP57,CEP62	REACTOME
Network 34	HSA-8854518	AURKA Activation by TPX2	2	72	2.43E+00	0.0282	CEP57,CEP62	REACTOME
Network 35	HSA-380259	Loss of Nlp from mitotic centrosomes	2	69	2.45E+00	0.0282	CEP57,CEP62	REACTOME
Network 36	HSA-380270	Recruitment of mitotic centrosome proteins and complexes	2	80	2.39	0.0282	CEP57,CEP62	REACTOME
Network 37	HSA-380320	Recruitment of NuMA to mitotic centrosomes	2	93	2.32	0.0282	CEP57,CEP62	REACTOME
Network 38	HSA-2565942	Regulation of PLK1 Activity at G2/M Transition	2	87	2.35	0.0282	CEP57,CEP62	REACTOME
Network 39	HSA-3214842	HDMs demethylate histones	2	24	2.91E+00	0.0037	ARID5B,PHF1	REACTOME
Network 40	HSA-5669034	TNFs bind their physiological receptors	2	28	2.84	0.0049	TNFSF4,TNFRSF3	REACTOME
