# Supplementary material for: Integrative molecular network analysis of genetic risk factors to infer biomarkers and therapeutic targets for rheumatoid arthritis
Source: PLoS One. 2025 Aug 21;20(8):e0329101. doi: 10.1371/journal.pone.0329101 (PMC12370121; doi:10.1371/journal.pone.0329101)
Supplement: S5 Table — (PDF) [file pone.0329101.s005.pdf]

Gene	Type	Note
AAT	Target	DrugBank
ACD	Target	DrugBank
ACE	Target	DrugBank
ACF	Target	DrugBank
ACR	Target	DrugBank
ACTB	Target	DrugBank
ADA	Target	DrugBank
ADA1	Target	DrugBank
ADA2	Target	DrugBank
ADAM17	Target	DrugBank
ADAM8	Target	DrugBank
ADAMTS12	Target	DrugBank
ADAMTS5	Target	DrugBank
CAMLG	Target	DrugBank
CASP1	Target	DrugBank
CASP3	Target	DrugBank
CCND1	Target	DrugBank
CFTR	Target	DrugBank
CHUK	Target	DrugBank
CLCNKA	Target	DrugBank
CRH	Target	DrugBank
CXCR1	Target	DrugBank
EDNRA	Target	DrugBank
FABP2	Target	DrugBank
FCGR1A	Target	DrugBank
FCGR2B	Target	DrugBank
FCGR2C	Target	DrugBank
FCGR3A	Target	DrugBank
FCGR3B	Target	DrugBank
GABRA1	Target	DrugBank
GLRA1	Target	DrugBank

Gene	Type	Note
GP1BA	Target	DrugBank
GST	Target	DrugBank
GSTA2	Target	DrugBank
GSTM1	Target	DrugBank
IL1R1	Target	DrugBank
HRH2	Target	DrugBank
HSD11B2	Target	DrugBank
HSD3B1	Target	DrugBank
HSPA5	Target	DrugBank
IKBKB	Target	DrugBank
KCNQ2	Target	DrugBank
KCNQ3	Target	DrugBank
LTA	Target	DrugBank
MAPK2	Target	DrugBank
MAPK3	Target	DrugBank
MC2R	Target	DrugBank
MYC	Target	DrugBank
NEU1	Target	DrugBank
NFKB1	Target	DrugBank
NFKB2	Target	DrugBank
NFKBIA	Target	DrugBank
NOS2	Target	DrugBank
NR0B1	Target	DrugBank
NR1I2	Target	DrugBank
NR3C1	Target	DrugBank
pab	Target	DrugBank
PCNA	Target	DrugBank
PDPK1	Target	DrugBank
PLA2G1B	Target	DrugBank
PLA2G4A	Target	DrugBank

Gene	Type	Note
PPARA	Target	DrugBank
PPARD	Target	DrugBank
PPIA	Target	DrugBank
PPIF	Target	DrugBank
PPP3R2	Target	DrugBank
PRDX6	Target	DrugBank
PRKA	Target	DrugBank
PTGDR2	Target	DrugBank
PTGER1	Target	DrugBank
PTGES3	Target	DrugBank
PTGS1	Target	DrugBank
PTGS2	Target	DrugBank
PTK2B	Target	DrugBank
RAC1	Target	DrugBank
RPS6KA3	Target	DrugBank
RXRA	Target	DrugBank
S100A7	Target	DrugBank
SCN10A	Target	DrugBank
SLC7A11	Target	DrugBank
THBD	Target	DrugBank
TLR9	Target	DrugBank
TNFAIP6	Target	DrugBank
TRPV1	Target	DrugBank
UGT1A9	Target	DrugBank
A20	Risk gene	GWAS
ACOXL	Risk gene	GWAS
ACPA	Risk gene	GWAS
AFF3	Risk gene	GWAS
AGR3-AHR	Risk gene	GWAS
AIM2	Risk gene	GWAS

Gene	Type	Note
AIRE	Risk gene	GWAS
AP11- LINC023	Risk gene	GWAS
ANKRD55	Risk gene	GWAS
ANTXR2	Risk gene	GWAS
ANXA3	Risk gene	GWAS
ARHGAP26	Risk gene	GWAS
ARID3A	Risk gene	GWAS
ARID5B	Risk gene	GWAS
B3GNT2	Risk gene	GWAS
BACH2	Risk gene	GWAS
BAD	Risk gene	GWAS
BATF	Risk gene	GWAS
BLK	Risk gene	GWAS
C1QBP	Risk gene	GWAS
C20orf181	Risk gene	GWAS
CASP8	Risk gene	GWAS
ASTOR3-SPDYE	Risk gene	GWAS
CCL21	Risk gene	GWAS
CCN4	Risk gene	GWAS
CCR6	Risk gene	GWAS
CD2	Risk gene	GWAS
CD226	Risk gene	GWAS
CD244	Risk gene	GWAS
CD28	Risk gene	GWAS
CD40	Risk gene	GWAS
CD83	Risk gene	GWAS
CDK2	Risk gene	GWAS
CDK5RAP2	Risk gene	GWAS
CDK6	Risk gene	GWAS
CEP57	Risk gene	GWAS

Gene	Type	Note
CLNK	Risk gene	GWAS
CLYBL	Risk gene	GWAS
COG6	Risk gene	GWAS
CRP	Risk gene	GWAS
CSE	Risk gene	GWAS
CSF2	Risk gene	GWAS
2RB-LOC105373	Risk gene	GWAS
CTLA4	Risk gene	GWAS
CUL5	Risk gene	GWAS
CXCR5	Risk gene	GWAS
DAP	Risk gene	GWAS
DDX6	Risk gene	GWAS
DGUOK-AS1	Risk gene	GWAS
DLGAP1	Risk gene	GWAS
DNASE1L3	Risk gene	GWAS
DPP4	Risk gene	GWAS
DR4	Risk gene	GWAS
EOMES	Risk gene	GWAS
ETV7	Risk gene	GWAS
FADS1	Risk gene	GWAS
FADS2	Risk gene	GWAS
FADS3	Risk gene	GWAS
FAM193A	Risk gene	GWAS
FcgR	Risk gene	GWAS
FCGR3A	Risk gene	GWAS
FCRL3	Risk gene	GWAS
FLI1-ETS1	Risk gene	GWAS
FLT-1	Risk gene	GWAS
FTL3	Risk gene	GWAS
FOXO3	Risk gene	GWAS

Gene	Type	Note
GATA3	Risk gene	GWAS
GFI1	Risk gene	GWAS
GNG4	Risk gene	GWAS
GPR174-KIF4CP	Risk gene	GWAS
GRHL2	Risk gene	GWAS
GSDMB	Risk gene	GWAS
GTF2IRD1-NCF1	Risk gene	GWAS
HAPLN4	Risk gene	GWAS
HIVEP3	Risk gene	GWAS
HLA-DRB1	Risk gene	GWAS
IFI144	Risk gene	GWAS
IFI16	Risk gene	GWAS
IFNGR2	Risk gene	GWAS
IKZF1	Risk gene	GWAS
IL10	Risk gene	GWAS
IL12RB2	Risk gene	GWAS
IL17A	Risk gene	GWAS
IL1beta	Risk gene	GWAS
IL1RN	Risk gene	GWAS
IL20RB	Risk gene	GWAS
IL2RA	Risk gene	GWAS
IL6	Risk gene	GWAS
IL6R	Risk gene	GWAS
ILF3	Risk gene	GWAS
IQGAP1	Risk gene	GWAS
IRAK1	Risk gene	GWAS
IRF4	Risk gene	GWAS
IRF5	Risk gene	GWAS
IRF8	Risk gene	GWAS
JARID2	Risk gene	GWAS

Gene	Type	Note
JAZF1	Risk gene	GWAS
KIAA1109	Risk gene	GWAS
KMT2B	Risk gene	GWAS
KSR1	Risk gene	GWAS
LBH	Risk gene	GWAS
LEF1	Risk gene	GWAS
LINC01898	Risk gene	GWAS
LMP2	Risk gene	GWAS
LOC105369698	Risk gene	GWAS
LOC107984408	Risk gene	GWAS
MACIR	Risk gene	GWAS
MED1	Risk gene	GWAS
MIC-1	Risk gene	GWAS
MIR146A	Risk gene	GWAS
MMEL1	Risk gene	GWAS
MTF1	Risk gene	GWAS
NFKBE	Risk gene	GWAS
NRAMP1	Risk gene	GWAS
NRSN1	Risk gene	GWAS
OS9-AGAP2	Risk gene	GWAS
PADI4	Risk gene	GWAS
PCBP1-AS1	Risk gene	GWAS
PDE2A-ARAP1	Risk gene	GWAS
LCL1-LINC0192	Risk gene	GWAS
PLCL2	Risk gene	GWAS
PLD4	Risk gene	GWAS
PLGRKT	Risk gene	GWAS
POLR1C	Risk gene	GWAS
POU3F1	Risk gene	GWAS
PPIL4	Risk gene	GWAS

Gene	Type	Note
PRDM1-ATG5	Risk gene	GWAS
PRKCB	Risk gene	GWAS
PRKCH	Risk gene	GWAS
PRKCQ	Risk gene	GWAS
PSORS1C1	Risk gene	GWAS
PSTPIP1	Risk gene	GWAS
PTGER4	Risk gene	GWAS
PTGS2	Risk gene	GWAS
PTPN2	Risk gene	GWAS
PTPN22	Risk gene	GWAS
PTPRC	Risk gene	GWAS
PVT1	Risk gene	GWAS
RAD51B	Risk gene	GWAS
RASGRP1	Risk gene	GWAS
RBPJ	Risk gene	GWAS
REL	Risk gene	GWAS
RTKN2	Risk gene	GWAS
RUNX1	Risk gene	GWAS
SCAF11	Risk gene	GWAS
SFTPD	Risk gene	GWAS
SH2B3-PTPN11	Risk gene	GWAS
SIGLEC6	Risk gene	GWAS
SKAP2-HOXA1	Risk gene	GWAS
SLAMF6	Risk gene	GWAS
SLC8A3	Risk gene	GWAS
SMC1B	Risk gene	GWAS
SPRED2	Risk gene	GWAS
STAT4	Risk gene	GWAS
SWAP70	Risk gene	GWAS
SYNGR1	Risk gene	GWAS

Gene	Type	Note
TAGAP	Risk gene	GWAS
TAMM41-SYN2	Risk gene	GWAS
TBX3	Risk gene	GWAS
TEC	Risk gene	GWAS
TGFB1	Risk gene	GWAS
TL1A	Risk gene	GWAS
TLE3	Risk gene	GWAS
TLR8	Risk gene	GWAS
TNFAIP3	Risk gene	GWAS
TNFalpha	Risk gene	GWAS
TNFRSF1A	Risk gene	GWAS
TNFRSF9	Risk gene	GWAS
TNFSF4	Risk gene	GWAS
TNIP1	Risk gene	GWAS
TPCN2	Risk gene	GWAS
TPD52	Risk gene	GWAS
TPRA1	Risk gene	GWAS
TPRG1-TP63	Risk gene	GWAS
TRAF1-C5	Risk gene	GWAS
TSPAN32	Risk gene	GWAS
TXNDC11	Risk gene	GWAS
TYK2	Risk gene	GWAS
UBASH3A	Risk gene	GWAS
UBE2L3-YDJC	Risk gene	GWAS
VSIG4	Risk gene	GWAS
WDFY4	Risk gene	GWAS
ZFP36L1	Risk gene	GWAS
ZNF438	Risk gene	GWAS
ZNF689	Risk gene	GWAS
ACAT1	Both	GWAS

Gene	Type	Note
ACE2	Both	GWAS
ACHE	Both	GWAS
AHR	Both	GWAS
AKR1B1	Both	GWAS
AKR1B10	Both	GWAS
AKR1C1	Both	GWAS
ALOX5	Both	GWAS
ANXA1	Both	GWAS
ATIC	Both	GWAS
ATP4A	Both	GWAS
BCL2	Both	GWAS
C1QA	Both	GWAS
CA2	Both	GWAS
CA3	Both	GWAS
CDH11	Both	GWAS
DHFR	Both	GWAS
DHODH	Both	GWAS
FCGR2A	Both	GWAS
FKBP1A	Both	GWAS
HMGB1	Both	GWAS
PPARG	Both	GWAS
TNF	Both	GWAS
TNFSF11	Both	GWAS
TP53	Both	GWAS
TYMS	Both	GWAS
ADAMTS7	Biomarker	Biomarker Database
ADL	Biomarker	Biomarker Database
ADM	Biomarker	Biomarker Database
ADORA2A	Biomarker	Biomarker Database
AFP	Biomarker	Biomarker Database

Gene	Type	Note
AGA	Biomarker	Biomarker Database
AID	Biomarker	Biomarker Database
AIF1	Biomarker	Biomarker Database
AIM	Biomarker	Biomarker Database
AIRE	Biomarker	Biomarker Database
AKR1D1	Biomarker	Biomarker Database
ALKBH5	Biomarker	Biomarker Database
ALP	Biomarker	Biomarker Database
ALS	Biomarker	Biomarker Database
AMG	Biomarker	Biomarker Database
AMH	Biomarker	Biomarker Database
AMPD1	Biomarker	Biomarker Database
AMPK	Biomarker	Biomarker Database
ANA	Biomarker	Biomarker Database
ANGPTL4	Biomarker	Biomarker Database
ANO1	Biomarker	Biomarker Database
ANOVA	Biomarker	Biomarker Database
APB	Biomarker	Biomarker Database
APC	Biomarker	Biomarker Database
APN	Biomarker	Biomarker Database
APOH	Biomarker	Biomarker Database
APP	Biomarker	Biomarker Database
APR	Biomarker	Biomarker Database
APRIL	Biomarker	Biomarker Database
APS	Biomarker	Biomarker Database
ARA	Biomarker	Biomarker Database
ARS	Biomarker	Biomarker Database
ASC	Biomarker	Biomarker Database
ASH	Biomarker	Biomarker Database
ATG16L1	Biomarker	Biomarker Database

Gene	Type	Note
B3GNT2	Biomarker	Biomarker Database
B7H3	Biomarker	Biomarker Database
BAFF	Biomarker	Biomarker Database
BAP	Biomarker	Biomarker Database
BBP	Biomarker	Biomarker Database
BCMA	Biomarker	Biomarker Database
BCR	Biomarker	Biomarker Database
BECN1	Biomarker	Biomarker Database
BGM	Biomarker	Biomarker Database
BGN	Biomarker	Biomarker Database
BGP	Biomarker	Biomarker Database
BLC	Biomarker	Biomarker Database
BMP2	Biomarker	Biomarker Database
BMP7	Biomarker	Biomarker Database
BR3	Biomarker	Biomarker Database
BRAF	Biomarker	Biomarker Database
BTK	Biomarker	Biomarker Database
C5orf30	Biomarker	Biomarker Database
CA125	Biomarker	Biomarker Database
CAC	Biomarker	Biomarker Database
CAD	Biomarker	Biomarker Database
CAL	Biomarker	Biomarker Database
CAN	Biomarker	Biomarker Database
CAP	Biomarker	Biomarker Database
CAPG	Biomarker	Biomarker Database
CAR	Biomarker	Biomarker Database
CARF	Biomarker	Biomarker Database
CASC2	Biomarker	Biomarker Database
CAT	Biomarker	Biomarker Database
CBG	Biomarker	Biomarker Database

Gene	Type	Note
CCL11	Biomarker	Biomarker Database
CCL18	Biomarker	Biomarker Database
CCL19	Biomarker	Biomarker Database
CCL2	Biomarker	Biomarker Database
CCL21	Biomarker	Biomarker Database
CCL3	Biomarker	Biomarker Database
CCL4	Biomarker	Biomarker Database
CCL5	Biomarker	Biomarker Database
CCL7	Biomarker	Biomarker Database
CCN1	Biomarker	Biomarker Database
CCN2	Biomarker	Biomarker Database
CCN3	Biomarker	Biomarker Database
CCN4	Biomarker	Biomarker Database
CCP2	Biomarker	Biomarker Database
CCP3	Biomarker	Biomarker Database
CCR2	Biomarker	Biomarker Database
CCR4	Biomarker	Biomarker Database
CCR5	Biomarker	Biomarker Database
CCR6	Biomarker	Biomarker Database
CD135	Biomarker	Biomarker Database
CD14	Biomarker	Biomarker Database
CD163	Biomarker	Biomarker Database
CD18	Biomarker	Biomarker Database
CD19	Biomarker	Biomarker Database
CD1B	Biomarker	Biomarker Database
CD1D	Biomarker	Biomarker Database
CD20	Biomarker	Biomarker Database
CD200	Biomarker	Biomarker Database
CD24	Biomarker	Biomarker Database
CD26	Biomarker	Biomarker Database

Gene	Type	Note
CD27	Biomarker	Biomarker Database
CD28	Biomarker	Biomarker Database
CD2BP1	Biomarker	Biomarker Database
CD30	Biomarker	Biomarker Database
CD34	Biomarker	Biomarker Database
CD38	Biomarker	Biomarker Database
CD4	Biomarker	Biomarker Database
CD40	Biomarker	Biomarker Database
CD40L	Biomarker	Biomarker Database
CD44	Biomarker	Biomarker Database
CD46	Biomarker	Biomarker Database
CD47	Biomarker	Biomarker Database
CD5	Biomarker	Biomarker Database
CD55	Biomarker	Biomarker Database
CD59	Biomarker	Biomarker Database
CD68	Biomarker	Biomarker Database
CD69	Biomarker	Biomarker Database
CD72	Biomarker	Biomarker Database
CD74	Biomarker	Biomarker Database
CD80	Biomarker	Biomarker Database
CD83	Biomarker	Biomarker Database
CD84	Biomarker	Biomarker Database
CD86	Biomarker	Biomarker Database
CDA	Biomarker	Biomarker Database
CEA	Biomarker	Biomarker Database
CEBPD	Biomarker	Biomarker Database
CEL	Biomarker	Biomarker Database
CGA	Biomarker	Biomarker Database
CHI3L1	Biomarker	Biomarker Database
CIC	Biomarker	Biomarker Database

Gene	Type	Note
CIRP	Biomarker	Biomarker Database
CIZ1	Biomarker	Biomarker Database
CKIP1	Biomarker	Biomarker Database
CLEC2	Biomarker	Biomarker Database
CLI	Biomarker	Biomarker Database
CLP	Biomarker	Biomarker Database
CLU	Biomarker	Biomarker Database
CMTM1	Biomarker	Biomarker Database
CMTM2	Biomarker	Biomarker Database
CMTM3	Biomarker	Biomarker Database
CMTM6	Biomarker	Biomarker Database
CNR2	Biomarker	Biomarker Database
CNTF	Biomarker	Biomarker Database
COG6	Biomarker	Biomarker Database
COMP	Biomarker	Biomarker Database
COPD	Biomarker	Biomarker Database
CORO1A	Biomarker	Biomarker Database
COX2	Biomarker	Biomarker Database
CPA	Biomarker	Biomarker Database
CPM	Biomarker	Biomarker Database
CR1	Biomarker	Biomarker Database
CREST	Biomarker	Biomarker Database
CRIg	Biomarker	Biomarker Database
CRP	Biomarker	Biomarker Database
CSA	Biomarker	Biomarker Database
CSF1	Biomarker	Biomarker Database
CST	Biomarker	Biomarker Database
CTGF	Biomarker	Biomarker Database
CTHRC1	Biomarker	Biomarker Database
CTLA4	Biomarker	Biomarker Database

Gene	Type	Note
CTRL	Biomarker	Biomarker Database
CTSS	Biomarker	Biomarker Database
CX3CL1	Biomarker	Biomarker Database
CX3CR1	Biomarker	Biomarker Database
CXCL10	Biomarker	Biomarker Database
CXCL11	Biomarker	Biomarker Database
CXCL12	Biomarker	Biomarker Database
CXCL13	Biomarker	Biomarker Database
CXCL8	Biomarker	Biomarker Database
CXCR2	Biomarker	Biomarker Database
CXCR3	Biomarker	Biomarker Database
CXCR4	Biomarker	Biomarker Database
CXCR5	Biomarker	Biomarker Database
CYP3A4	Biomarker	Biomarker Database
CYP3A5	Biomarker	Biomarker Database
CYR61	Biomarker	Biomarker Database
DAP12	Biomarker	Biomarker Database
DBP	Biomarker	Biomarker Database
DCIR	Biomarker	Biomarker Database
DDAH	Biomarker	Biomarker Database
DDT	Biomarker	Biomarker Database
DED	Biomarker	Biomarker Database
DES	Biomarker	Biomarker Database
DHCR7	Biomarker	Biomarker Database
DICER1	Biomarker	Biomarker Database
DIS	Biomarker	Biomarker Database
DKK 1.00	Biomarker	Biomarker Database
DMA	Biomarker	Biomarker Database
DMD	Biomarker	Biomarker Database
DNASE2	Biomarker	Biomarker Database

Gene	Type	Note
DOCK2	Biomarker	Biomarker Database
DR1	Biomarker	Biomarker Database
DR3	Biomarker	Biomarker Database
DR4	Biomarker	Biomarker Database
DRB1	Biomarker	Biomarker Database
DREAM	Biomarker	Biomarker Database
ECM	Biomarker	Biomarker Database
EGF	Biomarker	Biomarker Database
ELMO1	Biomarker	Biomarker Database
END	Biomarker	Biomarker Database
ENO1	Biomarker	Biomarker Database
ENTPD1	Biomarker	Biomarker Database
ERAS	Biomarker	Biomarker Database
ERK	Biomarker	Biomarker Database
ESR	Biomarker	Biomarker Database
ETA	Biomarker	Biomarker Database
FAC	Biomarker	Biomarker Database
FADD	Biomarker	Biomarker Database
FAP	Biomarker	Biomarker Database
FCN1	Biomarker	Biomarker Database
FCRL5	Biomarker	Biomarker Database
FGF12	Biomarker	Biomarker Database
FGF2	Biomarker	Biomarker Database
FGF21	Biomarker	Biomarker Database
FGFR1	Biomarker	Biomarker Database
FGL2	Biomarker	Biomarker Database
FKBP13	Biomarker	Biomarker Database
FKN	Biomarker	Biomarker Database
FMS	Biomarker	Biomarker Database
FMT	Biomarker	Biomarker Database

Gene	Type	Note
FOXO1	Biomarker	Biomarker Database
FOXO3	Biomarker	Biomarker Database
FOXO4	Biomarker	Biomarker Database
FPGS	Biomarker	Biomarker Database
FSA	Biomarker	Biomarker Database
FSTL1	Biomarker	Biomarker Database
FTO	Biomarker	Biomarker Database
FUR	Biomarker	Biomarker Database
FURIN	Biomarker	Biomarker Database
G0S2	Biomarker	Biomarker Database
G3BP	Biomarker	Biomarker Database
GAD	Biomarker	Biomarker Database
GADD34	Biomarker	Biomarker Database
GAK	Biomarker	Biomarker Database
GAPDH	Biomarker	Biomarker Database
GCA	Biomarker	Biomarker Database
GCF	Biomarker	Biomarker Database
GCSF	Biomarker	Biomarker Database
GDF11	Biomarker	Biomarker Database
GDF15	Biomarker	Biomarker Database
GFR	Biomarker	Biomarker Database
GGH	Biomarker	Biomarker Database
GITR	Biomarker	Biomarker Database
GITRL	Biomarker	Biomarker Database
GLS	Biomarker	Biomarker Database
GLUT3	Biomarker	Biomarker Database
GMCSF	Biomarker	Biomarker Database
GNG13	Biomarker	Biomarker Database
GPA	Biomarker	Biomarker Database
GPC	Biomarker	Biomarker Database

Gene	Type	Note
GPI	Biomarker	Biomarker Database
GPX1	Biomarker	Biomarker Database
GRS	Biomarker	Biomarker Database
GSDMB	Biomarker	Biomarker Database
GSN	Biomarker	Biomarker Database
GSS	Biomarker	Biomarker Database
GTF2I	Biomarker	Biomarker Database
GZMB	Biomarker	Biomarker Database
H2AX	Biomarker	Biomarker Database
HAP	Biomarker	Biomarker Database
HBD	Biomarker	Biomarker Database
HFE	Biomarker	Biomarker Database
HGF	Biomarker	Biomarker Database
HIF1	Biomarker	Biomarker Database
HLAA	Biomarker	Biomarker Database
HLADPB1	Biomarker	Biomarker Database
HLADQB1	Biomarker	Biomarker Database
HLADRB1	Biomarker	Biomarker Database
HLADRB5	Biomarker	Biomarker Database
HLAG	Biomarker	Biomarker Database
HO1	Biomarker	Biomarker Database
HPA	Biomarker	Biomarker Database
HSC	Biomarker	Biomarker Database
HSP47	Biomarker	Biomarker Database
HSP60	Biomarker	Biomarker Database
HSPA1A	Biomarker	Biomarker Database
HYA	Biomarker	Biomarker Database
IBD	Biomarker	Biomarker Database
ICAM1	Biomarker	Biomarker Database
ICOS	Biomarker	Biomarker Database

Gene	Type	Note
ICOSL	Biomarker	Biomarker Database
ID1	Biomarker	Biomarker Database
IDO1	Biomarker	Biomarker Database
IDO2	Biomarker	Biomarker Database
IFI16	Biomarker	Biomarker Database
IFI44L	Biomarker	Biomarker Database
IGF1	Biomarker	Biomarker Database
IGFBP3	Biomarker	Biomarker Database
IGHG3	Biomarker	Biomarker Database
IL10	Biomarker	Biomarker Database
IL11	Biomarker	Biomarker Database
IL13	Biomarker	Biomarker Database
IL15	Biomarker	Biomarker Database
IL16	Biomarker	Biomarker Database
IL17	Biomarker	Biomarker Database
IL17A	Biomarker	Biomarker Database
IL17F	Biomarker	Biomarker Database
IL17RA	Biomarker	Biomarker Database
IL18	Biomarker	Biomarker Database
IL18BP	Biomarker	Biomarker Database
IL1RA	Biomarker	Biomarker Database
IL2	Biomarker	Biomarker Database
IL20RB	Biomarker	Biomarker Database
IL21	Biomarker	Biomarker Database
IL22	Biomarker	Biomarker Database
IL23R	Biomarker	Biomarker Database
IL25	Biomarker	Biomarker Database
IL26	Biomarker	Biomarker Database
IL27	Biomarker	Biomarker Database
IL29	Biomarker	Biomarker Database

Gene	Type	Note
IL32	Biomarker	Biomarker Database
IL33	Biomarker	Biomarker Database
IL34	Biomarker	Biomarker Database
IL37	Biomarker	Biomarker Database
IL38	Biomarker	Biomarker Database
IL4	Biomarker	Biomarker Database
IL4R	Biomarker	Biomarker Database
IL5	Biomarker	Biomarker Database
IL6	Biomarker	Biomarker Database
IL6R	Biomarker	Biomarker Database
IL7	Biomarker	Biomarker Database
IL7R	Biomarker	Biomarker Database
IL8	Biomarker	Biomarker Database
INA	Biomarker	Biomarker Database
IPEX	Biomarker	Biomarker Database
IPP	Biomarker	Biomarker Database
IRAK	Biomarker	Biomarker Database
IRAK1	Biomarker	Biomarker Database
IRF5	Biomarker	Biomarker Database
ITAC	Biomarker	Biomarker Database
ITGA4	Biomarker	Biomarker Database
ITGAM	Biomarker	Biomarker Database
ITGB2	Biomarker	Biomarker Database
ITIH3	Biomarker	Biomarker Database
ITPA	Biomarker	Biomarker Database
JAK1	Biomarker	Biomarker Database
JAK2	Biomarker	Biomarker Database
JAK3	Biomarker	Biomarker Database
KDR	Biomarker	Biomarker Database
KIAA1199	Biomarker	Biomarker Database

Gene	Type	Note
KRAS	Biomarker	Biomarker Database
LAP	Biomarker	Biomarker Database
LBP	Biomarker	Biomarker Database
LCN2	Biomarker	Biomarker Database
LDLC	Biomarker	Biomarker Database
LEDGF	Biomarker	Biomarker Database
LIF	Biomarker	Biomarker Database
LINC00305	Biomarker	Biomarker Database
LOR	Biomarker	Biomarker Database
LOX	Biomarker	Biomarker Database
LOX1	Biomarker	Biomarker Database
LPA	Biomarker	Biomarker Database
LPD	Biomarker	Biomarker Database
LRG	Biomarker	Biomarker Database
LRRC31	Biomarker	Biomarker Database
LRRK2	Biomarker	Biomarker Database
LTB	Biomarker	Biomarker Database
LY96	Biomarker	Biomarker Database
MAF	Biomarker	Biomarker Database
MAGEB2	Biomarker	Biomarker Database
MAP	Biomarker	Biomarker Database
MAPT	Biomarker	Biomarker Database
MASP2	Biomarker	Biomarker Database
MBL	Biomarker	Biomarker Database
MBL2	Biomarker	Biomarker Database
MBP	Biomarker	Biomarker Database
MCI	Biomarker	Biomarker Database
MCP	Biomarker	Biomarker Database
MCP1	Biomarker	Biomarker Database
MCPIP	Biomarker	Biomarker Database

Gene	Type	Note
MDA5	Biomarker	Biomarker Database
MDR1	Biomarker	Biomarker Database
MEKK1	Biomarker	Biomarker Database
MEKK2	Biomarker	Biomarker Database
MEKK3	Biomarker	Biomarker Database
METRNL	Biomarker	Biomarker Database
METTL3	Biomarker	Biomarker Database
MFAP4	Biomarker	Biomarker Database
MFGE8	Biomarker	Biomarker Database
MFI	Biomarker	Biomarker Database
MIA	Biomarker	Biomarker Database
MIC1	Biomarker	Biomarker Database
MICA	Biomarker	Biomarker Database
MIF	Biomarker	Biomarker Database
MIG	Biomarker	Biomarker Database
MIP2	Biomarker	Biomarker Database
mir10a	Biomarker	Biomarker Database
mir125a	Biomarker	Biomarker Database
mir132	Biomarker	Biomarker Database
mir140	Biomarker	Biomarker Database
mir146a	Biomarker	Biomarker Database
mir155	Biomarker	Biomarker Database
mir15a	Biomarker	Biomarker Database
mir200c	Biomarker	Biomarker Database
mir21	Biomarker	Biomarker Database
mir210	Biomarker	Biomarker Database
mir212	Biomarker	Biomarker Database
mir223	Biomarker	Biomarker Database
mir23b	Biomarker	Biomarker Database
mir26b	Biomarker	Biomarker Database

Gene	Type	Note
mir29a	Biomarker	Biomarker Database
mir3168	Biomarker	Biomarker Database
mir363	Biomarker	Biomarker Database
mir424	Biomarker	Biomarker Database
mir497	Biomarker	Biomarker Database
mir498	Biomarker	Biomarker Database
mir5100	Biomarker	Biomarker Database
mir98	Biomarker	Biomarker Database
MLKL	Biomarker	Biomarker Database
MLN	Biomarker	Biomarker Database
MLR	Biomarker	Biomarker Database
MMP1	Biomarker	Biomarker Database
MMP2	Biomarker	Biomarker Database
MMP3	Biomarker	Biomarker Database
MMP8	Biomarker	Biomarker Database
MMP9	Biomarker	Biomarker Database
MPO	Biomarker	Biomarker Database
MPS1	Biomarker	Biomarker Database
MRI	Biomarker	Biomarker Database
MRP	Biomarker	Biomarker Database
MRP1	Biomarker	Biomarker Database
MRP14	Biomarker	Biomarker Database
MRP8	Biomarker	Biomarker Database
MSC	Biomarker	Biomarker Database
MTC	Biomarker	Biomarker Database
MTHFD1	Biomarker	Biomarker Database
MTHFR	Biomarker	Biomarker Database
MTMR3	Biomarker	Biomarker Database
MTRR	Biomarker	Biomarker Database
MTX	Biomarker	Biomarker Database

Gene	Type	Note
MUC7	Biomarker	Biomarker Database
MZB1	Biomarker	Biomarker Database
NAC	Biomarker	Biomarker Database
NAG	Biomarker	Biomarker Database
NAMPT	Biomarker	Biomarker Database
NAT2	Biomarker	Biomarker Database
NCC	Biomarker	Biomarker Database
NCF1	Biomarker	Biomarker Database
NEFA	Biomarker	Biomarker Database
NET	Biomarker	Biomarker Database
NFKBIE	Biomarker	Biomarker Database
NGF	Biomarker	Biomarker Database
NHL	Biomarker	Biomarker Database
NHS	Biomarker	Biomarker Database
NKB1	Biomarker	Biomarker Database
NKG2D	Biomarker	Biomarker Database
NLRP3	Biomarker	Biomarker Database
NMA	Biomarker	Biomarker Database
NPY	Biomarker	Biomarker Database
NSP	Biomarker	Biomarker Database
NTM	Biomarker	Biomarker Database
OCR	Biomarker	Biomarker Database
OCT	Biomarker	Biomarker Database
OLIG3	Biomarker	Biomarker Database
OMP	Biomarker	Biomarker Database
OPG	Biomarker	Biomarker Database
OPN	Biomarker	Biomarker Database
OSM	Biomarker	Biomarker Database
PACAP	Biomarker	Biomarker Database
PAD2	Biomarker	Biomarker Database

Gene	Type	Note
PAD4	Biomarker	Biomarker Database
PADI4	Biomarker	Biomarker Database
PAH	Biomarker	Biomarker Database
PAI1	Biomarker	Biomarker Database
PAI2	Biomarker	Biomarker Database
PAP	Biomarker	Biomarker Database
PAR2	Biomarker	Biomarker Database
PARC	Biomarker	Biomarker Database
PD1	Biomarker	Biomarker Database
PDCD5	Biomarker	Biomarker Database
PDIA3	Biomarker	Biomarker Database
PDL1	Biomarker	Biomarker Database
PDL2	Biomarker	Biomarker Database
PDPN	Biomarker	Biomarker Database
PDS	Biomarker	Biomarker Database
PF4	Biomarker	Biomarker Database
PGD	Biomarker	Biomarker Database
PGF	Biomarker	Biomarker Database
PGLYRP1	Biomarker	Biomarker Database
PIP	Biomarker	Biomarker Database
PLP	Biomarker	Biomarker Database
PML	Biomarker	Biomarker Database
PON	Biomarker	Biomarker Database
PON1	Biomarker	Biomarker Database
PPAR	Biomarker	Biomarker Database
PPD	Biomarker	Biomarker Database
PPM1A	Biomarker	Biomarker Database
PRF1	Biomarker	Biomarker Database
PRKCH	Biomarker	Biomarker Database
PRL	Biomarker	Biomarker Database

Gene	Type	Note
PSO	Biomarker	Biomarker Database
PTH	Biomarker	Biomarker Database
PTPN2	Biomarker	Biomarker Database
PTPN22	Biomarker	Biomarker Database
PTPRC	Biomarker	Biomarker Database
PTX3	Biomarker	Biomarker Database
PUFA	Biomarker	Biomarker Database
PUMA	Biomarker	Biomarker Database
RAF	Biomarker	Biomarker Database
RAGE	Biomarker	Biomarker Database
RANK	Biomarker	Biomarker Database
RANKL	Biomarker	Biomarker Database
RBP4	Biomarker	Biomarker Database
REM	Biomarker	Biomarker Database
RFC1	Biomarker	Biomarker Database
RIP	Biomarker	Biomarker Database
RIPK1	Biomarker	Biomarker Database
RIS	Biomarker	Biomarker Database
RNP	Biomarker	Biomarker Database
ROS	Biomarker	Biomarker Database
RPC	Biomarker	Biomarker Database
S100A11	Biomarker	Biomarker Database
S100A12	Biomarker	Biomarker Database
S100A4	Biomarker	Biomarker Database
S100A8	Biomarker	Biomarker Database
S100A9	Biomarker	Biomarker Database
S1P	Biomarker	Biomarker Database
SAHH	Biomarker	Biomarker Database
SAP	Biomarker	Biomarker Database
SBP	Biomarker	Biomarker Database

Gene	Type	Note
SCD	Biomarker	Biomarker Database
SCF	Biomarker	Biomarker Database
SDC	Biomarker	Biomarker Database
SDC1	Biomarker	Biomarker Database
SDF1	Biomarker	Biomarker Database
SERS	Biomarker	Biomarker Database
SFL	Biomarker	Biomarker Database
SHBG	Biomarker	Biomarker Database
SHMT1	Biomarker	Biomarker Database
SIGIRR	Biomarker	Biomarker Database
SIL	Biomarker	Biomarker Database
SLA	Biomarker	Biomarker Database
SLC11A1	Biomarker	Biomarker Database
SLC19A1	Biomarker	Biomarker Database
SLC2A3	Biomarker	Biomarker Database
SLC7A5	Biomarker	Biomarker Database
SMS2	Biomarker	Biomarker Database
SNX9	Biomarker	Biomarker Database
SOST	Biomarker	Biomarker Database
SPR	Biomarker	Biomarker Database
SQSTM1	Biomarker	Biomarker Database
SRP	Biomarker	Biomarker Database
SRR	Biomarker	Biomarker Database
ST2	Biomarker	Biomarker Database
STAT	Biomarker	Biomarker Database
STAT1	Biomarker	Biomarker Database
STAT3	Biomarker	Biomarker Database
STAT4	Biomarker	Biomarker Database
STM	Biomarker	Biomarker Database
SYNGR1	Biomarker	Biomarker Database

Gene	Type	Note
TACE	Biomarker	Biomarker Database
TACI	Biomarker	Biomarker Database
TAK1	Biomarker	Biomarker Database
TAS2R10	Biomarker	Biomarker Database
TAS2R13	Biomarker	Biomarker Database
TAS2R14	Biomarker	Biomarker Database
TAS2R19	Biomarker	Biomarker Database
TAS2R20	Biomarker	Biomarker Database
TAS2R43	Biomarker	Biomarker Database
TAS2R45	Biomarker	Biomarker Database
TAS2R46	Biomarker	Biomarker Database
TAT	Biomarker	Biomarker Database
TBX3	Biomarker	Biomarker Database
TEK	Biomarker	Biomarker Database
TFPI	Biomarker	Biomarker Database
TGFBR2	Biomarker	Biomarker Database
TGT	Biomarker	Biomarker Database
THBS1	Biomarker	Biomarker Database
TIA1	Biomarker	Biomarker Database
TIE	Biomarker	Biomarker Database
TIM3	Biomarker	Biomarker Database
TIMM23	Biomarker	Biomarker Database
TIMP	Biomarker	Biomarker Database
TIMP1	Biomarker	Biomarker Database
TIP1	Biomarker	Biomarker Database
TLR10	Biomarker	Biomarker Database
TLR2	Biomarker	Biomarker Database
TLR3	Biomarker	Biomarker Database
TLR4	Biomarker	Biomarker Database
TLR5	Biomarker	Biomarker Database

Gene	Type	Note
TLR7	Biomarker	Biomarker Database
TNC	Biomarker	Biomarker Database
TNFAIP3	Biomarker	Biomarker Database
TNFR1	Biomarker	Biomarker Database
TNFR2	Biomarker	Biomarker Database
TNFRSF11B	Biomarker	Biomarker Database
TNFSF10	Biomarker	Biomarker Database
TNIP1	Biomarker	Biomarker Database
TPO	Biomarker	Biomarker Database
TRAF1	Biomarker	Biomarker Database
TRAF6	Biomarker	Biomarker Database
TRAIL	Biomarker	Biomarker Database
TRANCE	Biomarker	Biomarker Database
TREM1	Biomarker	Biomarker Database
TRP	Biomarker	Biomarker Database
TRX	Biomarker	Biomarker Database
TSLP	Biomarker	Biomarker Database
TSP	Biomarker	Biomarker Database
TSP1	Biomarker	Biomarker Database
TSPAN33	Biomarker	Biomarker Database
TSPO	Biomarker	Biomarker Database
TST	Biomarker	Biomarker Database
TTP	Biomarker	Biomarker Database
TTR	Biomarker	Biomarker Database
TXN	Biomarker	Biomarker Database
VCAM1	Biomarker	Biomarker Database
VDR	Biomarker	Biomarker Database
VEGF	Biomarker	Biomarker Database
VEGFA	Biomarker	Biomarker Database
VEGFC	Biomarker	Biomarker Database

Gene	Type	Note
VEGFR1	Biomarker	Biomarker Database
VIP	Biomarker	Biomarker Database
VWF	Biomarker	Biomarker Database
WNT5A	Biomarker	Biomarker Database
XDH	Biomarker	Biomarker Database
YTHDF2	Biomarker	Biomarker Database
YY1	Biomarker	Biomarker Database
ZAP70	Biomarker	Biomarker Database
